# Elevated inflammatory biomarkers and poor outcomes in intracerebral hemorrhage

**DOI:** 10.1007/s00415-022-11284-8

**Published:** 2022-07-22

**Authors:** Edward R. Bader, Tiberiu A. Pana, Raphae S. Barlas, Anthony K. Metcalf, John F. Potter, Phyo K. Myint

**Affiliations:** 1 Department of Neurological Surgery, Albert Einstein College of Medicine, 1410 Pelham Parkway South, Rose F. Kennedy Center, Bronx, NY 10461 USA; 2 Institute of Applied Health Sciences, School of Medicine, Medical Sciences and Nutrition, University of Aberdeen, Foresthill, Aberdeen, UK; 3 Norfolk and Norwich University Hospital, Stroke Research Group, Norwich, Norfolk UK; 4 Department of Ageing and Stroke Medicine, University of East Anglia, Norwich, Norfolk UK

**Keywords:** Intracerebral hemorrhage, Stroke, Prognosis, Outcomes, Inflammation, Biomarkers

## Abstract

**Background:**

Accumulating evidence suggests that spontaneous intracerebral hemorrhage (ICH) is associated with a reactive neuroinflammatory response. However, it remains unclear if circulating inflammatory biomarkers are associated with adverse outcomes in ICH. To address this knowledge gap, we conducted a cohort study using a prospectively maintained stroke register in the United Kingdom to assess the prognostic value of admission inflammatory biomarkers in ICH.

**Methods:**

The Norfolk and Norwich Stroke and TIA Register recorded consecutive ICH cases. The primary exposures of interest were elevation of white cell count (WCC; > 10 × 10^9^/L), elevation of c-reactive protein (CRP; > 10 mg/L), and co-elevation of both biomarkers, at the time of admission. Modified Poisson and Cox regressions were conducted to investigate the relationship between co-elevation of WCC and CRP at admission and outcomes following ICH. Functional outcome, multiple mortality timepoints, and length of stay were assessed.

**Results:**

In total, 1714 ICH cases were identified from the register. After adjusting for covariates, including stroke-associated pneumonia, co-elevation of WCC and CRP at admission was independently associated with significantly increased risk of poor functional outcome (RR 1.08 [95% CI 1.01–1.15]) and inpatient mortality (RR 1.21 [95% CI 1.06–1.39]); and increased 90-day (HR 1.22 [95% CI 1.03–1.45]), and 1-year mortality (HR 1.20 [95% CI 1.02–1.41]). Individual elevation of WCC or CRP was also associated with poor outcomes.

**Conclusions:**

Elevated inflammatory biomarkers were associated with poor outcomes in ICH. This study indicates that these readily available biomarkers may be valuable for prognostication and underscore the importance of inflammation in ICH.

**Supplementary Information:**

The online version contains supplementary material available at 10.1007/s00415-022-11284-8.

## Introduction

Spontaneous intracerebral hemorrhage (ICH) continues to be associated with high rates of morbidity and mortality, and there remains no definitive treatment beyond supportive care[1, 2]. Accumulating experimental evidence demonstrates that intracerebral hemorrhage drives a microglia and T-cell-mediated inflammatory reaction in the brain[3]. This inflammatory response is associated with perihematomal oedema, cytokine release, and breakdown of the blood–brain barrier with resultant leukocyte influx[4], and may contribute to poor outcomes following ICH.

The extent of inflammation has been shown to correlate with ICH severity experimentally [5, 6], suggesting that clinical markers of inflammation may be associated with adverse outcomes after ICH. However, studies on this topic remain limited to date and, critically, have not accounted for the potential confounding arising from stroke-associated pneumonia (SAP) [7, 8]. Additionally, targeting post-hemorrhagic inflammation is an appealing avenue for the development of future treatments. Such treatments may require selection of appropriate candidates for intervention: inflammatory biomarkers may therefore provide a rapid and readily accessible means of triage.

There is, therefore, a need to understand the relationship between clinically identifiable inflammation and subsequent outcomes in ICH populations. To address this, we performed a cohort study using a prospectively maintained stroke register of consecutive admissions in the United Kingdom to investigate the relationship between elevated inflammatory blood biomarkers at the time of admission and outcomes in ICH.

## Methods

### Database and patient identification

The Norfolk and Norwich Stroke and TIA register is a prospectively maintained stroke register in the United Kingdom that records all stroke admissions to the Norfolk and Norwich University Hospital, which had a catchment of approximately 900,000 in 2017. The Newcastle and Tyneside National Health Service and Research Ethics Committee provided ethical approval for this database (17/NE/0277), providing approval for anonymized research studies, and waiving the requirement for individual patient consent. There are no other tertiary care centers in Norfolk County, ensuring a high percentage capture of stroke cases. Full data collection methods have been previously published in detail [9]. Data from multiple sources are combined for each patient to provide a wide range of variables for each ICH case; specialist nurses determine the pre-stroke modified Rankin Score (mRS) for each patient. Patients were included if they were admitted with a diagnosis of spontaneous non-traumatic ICH and had complete follow-up data between inclusion in the study and the end of the follow-up period in June 2017.

### Data extraction and exposures

Baseline patient demographics were extracted from the database, along with relevant covariates and comorbidities. Data extracted were as follows: age, sex, pre-ICH mRS, Oxford Community Stroke Project classification (OCSP), prescriptions at admission (anticoagulants and antiplatelets), and stroke-associated pneumonia during admission (SAP; defined as any pneumonia within 7 days of admission[10]). All pre-ICH comorbidities recorded in the database were extracted using the International Classification of Disease-tenth edition (ICD-10): asthma (J45), atrial fibrillation (I48), previous cerebral infarction (I63), coronary heart disease (I20–I25), congestive heart failure (I50), chronic kidney disease (N18), chronic obstructive pulmonary disorder—COPD (J40–J44, J47), dementia (F01–F05), diabetes mellitus (E10–E14), hyperlipidemia (E78), hypertension (I10–I15), liver disease (K70–K77), malignancy (C00–C97), peptic ulcer disease (K25–K28), peripheral vascular disease (I73.9), previous subarachnoid (SAH) or intracerebral hemorrhage (I60–I61), and rheumatoid arthritis/connective tissue disease (M32, M34, M332, M053, M058, M059, M060, M063, M069, M050, M052, M051, M353).

Admission values for undifferentiated white cell count (WCC), and C-reactive protein (CRP) blood tests were extracted. The primary exposure of interest was co-elevation of WCC (> 10 × 10^9^/L) and CRP (> 10 mg/L) at the time of admission. These thresholds were selected as they have been validated for clinical purposes at the study center, defined as the upper bound for the normal range [11, 12]. A combined exposure using both biomarkers was used to provide a more comprehensive assay of the inflammatory state when compared with a single biomarker in isolation. Analyses of the associations of isolated elevated WCC (> 10 × 10^9^/L) and elevated CRP (> 10 mg/L) were also performed.

### Outcomes

The following outcomes were recorded: mortality, functional outcome, and length of stay. Mortality was assessed at multiple timepoints: inpatient mortality, 90-day mortality, and 1-year mortality. Functional outcome was assessed at discharge and recorded using the modified Rankin Scale. Functional outcome was dichotomized into good functional outcome (mRS 0–2 at discharge) and poor functional outcome (mRS 3–6 at discharge). Prolonged length of stay was defined as hospitalization > 14 days. Prolonged hospitalization is an important outcome to ascertain, as it provides useful information for service provision including economic considerations. This is particularly important in the UK NHS setting of universal healthcare in which the ability to estimate the length of hospitalization based on clinical characteristics is essential for to ensure equitable allocation of resources.

### Missing data and exclusions

**Fig. 1:**
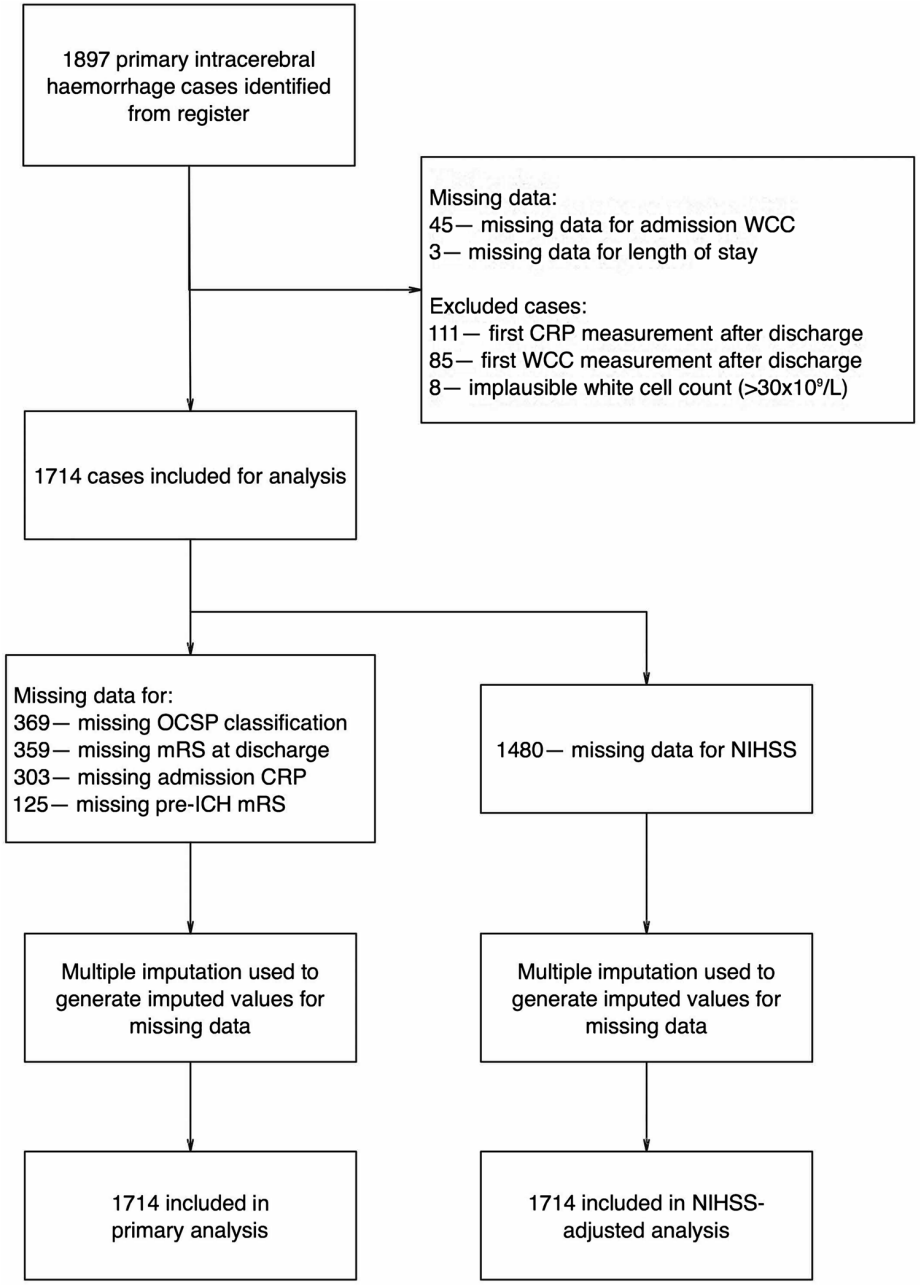
Flow chart of patient inclusion in analyses and handling of missing data. A total of 1714 patients were included following exclusions

Figure 1 summarizes identification of patients from the register. Missing data were handled in a predefined manner. Variables with < 5% missing data (WCC, LoS) were analyzed on a complete-case-analysis basis, while variables with ≥ 5% (mRS before ICH, mRS at discharge, CRP on admission, OCSP classification, and NIHSS) were imputed. Out of 1897 ICH admissions extracted from the register, the following patients were excluded: those with missing WCC (*n* = 45) or LoS (*n* = 3) data, those for whom the first measurement of WCC (*n* = 85) or CRP (*n* = 111) recorded in the registry occurred after discharge, and those with extreme or implausible WBC > 30 × 10^9^/L (*n* = 8), resulting in an included population of 1714 patients. Figure 1 also summarizes key variables with missing data after the application of the selection criteria: mRS before incident ICH (7.29% missing), CRP on admission (17.68% missing), mRS at discharge (20.95% missing), OCSP classification (21.53% missing), and NIHSS (86.35% missing). Missing data analysis revealed that patients with missing data on these variables were more significantly likely to have higher WCC, less likely to have pre-existing comorbidities on admission, and more likely to die after stroke (Tables S1.1–1.5). Data missingness was therefore likely to depend on observed factors, and therefore deemed likely to be missing-at-random[13]. A multiple imputation by chained equation (MICE) algorithm with 20 iterations was therefore implemented to impute the missing data. All variables were imputed using predictive mean matching drawing from five nearest neighbors. Conditional imputation was employed for mRS at discharge, only imputing this variable for patients discharged alive and restricting the imputation values to 0–5. Age, sex, prevalent comorbidities, SAP, admission anticoagulant and antiplatelet medications were used as predictors. A separate MICE algorithm was employed for the variables utilized for the long-term mortality analyses, replicating the methodology above, but also including the Nelson–Aalen estimator as a predictor.

As the NIHSS score was only routinely recorded in the register from 2015, there was a high degree of missingness for this variable. Therefore, sensitivity analyses replicating all the main regression models additionally adjusting for the imputed NIHSS were conducted. These did not show any meaningful differences in results (Tables 2, 3).

### Statistical analyses

Statistical analyses were performed using STATA 14.1 (STATA Corporation, 2015). As CRP was one of the imputed variables and was also used to define the combined exposure variable, descriptive statistics comparing the patient population characteristics by defined exposure were performed on the 20 imputed datasets obtained after MICE. Table 1 in the main manuscript details the descriptive statistics performed for one of the imputed datasets, while the other 19 are presented in Supplementary Tables (Tables S2.1–S2.20). For all 20 imputed datasets, the Pearson’s Chi-square test was used to compare categorical variables. The independent *t*-test was used to compare normally distributed continuous variables (age), and the Mann–Whitney *U* test to compare non-normally distributed continuous variables (NIHSS score, white cell count, C-reactive protein, and length of stay). A significance threshold of *P* < 0.05 was used.

**Table 1:** Cohort characteristics stratified by inflammatory biomarker status

Variable	ICH admissions			*P *value
	Total cohort (*n* = 1714)	Non-elevated inflammatory biomarkers (n = 1242)	Elevated inflammatory biomarkers (n = 472)	
Age—mean (± SD)	76.05 (12.25)	76.01 (12.32)	76.16 (12.07)	0.829
Sex—no. male (%)	854 (49.82)	642 (51.69)	212 (44.92)	**0.012**
Total NIHSS score—median (IQR)	5.00 (2.00–10.00)	5.00 (2.00–10.00)	5.00 (2.00–10.00)	0.527
*Pre-ICH mRS*				
0—no. (%)	1135 (66.22)	837 (67.39)	298 (63.14)	**0.005**
1—no. (%)	188 (10.97)	137 (11.03)	51 (10.81)	**0.005**
2—no. (%)	129 (7.53)	103 (8.29)	26 (5.51)	**0.005**
3—no. (%)	168 (9.8)	108 (8.7)	60 (12.71)	**0.005**
4—no. (%)	69 (4.03)	41 (3.3)	28 (5.93)	**0.005**
5—no. (%)	25 (1.46)	16 (1.29)	9 (1.91)	**0.005**
*OCSP classification*				
TACS	561 (32.73)	371 (29.87)	190 (40.25)	**< 0.001**
PACS	494 (28.82)	365 (29.39)	129 (27.33)	**< 0.001**
LACS	138 (8.05)	104 (8.37)	34 (7.2)	**< 0.001**
POCS	521 (30.4)	402 (32.37)	119 (25.21)	**< 0.001**
*Comorbidities*				
Asthma—no. (%)	146 (8.52)	114 (9.18)	32 (6.78)	0.112
Atrial fibrillation—no. (%)	383 (22.35)	268 (21.58)	115 (24.36)	0.216
Cerebral infarction—no. (%)	135 (7.88)	99 (7.97)	36 (7.63)	0.813
Coronary heart disease—no. (%)	327 (19.08)	219 (17.63)	108 (22.88)	**0.013**
Congestive heart failure—no. (%)	135 (7.88)	86 (6.92)	49 (10.38)	**0.018**
Chronic kidney disease—no. (%)	85 (4.96)	57 (4.59)	28 (5.93)	0.253
COPD—no. (%)	104 (6.07)	80 (6.44)	24 (5.08)	0.293
Dementia—no. (%)	71 (4.14)	51 (4.11)	20 (4.24)	0.903
Diabetes mellitus—no. (%)	205 (11.96)	148 (11.92)	57 (12.08)	0.927
Hyperlipidemia—no. (%)	179 (10.44)	135 (10.87)	44 (9.32)	0.349
Hypertension—no. (%)	996 (58.11)	730 (58.78)	266 (56.36)	0.364
Liver disease—no. (%)	26 (1.52)	23 (1.85)	3 (.64)	0.066
Malignancy—no. (%)	243 (14.18)	178 (14.33)	65 (13.77)	0.766
Peptic ulcer disease—no. (%)	64 (3.73)	43 (3.46)	21 (4.45)	0.336
Peripheral vascular disease—no. (%)	82 (4.78)	60 (4.83)	22 (4.66)	0.883
Previous ICH/SAH—no. (%)	377 (22)	282 (22.71)	95 (20.13)	0.250
Connective tissue disease—no. (%)	62 (3.62)	47 (3.78)	15 (3.18)	0.548
Stroke-associated pneumonia—no. (%)	194 (11.32)	119 (9.58)	75 (15.89)	**< 0.001**
*Admission medications*				
Anticoagulants—no. (%)	34 (1.98)	29 (2.33)	5 (1.06)	0.091
Antiplatelets—no. (%)	604 (35.24)	429 (34.54)	175 (37.08)	0.326
*Biomarkers at admission*				
White cell count (× 10^9/L)—median (IQR)	9.90 (7.60–12.60)	8.70 (7.00–10.60)	12.95 (11.30–16.25)	**< 0.001**
CRP (mg/L)—median (IQR)	10.00 (4.00–27.00)	7.00 (4.00–14.00)	27.00 (14.00–57.00)	**< 0.001**
*Outcomes*				
Poor functional outcome—no. (%)	1319 (76.95)	925 (74.48)	394 (83.47)	**< 0.001**
*mRS at discharge*				
Discharge mRS 0—no. (%)	156 (9.1)	120 (9.66)	36 (7.63)	**< 0.001**
Discharge mRS 1—no. (%)	149 (8.69)	126 (10.14)	23 (4.87)	**< 0.001**
Discharge mRS 2—no. (%)	90 (5.25)	71 (5.72)	19 (4.03)	**< 0.001**
Discharge mRS 3—no. (%)	238 (13.89)	172 (13.85)	66 (13.98)	**< 0.001**
Discharge mRS 4—no. (%)	312 (18.2)	233 (18.76)	79 (16.74)	**< 0.001**
Discharge mRS 5—no. (%)	146 (8.52)	107 (8.62)	39 (8.26)	**< 0.001**
Discharge mRS 6—no. (%)	623 (36.35)	413 (33.25)	210 (44.49)	**< 0.001**
*Mortality*				
Death during admission—no. (%)	623 (36.35)	413 (33.25)	210 (44.49)	**< 0.001**
Death at 90 days—no. (%)	714 (41.66)	480 (38.65)	234 (49.58)	**< 0.001**
Death at 365 days—no. (%)	834 (48.66)	568 (45.73)	266 (56.36)	**< 0.001**
*Length of stay*				
Days—median (IQR)	8.00 (3.00–19.00)	8.00 (3.00–19.00)	8.00 (3.00–23.00)	0.068
Length of stay > 14 days—no. (%)	576 (33.61)	396 (31.88)	180 (38.14)	**0.014**

Elevated inflammatory biomarkers was defined as white cell count > 10 × 10^9^/L and C-reactive protein > 10 mg/L

As C-reactive protein (CRP) data contained 17.68% missing data, missing values imputed using a multiple imputation by chained equations algorithm with 20 resulting imputations. As the definition of exposure variable includes CRP, the descriptive statistics comparing the patient population characteristics by defined exposure were performed on each one of the 20 imputed datasets thus obtained. The descriptive statistics display above were performed for one of the imputed datasets, while the other 19 are presented in Supplementary Tables (Tables S2.1–S2.20)

The Pearson’s Chi-square test was used to compare categorical variables. The independent t-test was used to compare normally distributed continuous variables (age), and the Mann–Whitney U test to compare non-normally distributed continuous variables (NIHSS score, white cell count, C-reactive protein, and length of stay). Bold denotes statistical significance

*SD* standard deviation, *IQR* interquartile range, *ICH* intracerebral hemorrhage, *mRS* modified Rankin Scale, *OCSP* Oxfordshire Community Stroke Project, *COPD* chronic obstructive pulmonary disease, *WCC* white cell count, *CRP* C-reactive protein

Poisson regression models with a robust variance estimator were used to determine risk ratios for the in-hospital outcomes: functional outcome, mortality, and prolonged length of stay. The use of the robust variance estimator allows the relaxation of the assumption that the outcome follows a Poisson distribution and therefore allows the derivation of risk ratios with appropriate standard errors for an outcome following a binomial distribution [14, 15]. This method was chosen as an alternative to the traditional logistic regressions to yield risk ratios that are directly comparable to the hazard ratios for long-term mortality yielded by the Cox regressions [14]. Additional analyses of the in-hospital outcomes employing logistic regressions were performed to yield odds ratios directly comparable to previous and future literature employing logistic regressions (Table S3). Cox regressions were performed for 90-day, and 1-year mortality. Satisfaction of the proportional hazards assumption was confirmed in all models by visual inspection of log-negative-log survival curves. Risk ratios (RR), hazard ratios (HR), and odds ratios (OR) are reported alongside corresponding 95% confidence intervals (CI).

Three sequentially adjusted regression models were constructed for each model described above: a primary model that adjusted for all covariates and comorbidities except SAP and NIHSS score, an SAP-adjusted model, and an NIHSS score-adjusted model (also adjusted for SAP). Dedicated sensitivity analyses excluding all cases of SAP were also performed. A Kaplan–Meier survival analysis was performed to compare death between groups using a log-rank test.

Finally, a further Cox model was constructed to evaluate the relationship between WCC and CRP as continuous variables and the selected adverse outcomes. This adjusted for all the confounders listed above including SAP and NIHSS. The Akaike Information Criterion (AIC) was calculated for the linear model and for nonlinear models parametrized using restricted cubic spline (RCS) with varying degrees of freedom (*df* = 2 to *df* = 7) to determine the best fitting model delineating the association between WCC/CRP and long-term mortality. RCS were constructed using the Stata command *rcsgen* [16]. Where an RCS model had a lower AIC than the linear model, the likelihood-ratio test was used to confirm that this RCS model provides a better fit for the data than the linear model. WCC was therefore parametrized using RCS with 2 degrees of freedom (1 internal knot), while CRP was parametrized using a linear model. The reference point (HR = 1) for the WCC HR function was chosen as the minimum value of this function, corresponding to a WCC value of 7.0 × 10^9^/L in the main analyses and 5.8 × 10^9^/L in the sensitivity analyses excluding SAP patients. The satisfaction of the proportional hazards assumption for these models was verified using Schoenfeld residuals. The resulting hazard ratios characterizing the association between WCC/CRP and long-term mortality were therefore assumed to remain constant throughout the follow-up period.

As the interval from admission to measurement of inflammatory biomarkers varied, we performed sensitivity analyses replicating the main analyses, but excluding those patients whose biomarkers were sampled > 48 h after admission. These did not show any meaningful differences in results (Supplementary Tables S6 and S7).

## Results

### Cohort characteristics

A total of 1714 patients with ICH were identified following exclusions (Fig. 1). Table 1 displays the cohort characteristics and outcomes of included cases, performed on one of the imputed datasets resulting from the MICE algorithm. Descriptive statistics of the other 19 imputed datasets are presented in the Supplementary Tables (Tables S2.1–S2.20). The mean age (± SD) of patients was 76.05 (± 12.25); 49.82% of patients were male. The distribution of OCSP classifications varied between groups, with a higher proportion of TACS in the inflammatory biomarker group (*p* < 0.001). There was a lower proportion of male patients in the elevated inflammatory biomarkers cohort than in the control cohort (44.92% vs 51.69.7%, *P* = 0.012). Patients with elevated inflammatory biomarkers were more likely to have coronary heart disease (22.88% vs 17.63%, *P* = 0.013), and congestive heart failure (10.38% vs 6.92%, *P* = 0.018). Patients in the elevated inflammatory biomarkers cohort were more likely to be diagnosed with stroke associated pneumonia during admission than controls (15.89% vs 9.58%, *P* < 0.001).

Of the included patients, 472 (27.54%) had elevated inflammatory biomarkers at admission. The median WCC and CRP in the non-inflammatory group were 8.70 × 10^9^/L and 7.00 mg/L respectively; in the inflammatory cohort, they were 12.95 × 10^9^/L and 27.00 mg/L, respectively.

### Functional outcome

**Fig. 2:**
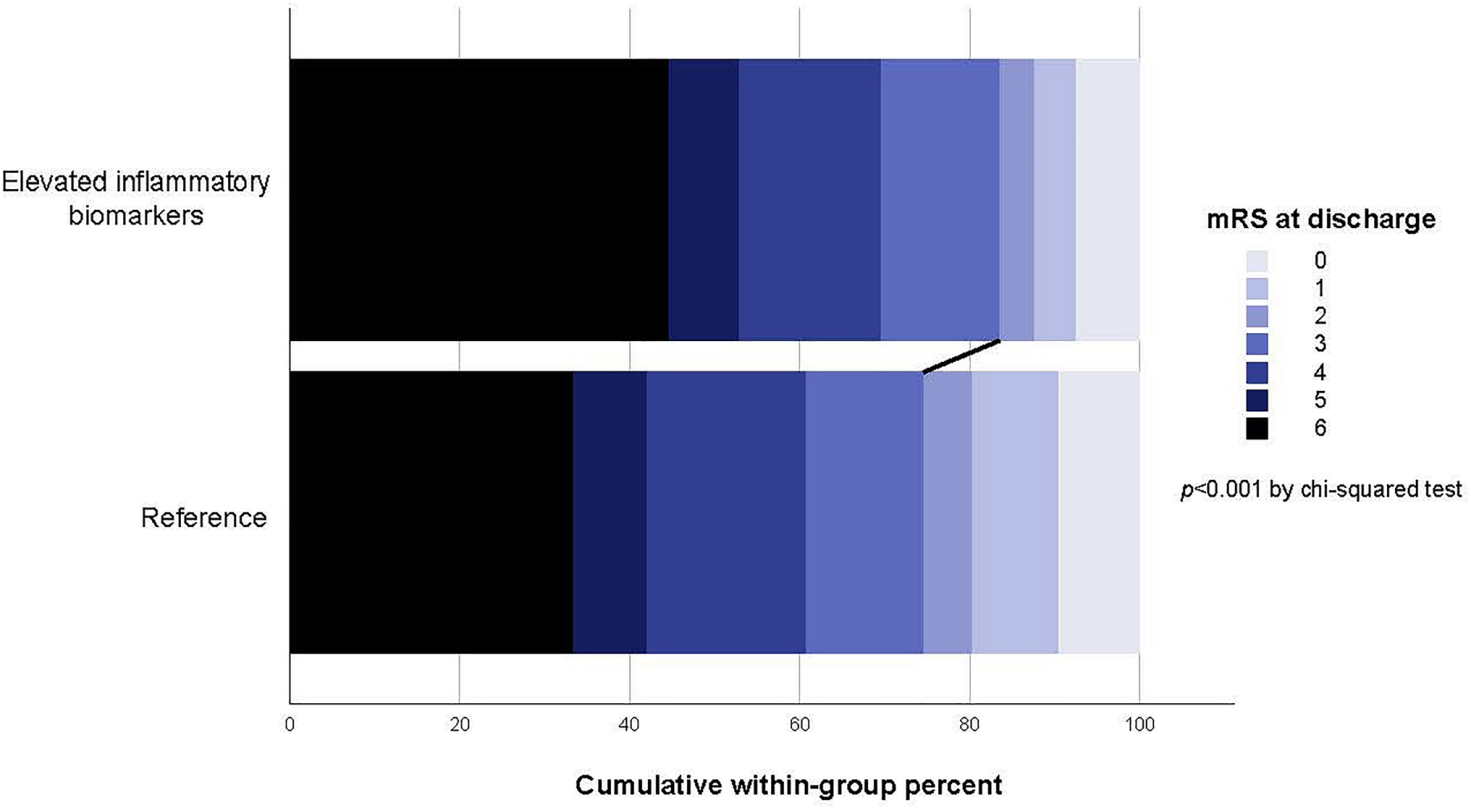
Stacked bar graph of functional outcome of patients with ICH. Patients with elevated inflammatory biomarkers at admission were more likely to experience poor functional outcome (mRS 3–6 at discharge; 83.5% vs 74.5% respectively; *p* < 0.001 by Chi-square test)

After adjusting for confounders, modified Poisson regression revealed that elevated inflammatory biomarkers at admission were associated with poor functional outcome (RR 1.09 [95% CI 1.02–1.16]) (Table 2, Fig. 2). This association was observed in the SAP-adjusted regression model (RR 1.08 [95% CI 1.01–1.15]) and in the NIHSS-adjusted regression model (RR 1.08 [95%CI 1.01–1.15]). In the sensitivity analyses excluding all cases of stroke associated pneumonia, as well as those excluding all patients whose biomarkers were sampled > 48 h after admission, elevated inflammatory biomarkers continued to be associated with poor functional outcomes (Tables S4 and S6).

**Table 2:** Results of multivariable Poisson regression models with a robust variance estimator^a^ assessing the relationship between co-elevation of WCC (> 10 × 10^9^/L) and CRP (> 10 mg/L) and in-hospital outcomes

Outcome	Primary model	SAP-adjusted model	NIHSS-adjusted model
	Risk ratios (95% confidence intervals)		
Poor functional outcome^b^	1.09 (1.02–1.16), *P* = 0.009	1.08 (1.01–1.15), *P* = 0.017	1.08 (1.01–1.15), *P* = 0.018
Death during admission	**1.22 (1.07–1.40), *P* = 0.002**	**1.21 (1.06–1.38), *P* = 0.004**	**1.21 (1.06–1.39), *P* = 0.004**
Length-of-stay > 14 days	1.14 (0.99–1.32), *P* = 0.067	1.11 (0.96–1.28), *P* = 0.159	1.11 (0.96–1.28), *P* = 0.163

*Primary model* adjusted for age, sex, pre-morbid modified Ranking Score, Oxfordshire Community Stroke Project classification, prevalent comorbidities on admission, and anticoagulant and antithrombotic medications on admission

*SAP-adjusted model* primary model + stroke-associated pneumonia, defined as any case of pneumonia within 7 days of intracerebral hemorrhage onset, as defined by the Pneumonia in Stroke Consensus Group^8^

*NIHSS-adjusted model* SAP-adjusted model + imputed values of the National Institute of Health Stroke Scale

*SAP* stroke-associated pneumonia, *NIHSS* National Institute of Health Stroke Scale. Bold denotes statistical significance

^a^The use of the robust variance estimator allows the relaxation of the assumption that the outcome follows a Poisson distribution and therefore allows the derivation of risk ratios with appropriate standard errors for an outcome following a binomial distribution^12,13^. This method was chosen as an alternative to the traditional logistic regressions to yield risk ratios that are directly comparable to the hazard ratios for long-term mortality yielded by the Cox regressions^12^. Additional analyses of the in-hospital outcomes employing logistic regressions were also performed in order to yield odds ratios directly comparable to previous and future literature employing logistic regressions (Table S3)

^b^Defined as modified Ranking Scale at discharge 3–6

### Mortality

**Fig. 3:**
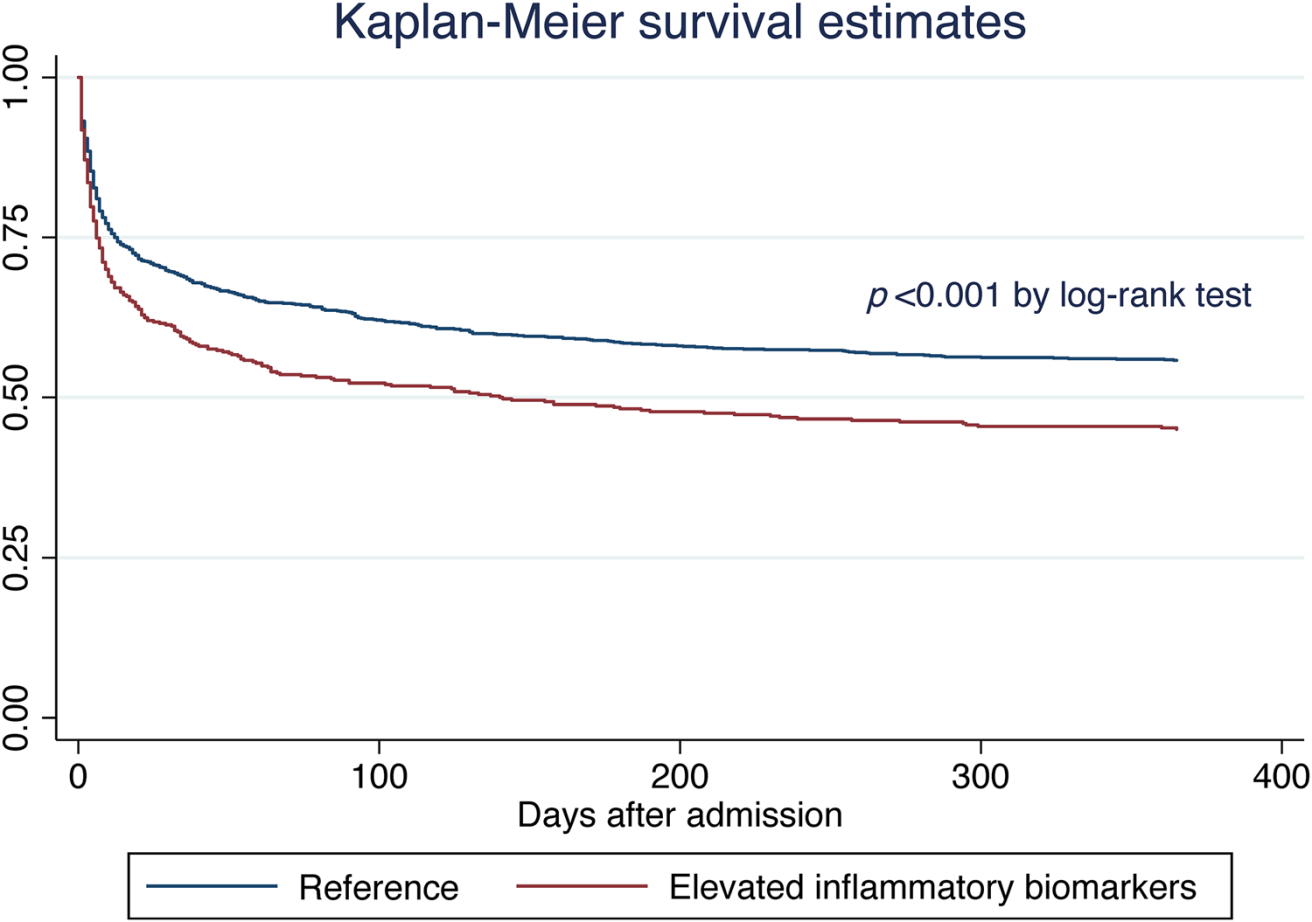
Kaplan–Meier survival analysis of patients with ICH. Patients with elevated inflammatory biomarkers at admission were more likely to be dead at 1-year follow-up (*p* < 0.001 by log-rank test)

After adjusting for all confounders including SAP and NIHSS, elevated inflammatory biomarkers were associated with death during admission (RR 1.21 [95% CI 1.06–1,39]), death at 90 days (HR 1.22 [95% CI 1.03–1.45]), and death at 1 year (HR 1.20 [95% CI 1.02–1.41]) (Tables 2, 3). In a sensitivity analysis excluding all SAP cases, elevated inflammatory biomarkers continued to be associated with death during admission (Table S4). In contrast, sensitivity analyses excluding all SAP cases did not show an association between co-elevation of inflammatory biomarkers on admission and post-discharge mortality at 90 days or 365 days (Table S5). Kaplan–Meier survival analysis revealed that patients with elevated inflammatory biomarkers died earlier and more frequently than their counterparts (*P* < 0.001 by log rank test; Fig. 3).

**Table 3:** Results of multivariable Cox models assessing the relationship between co-elevation of WCC (> 10 × 10^9^/L) and CRP (> 10 mg/L) and mortality at pre-specified timepoints after hospital discharge

Outcome	Primary model	SAP-adjusted model	NIHSS-adjusted model
	Hazard ratio (95% confidence interval)		
Day 90 mortality	**1.23 (1.03–1.47), *P* = 0.020**	**1.22 (1.03–1.45), *P* = 0.025**	**1.22 (1.03–1.45), *P* = 0.025**
Day 365 mortality	**1.22 (1.03–1.43), *P* = 0.018**	**1.20 (1.02–1.41), *P* = 0.025**	**1.20 (1.02–1.41), *P* = 0.025**
Day 5239^a^ mortality	**1.24 (1.07–1.44), *P* = 0.005**	**1.23 (1.06–1.43), *P* = 0.006**	**1.23 (1.06–1.43), *P* = 0.006**

*Primary model* adjusted for age, sex, pre-morbid modified Ranking Score, Oxfordshire Community Stroke Project classification, prevalent comorbidities on admission, and anticoagulant and antithrombotic medications on admission

*SAP-adjusted model* primary model + stroke-associated pneumonia, defined as any case of pneumonia within 7 days of intracerebral hemorrhage onset, as defined by the Pneumonia in Stroke Consensus Group^8^

*NIHSS-adjusted model* SAP-adjusted model + imputed values of the National Institute of Health Stroke Scale

*SAP* stroke-associated pneumonia, *NIHSS* National Institute of Health Stroke Scale. Bold denotes statistical significance

^a^End of follow-up

**Fig. 4:**
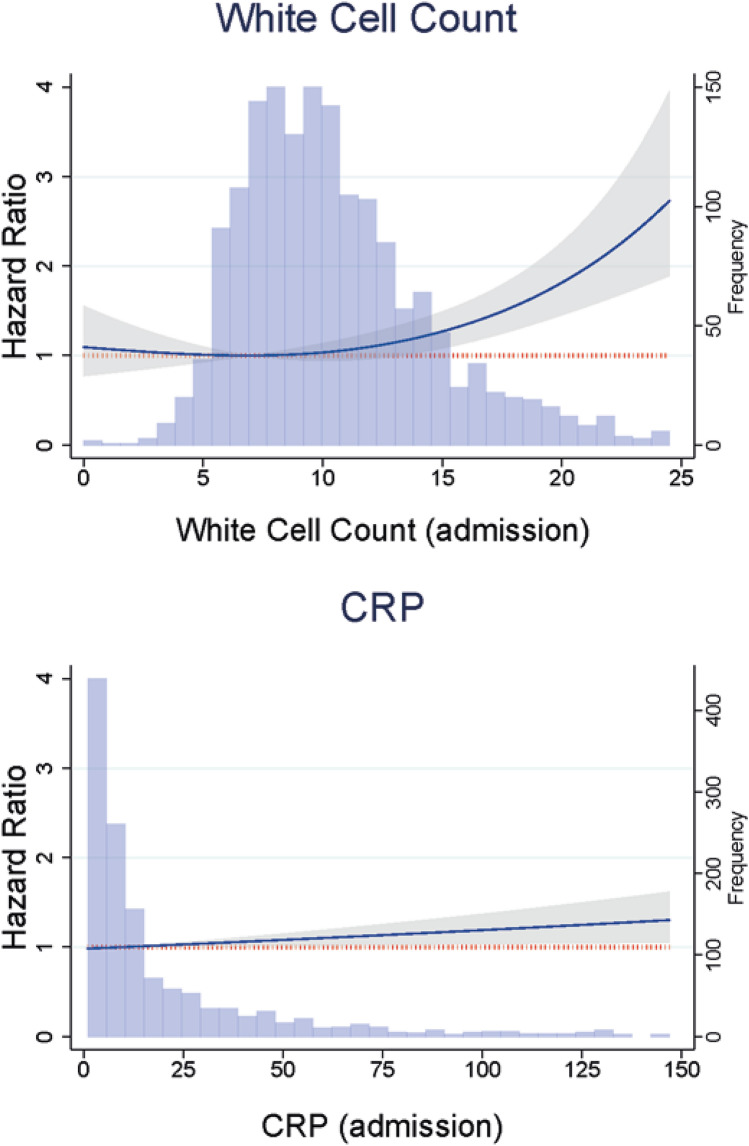
Results of multivariable Cox regressions assessing the association of white blood cell count (WCC) and C-reactive protein (CRP) as continuous variables, and long-term mortality after intracerebral hemorrhage. As the relationship between WCC and mortality did not follow a linear pattern, this was parametrized using restricted cubic splines with 1 internal knot. The reference point (HR = 1) for the HR function was chosen as the minimum value of the function, corresponding to a WCC value of 7.0 × 10^9^/L. The relationship between CRP and mortality followed a linear pattern and was modelled accordingly: HR (95% confidence interval) for 50-point increase in CRP = 1.10 (1.02–1.19). Hazard ratios and respective 95% confidence intervals are represented by the blue line with grey shadowing. The dotted red line represents the reference line (HR = 1). The overlaying blue bar chart displays the distribution of each inflammatory marker in the included cohort. Model adjusted for age, sex, Oxfordshire Community Stroke Project classification, pre-morbid modified Rankin Scale, prevalent comorbidities on admission, stroke-associated pneumonia, admission antiplatelet and anticoagulant medication, and the National Institute of Health Stroke Scale

Figure 4 shows the results of the Cox proportional hazards model detailing the association between WCC and CRP as continuous variables and long-term mortality. Relative to a WCC of a 7.0 × 10^9^/L, there was no association between WCC ranging between 0 and 13.2 × 10^9^/L and long-term mortality. Nevertheless, compared to a WCC of a 7.0 × 10^9^/L, WCC values ranging between 13.3 (HR 1.16 [95% CI 1.00–1.35]) and 25.0 × 10^9^/L (HR 2.90 [95% CI 1.95–4.31]) were associated with statistically significant excess risk of long-term mortality. Increasing CRP was significantly associated with a linear increase in excess long-term mortality (HR for 1-point increase 1.0019 [95% CI 1.0003–1.0035]; HR for 10-point increase 1.0194 [1.0034–1.0358]; HR for 50-point increase 1.1013 [1.017–1.1923]). In sensitivity analyses excluding all patients whose biomarkers were sampled > 48 h after admission, elevated inflammatory biomarkers continued to be associated with mortality at all timepoints assessed (Supplementary Tables S6 and S7).

### Length of stay

After adjusting for all confounders in the modified Poisson regression model, elevated inflammatory biomarkers were not associated with an increased length of stay (RR 1.11 [95% CI 0.96–1.28]). Sensitivity analyses excluding all SAP cases showed similar results (Table S4).

## Discussion

In this large cohort study using a prospectively maintained stroke register, co-elevation of WCC and CRP at admission predicted poor outcomes in ICH: patients were more likely to have a poor functional outcome, die during admission, be dead at 90 days, and be dead at 1 year. Critically, these associations were maintained after adjusting for stroke-associated pneumonia. This study therefore indicates that these readily available inflammatory biomarkers may be useful prognosticators in the context of ICH and underscore the importance of reactive inflammation in the pathophysiology of this disease.

A growing body of evidence reveals there is a reactive inflammatory process following ICH [3, 17], with experimental studies demonstrating that a wide variety of inflammatory processes occur, including infiltration of white cells [18], activation of microglia [19], and the release of inflammatory cytokines [6]. Notably, elevation of interleukin-6- the primary inducer of CRP [20]—has been found in animal and human studies [21]. These studies are consistent with the findings of this study: that there is considerable inflammation immediately following ICH. More specifically, given that experimental data demonstrate the local infiltration of leukocytes and activation of inflammatory cytokines in ICH, it is logical that such phenomena could be detected peripherally and be used for prognostication, as suggested by our results. This study further emphasizes the important role that inflammation plays in ICH.

Studies have previously reported that the extent of inflammation correlates with various measures of stroke magnitude [6, 22, 23], suggesting that inflammatory biomarkers may in turn correlate with clinical outcomes. This was the case in this study: patients with elevated inflammatory biomarkers had a higher risk of poor functional outcome and death at multiple timepoints. Accordingly, these inflammatory biomarkers may be useful for outcome prognostication. To date, however, the prognostic value of inflammatory biomarkers has been incompletely investigated, with small samples sizes and varying study thresholds limiting generalizability. A number of studies investigating a variety of white blood cell measurements in ICH have reported linear associations between increasing white cells and poor outcomes [7, 8], while three studies have reported that rising CRP is linearly associated with poor outcomes: these studies do not use discrete thresholds for denoting limiting the clinical applicability of these findings [24–26]. Notably, the present study cohort is larger than those of these previous analyses combined, providing greater statistical power to support these results. Notably, elevated inflammatory biomarkers were not associated with prolonged length of stay in this analysis after adjusting for covariates in regression models: it may be that the myriad factors that determine discharge are independent of the effects of post-ICH inflammation.

This study has several strengths. The protocol of the stroke register used for this study provides near-complete follow-up data capture and is prospectively maintained for research purposes, ensuring that data are systematically recorded. By adjusting for many covariates, including stroke-associated pneumonia, the risk of confounding is further reduced: incident pneumonia will not only cause an inflammatory marker increase that is independent of ICH-driven inflammation, but is also more likely to be associated with death and prolonged hospitalization. As biomarkers were sampled at varying timepoints after admission, this is particularly important. Additionally, this study uses clinically validated biomarker thresholds for determining study groups and considers both the combined and independent roles of WCC and CRP. Significantly, this study demonstrates that these inflammatory biomarkers are associated with poor outcomes independent of the effects of stroke-associated pneumonia, a key confounder unadjusted for in previous analyses [7, 8, 24–26].

This study is subject to a number of limitations. ICH volume and Graeb Score are not recorded in the register, and thus were not adjusted for in this analysis. Hematoma volume is a strong predictor of outcome in ICH and is likely associated with elevation of inflammatory biomarkers. We were, however, able to adjust for NIHSS and OCSP, providing an index of ICH severity. While there was a large proportion of missing data for NIHSS, we utilized MICE to impute missing values in order to reduce bias secondary to data missingness. Furthermore, it has been previously shown that this remains statistically robust even with a high proportion of data missingness when the data are likely missing-at-random, as was the case in our study [27]. Previous work has found that NIHSS score is correlated with ICH volume, suggesting that adjusting for NIHSS may account for much of the confounding effects of ICH volume [28]. While the OCSP classification is not a marker of stroke severity per se, there is a strong association between stroke severity and OCSP, with total anterior circulation stroke syndromes being likely more severe than partial anterior circulation stroke syndromes which are likely more severe than lacunar stroke syndromes. Having much lower proportion of missing OCSP than NIHSS data, we included this as an adjustment in all our analyses to reduce residual confounding from stroke severity. While we adjusted for the effect of stroke-associated pneumonia in multivariate regressions, we were not able to adjust for other infections such as urinary tract infections, and patients with pre-existing infections could not be excluded. Other infections may, in part, account for the worse outcomes experienced by patients with elevated inflammatory biomarkers in this cohort, and further work adjusting for a wider range of infections would be insightful. However, the large proportion of patients with elevated inflammatory biomarkers, and a meta-analysis of 137,817 patients reporting that post-stroke urinary tract infections are not associated with mortality [29], suggest that other infections would only partially account for these worse outcomes. As time from stroke onset to admission is not recorded in the register, we were unable to adjust for this in our analyses, and the interval between admission and biomarker measurement varied. We nevertheless performed a sensitivity analysis including only those patients whose biomarkers were measured within 48 h of admission in order to ascertain whether the temporal relationship between hospital admission and biomarker measurement may influence these associations. The results of this sensitivity analysis were not meaningfully different from the primary analyses, suggesting that, in this study, the exact timing of inflammatory marker quantification may not influence the association between inflammation and adverse ICH outcomes as long as they are measured during the acute phase. Nevertheless, it is important to highlight that most of our included cohort had undergone blood parameter measurement within 48 h of admission and therefore our sensitivity analyses may be underpowered to determine such temporal associations accurately. Further research into the role of inflammatory biomarkers across both the acute and post-acute periods is warranted.

The results of this study have several potential implications. In patients with ICH, inflammatory biomarkers at admission were associated with poor outcomes: this suggests that they may be useful for prognostication in the context of ICH. These biomarkers have been previously used for prognostication in a variety of other contexts, including ischemic stroke, myocardial infarction, and intensive care unit mortality [30–34]. Incorporating these inflammatory biomarkers into existing severity scores may further improve their ability to stratify ICH severity. Additionally, the findings of this study emphasize the importance of conducting a prospective cohort study investigating inflammatory biomarkers in ICH to investigate these hypotheses further. This study also underscores the importance of inflammation in the context of ICH, and as a potential target for the development of novel treatments. Such future treatments may require an index of inflammation for determining patient eligibility, for which WCC and CRP may be promising candidates.

## Conclusions

Co-elevation of WCC and CRP at admission was associated with an increased risk of poor functional outcome and death following ICH. These results underscore the importance of inflammation in ICH and suggest that these biomarkers may be valuable for prognostication in the context of ICH.

## Supplementary Information

Below is the link to the electronic supplementary material.

Supplementary file1 (DOCX 292 KB)

## Abbreviations

CI: Confidence interval
COPD: Chronic obstructive pulmonary disorder
CRP: C-reactive protein
HR: Hazard ratio
ICH: Intracerebral hemorrhage
LACS: Lacunar stroke syndrome
MICE: Multiple imputation by chained equation
mRS: Modified Rankin Scale
NIHSS: National Institute of Health Stroke Scale
OCSP: Oxford Community Stroke Project
OR: Odds ratio
PACS: Partial anterior circulation stroke syndrome
POCS: Posterior circulation stroke syndrome
RCS: Restricted cubic spline
RR: Risk ratio
SAH: Subarachnoid hemorrhage
SAP: Stroke-associated pneumonia
SD: Standard deviation
SIRS: Systemic inflammatory response syndrome
TACS: Total anterior circulation stroke syndrome
UTI: Urinary tract infection
WCC: White cell count
