# Supplementary material for: Elevated inflammatory biomarkers and poor outcomes in intracerebral hemorrhage
Source: J Neurol. 2022 Jul 22;269(12):6330–41. doi: 10.1007/s00415-022-11284-8 (PMC9618534; doi:10.1007/s00415-022-11284-8)
Supplement: Supplementary file 1 — Supplementary file1 (DOCX 292 KB) [file 415_2022_11284_MOESM1_ESM.docx]

Supplementary Tables S1.1 – S1.5: Missing data analysis tables.

Table S1.1: Missing data analysis for mRS at discharge.

|  | ICH admissions stratified by discharge mRS missingness | | |  |
| --- | --- | --- | --- | --- |
| Variable | Total cohort (*n*= 1714) | Non-missing (n= 1355) | Missing (n= 359) | *P-*value |
| Age- mean (+/- SD) | 76.05 (12.25) | 76.50 (12.23) | 74.37 (12.18) | **0.003** |
| Sex- no. male (%) | 854 (49.82) | 678 (50.04) | 176 (49.03) | 0.733 |
| Comorbidities |  |  |  |  |
| Asthma- no. (%) | 146 (8.52) | 111 (8.19) | 35 (9.75) | 0.347 |
| Atrial fibrillation- no. (%) | 383 (22.35) | 316 (23.32) | 67 (18.66) | 0.060 |
| Cerebral infarction- no. (%) | 135 (7.88) | 113 (8.34) | 22 (6.13) | 0.167 |
| Coronary heart disease- no. (%) | 327 (19.08) | 268 (19.78) | 59 (16.43) | 0.152 |
| Congestive heart failure- no. (%) | 135 (7.88) | 105 (7.75) | 30 (8.36) | 0.704 |
| Chronic kidney disease- no. (%) | 85 (4.96) | 69 (5.09) | 16 (4.46) | 0.622 |
| COPD- no. (%) | 104 (6.07) | 85 (6.27) | 19 (5.29) | 0.489 |
| Dementia- no. (%) | 71 (4.14) | 61 (4.5) | 10 (2.79) | 0.147 |
| Diabetes mellitus- no. (%) | 205 (11.96) | 165 (12.18) | 40 (11.14) | 0.591 |
| Hyperlipidemia- no. (%) | 179 (10.44) | 165 (12.18) | 14 (3.9) | **<0.001** |
| Hypertension- no. (%) | 996 (58.11) | 819 (60.44) | 177 (49.3) | **<0.001** |
| Liver disease- no. (%) | 26 (1.52) | 21 (1.55) | 5 (1.39) | 0.829 |
| Malignancy- no. (%) | 243 (14.18) | 207 (15.28) | 36 (10.03) | **0.011** |
| Peptic ulcer disease- no. (%) | 64 (3.73) | 44 (3.25) | 20 (5.57) | **0.039** |
| Peripheral vascular disease- no. (%) | 82 (4.78) | 65 (4.8) | 17 (4.74) | 0.961 |
| Previous ICH/SAH- no. (%) | 377 (22) | 333 (24.58) | 44 (12.26) | **<0.001** |
| Connective tissue disease- no. (%) | 62 (3.62) | 54 (3.99) | 8 (2.23) | 0.113 |
| Stroke-associated pneumonia- no. (%) | 194 (11.32) | 170 (12.55) | 24 (6.69) | **0.002** |
| Admission medications |  |  |  |  |
| Anticoagulants- no. (%) | 34 (1.98) | 34 (2.51) | 0 (0) | **0.002** |
| Antiplatelets- no. (%) | 604 (35.24) | 472 (34.83) | 132 (36.77) | 0.495 |
| Biomarkers at admission |  |  |  |  |
| White cell count (x10^9/L) - median (IQR) | 9.90 (7.60-12.60) | 9.80 (7.60-12.90) | 10.10 (8.00-12.10) | 0.648 |
| Outcomes |  |  |  |  |
| Mortality |  |  |  |  |
| Death during admission- no. (%) | 623 (36.35) | 623 (45.98) | 0 (0) | **<0.001** |
| Death at 90 days- no. (%) | 714 (41.66) | 678 (50.04) | 36 (10.03) | **<0.001** |
| Death at 365 days- no. (%) | 834 (48.66) | 760 (56.09) | 74 (20.61) | **<0.001** |
| Length of stay |  |  |  |  |
| Days- median (IQR) | 8.00 (3.00-19.00) | 6.00 (2.00-16.00) | 17.00 (7.00-31.00) | **<0.001** |
| Length of stay >14 days- no. (%) | 576 (33.61) | 380 (28.04) | 196 (54.6) | **<0.001** |

Table S1.2: Missing data analysis for CRP at admission.

|  | ICH admissions stratified by admission CRP missingness | | |  |
| --- | --- | --- | --- | --- |
| Variable | Total cohort (*n*= 1714) | Non-missing (n= 1411) | Missing (n= 303) | *P-*value |
| Age- mean (+/- SD) | 76.05 (12.25) | 76.61 (11.75) | 73.47 (14.06) | **<0.001** |
| Sex- no. male (%) | 854 (49.82) | 698 (49.47) | 156 (51.49) | 0.524 |
| Comorbidities |  |  |  |  |
| Asthma- no. (%) | 146 (8.52) | 121 (8.58) | 25 (8.25) | 0.854 |
| Atrial fibrillation- no. (%) | 383 (22.35) | 345 (24.45) | 38 (12.54) | **<0.001** |
| Cerebral infarction- no. (%) | 135 (7.88) | 129 (9.14) | 6 (1.98) | **<0.001** |
| Coronary heart disease- no. (%) | 327 (19.08) | 287 (20.34) | 40 (13.2) | **0.004** |
| Congestive heart failure- no. (%) | 135 (7.88) | 117 (8.29) | 18 (5.94) | 0.168 |
| Chronic kidney disease- no. (%) | 85 (4.96) | 82 (5.81) | 3 (.99) | **<0.001** |
| COPD- no. (%) | 104 (6.07) | 96 (6.8) | 8 (2.64) | **0.006** |
| Dementia- no. (%) | 71 (4.14) | 65 (4.61) | 6 (1.98) | **0.037** |
| Diabetes mellitus- no. (%) | 205 (11.96) | 183 (12.97) | 22 (7.26) | **0.005** |
| Hyperlipidemia- no. (%) | 179 (10.44) | 163 (11.55) | 16 (5.28) | **0.001** |
| Hypertension- no. (%) | 996 (58.11) | 854 (60.52) | 142 (46.86) | **<0.001** |
| Liver disease- no. (%) | 26 (1.52) | 23 (1.63) | 3 (.99) | 0.408 |
| Malignancy- no. (%) | 243 (14.18) | 213 (15.1) | 30 (9.9) | **0.019** |
| Peptic ulcer disease- no. (%) | 64 (3.73) | 56 (3.97) | 8 (2.64) | 0.268 |
| Peripheral vascular disease- no. (%) | 82 (4.78) | 76 (5.39) | 6 (1.98) | **0.012** |
| Previous ICH/SAH- no. (%) | 377 (22) | 337 (23.88) | 40 (13.2) | **<0.001** |
| Connective tissue disease- no. (%) | 62 (3.62) | 57 (4.04) | 5 (1.65) | **0.043** |
| Stroke-associated pneumonia- no. (%) | 194 (11.32) | 179 (12.69) | 15 (4.95) | **<0.001** |
| Admission medications |  |  |  |  |
| Anticoagulants- no. (%) | 34 (1.98) | 32 (2.27) | 2 (.66) | 0.069 |
| Antiplatelets- no. (%) | 604 (35.24) | 502 (35.58) | 102 (33.66) | 0.527 |
| Biomarkers at admission |  |  |  |  |
| White cell count (x10^9/L) - median (IQR) | 9.90 (7.60-12.60) | 9.90 (7.70-12.70) | 9.30 (7.30-12.30) | **0.034** |
| Outcomes |  |  |  |  |
| Mortality |  |  |  |  |
| Death during admission- no. (%) | 623 (36.35) | 471 (33.38) | 152 (50.17) | **<0.001** |
| Death at 90 days- no. (%) | 714 (41.66) | 544 (38.55) | 170 (56.11) | **<0.001** |
| Death at 365 days- no. (%) | 834 (48.66) | 658 (46.63) | 176 (58.09) | **<0.001** |
| Length of stay |  |  |  |  |
| Days- median (IQR) | 8.00 (3.00-19.00) | 10.00 (4.00-23.00) | 3.00 (1.00-7.00) | **<0.001** |
| Length of stay >14 days- no. (%) | 576 (33.61) | 549 (38.91) | 27 (8.91) | **<0.001** |

Table S1.3: Missing data analysis for OCSP classification.

|  | ICH admissions stratified by OCSP classification missingness | | |  |
| --- | --- | --- | --- | --- |
| Variable | Total cohort (*n*= 1714) | Non-missing (n= 1345) | Missing (n= 369) | *P-*value |
| Age- mean (+/- SD) | 76.05 (12.25) | 76.59 (11.75) | 74.10 (13.74) | **<0.001** |
| Sex- no. male (%) | 854 (49.82) | 669 (49.74) | 185 (50.14) | 0.893 |
| Comorbidities |  |  |  |  |
| Asthma- no. (%) | 146 (8.52) | 116 (8.62) | 30 (8.13) | 0.763 |
| Atrial fibrillation- no. (%) | 383 (22.35) | 302 (22.45) | 81 (21.95) | 0.837 |
| Cerebral infarction- no. (%) | 135 (7.88) | 99 (7.36) | 36 (9.76) | 0.130 |
| Coronary heart disease- no. (%) | 327 (19.08) | 255 (18.96) | 72 (19.51) | 0.811 |
| Congestive heart failure- no. (%) | 135 (7.88) | 108 (8.03) | 27 (7.32) | 0.653 |
| Chronic kidney disease- no. (%) | 85 (4.96) | 68 (5.06) | 17 (4.61) | 0.725 |
| COPD- no. (%) | 104 (6.07) | 87 (6.47) | 17 (4.61) | 0.185 |
| Dementia- no. (%) | 71 (4.14) | 50 (3.72) | 21 (5.69) | 0.092 |
| Diabetes mellitus- no. (%) | 205 (11.96) | 172 (12.79) | 33 (8.94) | **0.044** |
| Hyperlipidemia- no. (%) | 179 (10.44) | 145 (10.78) | 34 (9.21) | 0.383 |
| Hypertension- no. (%) | 996 (58.11) | 786 (58.44) | 210 (56.91) | 0.598 |
| Liver disease- no. (%) | 26 (1.52) | 20 (1.49) | 6 (1.63) | 0.847 |
| Malignancy- no. (%) | 243 (14.18) | 197 (14.65) | 46 (12.47) | 0.287 |
| Peptic ulcer disease- no. (%) | 64 (3.73) | 51 (3.79) | 13 (3.52) | 0.809 |
| Peripheral vascular disease- no. (%) | 82 (4.78) | 64 (4.76) | 18 (4.88) | 0.924 |
| Previous ICH/SAH - no. (%) | 377 (22) | 308 (22.9) | 69 (18.7) | 0.084 |
| Connective tissue disease- no. (%) | 62 (3.62) | 52 (3.87) | 10 (2.71) | 0.292 |
| Stroke-associated pneumonia- no. (%) | 194 (11.32) | 170 (12.64) | 24 (6.5) | **<0.001** |
| Admission medications |  |  |  |  |
| Anticoagulants- no. (%) | 34 (1.98) | 28 (2.08) | 6 (1.63) | 0.578 |
| Antiplatelets- no. (%) | 604 (35.24) | 450 (33.46) | 154 (41.73) | **0.003** |
| Biomarkers at admission |  |  |  |  |
| White cell count (x10^9/L) - median (IQR) | 9.90 (7.60-12.60) | 9.60 (7.50-12.30) | 10.80 (8.30-14.00) | **<0.001** |
| Outcomes |  |  |  |  |
| Mortality |  |  |  |  |
| Death during admission- no. (%) | 623 (36.35) | 463 (34.42) | 160 (43.36) | **0.002** |
| Death at 90 days- no. (%) | 714 (41.66) | 534 (39.7) | 180 (48.78) | **0.002** |
| Death at 365 days- no. (%) | 834 (48.66) | 638 (47.43) | 196 (53.12) | 0.053 |
| Length of stay |  |  |  |  |
| Days- median (IQR) | 8.00 (3.00-19.00) | 9.00 (4.00-20.00) | 4.00 (1.00-13.00) | **<0.001** |
| Length of stay >14 days- no. (%) | 576 (33.61) | 487 (36.21) | 89 (24.12) | **<0.001** |

Table S1.4: Missing data analysis for pre-ICH mRS.

|  | ICH admissions | | |  |
| --- | --- | --- | --- | --- |
| Variable | Total cohort (*n*= 1714) | Non-missing (n= 1589) | Missing (n= 125) | *P-*value |
| Age- mean (+/- SD) | 76.05 (12.25) | 76.07 (12.28) | 75.85 (11.87) | 0.849 |
| Sex- no. male (%) | 854 (49.82) | 800 (50.35) | 54 (43.2) | 0.124 |
| Comorbidities |  |  |  |  |
| Asthma- no. (%) | 146 (8.52) | 134 (8.43) | 12 (9.6) | 0.653 |
| Atrial fibrillation- no. (%) | 383 (22.35) | 350 (22.03) | 33 (26.4) | 0.258 |
| Cerebral infarction- no. (%) | 135 (7.88) | 123 (7.74) | 12 (9.6) | 0.457 |
| Coronary heart disease- no. (%) | 327 (19.08) | 303 (19.07) | 24 (19.2) | 0.971 |
| Congestive heart failure- no. (%) | 135 (7.88) | 126 (7.93) | 9 (7.2) | 0.771 |
| Chronic kidney disease- no. (%) | 85 (4.96) | 79 (4.97) | 6 (4.8) | 0.932 |
| COPD- no. (%) | 104 (6.07) | 98 (6.17) | 6 (4.8) | 0.538 |
| Dementia- no. (%) | 71 (4.14) | 68 (4.28) | 3 (2.4) | 0.310 |
| Diabetes mellitus- no. (%) | 205 (11.96) | 192 (12.08) | 13 (10.4) | 0.577 |
| Hyperlipidemia- no. (%) | 179 (10.44) | 175 (11.01) | 4 (3.2) | **0.006** |
| Hypertension- no. (%) | 996 (58.11) | 934 (58.78) | 62 (49.6) | **0.045** |
| Liver disease- no. (%) | 26 (1.52) | 24 (1.51) | 2 (1.6) | 0.937 |
| Malignancy- no. (%) | 243 (14.18) | 219 (13.78) | 24 (19.2) | 0.095 |
| Peptic ulcer disease- no. (%) | 64 (3.73) | 56 (3.52) | 8 (6.4) | 0.103 |
| Peripheral vascular disease- no. (%) | 82 (4.78) | 75 (4.72) | 7 (5.6) | 0.657 |
| Previous ICH/SAH- no. (%) | 377 (22) | 357 (22.47) | 20 (16) | 0.093 |
| Connective tissue disease- no. (%) | 62 (3.62) | 59 (3.71) | 3 (2.4) | 0.449 |
| Stroke-associated pneumonia- no. (%) | 194 (11.32) | 176 (11.08) | 18 (14.4) | 0.259 |
| Admission medications |  |  |  |  |
| Anticoagulants- no. (%) | 34 (1.98) | 34 (2.14) | 0 (0) | 0.099 |
| Antiplatelets- no. (%) | 604 (35.24) | 573 (36.06) | 31 (24.8) | **0.011** |
| Biomarkers at admission |  |  |  |  |
| White cell count (x10^9/L) - median (IQR) | 9.90 (7.60-12.60) | 9.80 (7.60-12.40) | 11.20 (8.80-14.60) | **<0.001** |
| Outcomes |  |  |  |  |
| Mortality |  |  |  |  |
| Death during admission- no. (%) | 623 (36.35) | 566 (35.62) | 57 (45.6) | **0.026** |
| Death at 30 days- no. (%) | 597 (34.83) | 540 (33.98) | 57 (45.6) | **0.009** |
| Death at 90 days- no. (%) | 714 (41.66) | 645 (40.59) | 69 (55.2) | **0.001** |
| Death at 365 days- no. (%) | 834 (48.66) | 753 (47.39) | 81 (64.8) | **<0.001** |
| Length of stay |  |  |  |  |
| Days- median (IQR) | 8.00 (3.00-19.00) | 8.00 (3.00-19.00) | 9.00 (1.00-23.00) | 0.945 |
| Length of stay >14 days- no. (%) | 576 (33.61) | 528 (33.23) | 48 (38.4) | 0.239 |

Table S1.5: Missing data analysis for NIHSS.

|  | ICH admissions by NIHSS missingness | | |  |
| --- | --- | --- | --- | --- |
| Variable | Total cohort (*n*= 1714) | Non-missing (n= 234) | Missing (n= 1480) | *P-*value |
| Age- mean (+/- SD) | 76.05 (12.25) | 75.82 (13.04) | 76.09 (12.12) | 0.759 |
| Sex- no. male (%) | 854 (49.82) | 138 (58.97) | 716 (48.38) | **0.003** |
| Comorbidities |  |  |  |  |
| Asthma- no. (%) | 146 (8.52) | 16 (6.84) | 130 (8.78) | 0.322 |
| Atrial fibrillation- no. (%) | 383 (22.35) | 45 (19.23) | 338 (22.84) | 0.218 |
| Cerebral infarction- no. (%) | 135 (7.88) | 22 (9.4) | 113 (7.64) | 0.351 |
| Coronary heart disease- no. (%) | 327 (19.08) | 40 (17.09) | 287 (19.39) | 0.406 |
| Congestive heart failure- no. (%) | 135 (7.88) | 14 (5.98) | 121 (8.18) | 0.247 |
| Chronic kidney disease- no. (%) | 85 (4.96) | 20 (8.55) | 65 (4.39) | **0.007** |
| COPD- no. (%) | 104 (6.07) | 16 (6.84) | 88 (5.95) | 0.595 |
| Dementia- no. (%) | 71 (4.14) | 15 (6.41) | 56 (3.78) | 0.061 |
| Diabetes mellitus- no. (%) | 205 (11.96) | 36 (15.38) | 169 (11.42) | 0.082 |
| Hyperlipidemia- no. (%) | 179 (10.44) | 42 (17.95) | 137 (9.26) | **<0.001** |
| Hypertension- no. (%) | 996 (58.11) | 150 (64.1) | 846 (57.16) | **0.046** |
| Liver disease- no. (%) | 26 (1.52) | 6 (2.56) | 20 (1.35) | 0.158 |
| Malignancy- no. (%) | 243 (14.18) | 47 (20.09) | 196 (13.24) | **0.005** |
| Peptic ulcer disease- no. (%) | 64 (3.73) | 16 (6.84) | 48 (3.24) | **0.007** |
| Peripheral vascular disease- no. (%) | 82 (4.78) | 8 (3.42) | 74 (5) | 0.292 |
| Previous ICH/SAH- no. (%) | 377 (22) | 196 (83.76) | 181 (12.23) | **<0.001** |
| Connective tissue disease- no. (%) | 62 (3.62) | 8 (3.42) | 54 (3.65) | 0.861 |
| Stroke-associated pneumonia- no. (%) | 194 (11.32) | 13 (5.56) | 181 (12.23) | **0.003** |
| Admission medications |  |  |  |  |
| Anticoagulants- no. (%) | 34 (1.98) | 34 (14.53) | 0 (0) | **<0.001** |
| Antiplatelets- no. (%) | 604 (35.24) | 1 (.43) | 603 (40.74) | **<0.001** |
| Biomarkers at admission |  |  |  |  |
| White cell count (x10^9/L) - median (IQR) | 9.90 (7.60-12.60) | 9.00 (7.50-11.30) | 10.00 (7.70-12.75) | **<0.001** |
| Outcomes |  |  |  |  |
| Mortality |  |  |  |  |
| Death during admission- no. (%) | 623 (36.35) | 64 (27.35) | 559 (37.77) | **0.002** |
| Death at 90 days- no. (%) | 714 (41.66) | 74 (31.62) | 640 (43.24) | **<0.001** |
| Death at 365 days- no. (%) | 834 (48.66) | 89 (38.03) | 745 (50.34) | **<0.001** |
| Length of stay |  |  |  |  |
| Days- median (IQR) | 8.00 (3.00-19.00) | 9.00 (3.00-23.00) | 8.00 (3.00-19.00) | 0.075 |
| Length of stay >14 days- no. (%) | 576 (33.61) | 87 (37.18) | 489 (33.04) | 0.213 |

Supplementary Tables S2.1 – S2.20: Descriptive statistics for all 20 imputed datasets.

Table S2.1

|  | ICH admissions | | |  |
| --- | --- | --- | --- | --- |
| Variable | Total cohort (*n*= 1714) | Non-elevated inflammatory biomarkers (n= 1242) | Elevated inflammatory biomarkers (n= 472) | *P-*value |
| Age- mean (+/- SD) | 76.05 (12.25) | 76.01 (12.32) | 76.16 (12.07) | 0.829 |
| Sex- no. male (%) | 854 (49.82) | 642 (51.69) | 212 (44.92) | **0.012** |
| Total NIHSS score- median (IQR) | 5.00 (2.00-10.00) | 5.00 (2.00-10.00) | 5.00 (2.00-10.00) | 0.527 |
| Pre-ICH mRS |  |  |  |  |
| 0- no. (%) | 1135 (66.22) | 837 (67.39) | 298 (63.14) | **0.005** |
| 1- no. (%) | 188 (10.97) | 137 (11.03) | 51 (10.81) | **0.005** |
| 2- no. (%) | 129 (7.53) | 103 (8.29) | 26 (5.51) | **0.005** |
| 3- no. (%) | 168 (9.8) | 108 (8.7) | 60 (12.71) | **0.005** |
| 4- no. (%) | 69 (4.03) | 41 (3.3) | 28 (5.93) | **0.005** |
| 5- no. (%) | 25 (1.46) | 16 (1.29) | 9 (1.91) | **0.005** |
| OCSP classification |  |  |  |  |
| TACS | 561 (32.73) | 371 (29.87) | 190 (40.25) | **<0.001** |
| PACS | 494 (28.82) | 365 (29.39) | 129 (27.33) | **<0.001** |
| LACS | 138 (8.05) | 104 (8.37) | 34 (7.2) | **<0.001** |
| POCS | 521 (30.4) | 402 (32.37) | 119 (25.21) | **<0.001** |
| Comorbidities |  |  |  |  |
| Asthma- no. (%) | 146 (8.52) | 114 (9.18) | 32 (6.78) | 0.112 |
| Atrial fibrillation- no. (%) | 383 (22.35) | 268 (21.58) | 115 (24.36) | 0.216 |
| Cerebral infarction- no. (%) | 135 (7.88) | 99 (7.97) | 36 (7.63) | 0.813 |
| Coronary heart disease- no. (%) | 327 (19.08) | 219 (17.63) | 108 (22.88) | **0.013** |
| Congestive heart failure- no. (%) | 135 (7.88) | 86 (6.92) | 49 (10.38) | **0.018** |
| Chronic kidney disease- no. (%) | 85 (4.96) | 57 (4.59) | 28 (5.93) | 0.253 |
| COPD- no. (%) | 104 (6.07) | 80 (6.44) | 24 (5.08) | 0.293 |
| Dementia- no. (%) | 71 (4.14) | 51 (4.11) | 20 (4.24) | 0.903 |
| Diabetes mellitus- no. (%) | 205 (11.96) | 148 (11.92) | 57 (12.08) | 0.927 |
| Hyperlipidemia- no. (%) | 179 (10.44) | 135 (10.87) | 44 (9.32) | 0.349 |
| Hypertension- no. (%) | 996 (58.11) | 730 (58.78) | 266 (56.36) | 0.364 |
| Liver disease- no. (%) | 26 (1.52) | 23 (1.85) | 3 (.64) | 0.066 |
| Malignancy- no. (%) | 243 (14.18) | 178 (14.33) | 65 (13.77) | 0.766 |
| Peptic ulcer disease- no. (%) | 64 (3.73) | 43 (3.46) | 21 (4.45) | 0.336 |
| Peripheral vascular disease- no. (%) | 82 (4.78) | 60 (4.83) | 22 (4.66) | 0.883 |
| Previous ICH/SAH- no. (%) | 377 (22) | 282 (22.71) | 95 (20.13) | 0.250 |
| Connective tissue disease- no. (%) | 62 (3.62) | 47 (3.78) | 15 (3.18) | 0.548 |
| Stroke-associated pneumonia- no. (%) | 194 (11.32) | 119 (9.58) | 75 (15.89) | **<0.001** |
| Admission medications |  |  |  |  |
| Anticoagulants- no. (%) | 34 (1.98) | 29 (2.33) | 5 (1.06) | 0.091 |
| Antiplatelets- no. (%) | 604 (35.24) | 429 (34.54) | 175 (37.08) | 0.326 |
| Biomarkers at admission |  |  |  |  |
| White cell count (x10^9/L) - median (IQR) | 9.90 (7.60-12.60) | 8.70 (7.00-10.60) | 12.95 (11.30-16.25) | **<0.001** |
| CRP (mg/L)- median (IQR) | 10.00 (4.00-27.00) | 7.00 (4.00-14.00) | 27.00 (14.00-57.00) | **<0.001** |
| Outcomes |  |  |  |  |
| Poor functional outcome- no. (%) | 1319 (76.95) | 925 (74.48) | 394 (83.47) | **<0.001** |
| mRS at discharge |  |  |  |  |
| Discharge mRS 0- no. (%) | 156 (9.1) | 120 (9.66) | 36 (7.63) | **<0.001** |
| Discharge mRS 1- no. (%) | 149 (8.69) | 126 (10.14) | 23 (4.87) | **<0.001** |
| Discharge mRS 2- no. (%) | 90 (5.25) | 71 (5.72) | 19 (4.03) | **<0.001** |
| Discharge mRS 3- no. (%) | 238 (13.89) | 172 (13.85) | 66 (13.98) | **<0.001** |
| Discharge mRS 4- no. (%) | 312 (18.2) | 233 (18.76) | 79 (16.74) | **<0.001** |
| Discharge mRS 5- no. (%) | 146 (8.52) | 107 (8.62) | 39 (8.26) | **<0.001** |
| Discharge mRS 6- no. (%) | 623 (36.35) | 413 (33.25) | 210 (44.49) | **<0.001** |
| Mortality |  |  |  |  |
| Death during admission- no. (%) | 623 (36.35) | 413 (33.25) | 210 (44.49) | **<0.001** |
| Death at 90 days- no. (%) | 714 (41.66) | 480 (38.65) | 234 (49.58) | **<0.001** |
| Death at 365 days- no. (%) | 834 (48.66) | 568 (45.73) | 266 (56.36) | **<0.001** |
| Length of stay |  |  |  |  |
| Days- median (IQR) | 8.00 (3.00-19.00) | 8.00 (3.00-19.00) | 8.00 (3.00-23.00) | 0.068 |
| Length of stay >14 days- no. (%) | 576 (33.61) | 396 (31.88) | 180 (38.14) | **0.014** |

Table S2.2

|  | ICH admissions | | |  |
| --- | --- | --- | --- | --- |
| Variable | Total cohort (*n*= 1714) | Non-elevated inflammatory biomarkers (n= 1236) | Elevated inflammatory biomarkers (n= 478) | *P-*value |
| Age- mean (+/- SD) | 76.05 (12.25) | 75.99 (12.26) | 76.21 (12.22) | 0.743 |
| Sex- no. male (%) | 854 (49.82) | 638 (51.62) | 216 (45.19) | **0.017** |
| Total NIHSS score- median (IQR) | 4.00 (2.00-8.00) | 4.00 (2.00-8.00) | 4.00 (2.00-8.00) | 0.077 |
| Pre-ICH mRS |  |  |  |  |
| 0- no. (%) | 1143 (66.69) | 831 (67.23) | 312 (65.27) | **0.025** |
| 1- no. (%) | 194 (11.32) | 142 (11.49) | 52 (10.88) | **0.025** |
| 2- no. (%) | 125 (7.29) | 100 (8.09) | 25 (5.23) | **0.025** |
| 3- no. (%) | 162 (9.45) | 107 (8.66) | 55 (11.51) | **0.025** |
| 4- no. (%) | 63 (3.68) | 37 (2.99) | 26 (5.44) | **0.025** |
| 5- no. (%) | 27 (1.58) | 19 (1.54) | 8 (1.67) | **0.025** |
| OCSP classification |  |  |  |  |
| TACS | 563 (32.85) | 369 (29.85) | 194 (40.59) | **<0.001** |
| PACS | 492 (28.7) | 371 (30.02) | 121 (25.31) | **<0.001** |
| LACS | 139 (8.11) | 103 (8.33) | 36 (7.53) | **<0.001** |
| POCS | 520 (30.34) | 393 (31.8) | 127 (26.57) | **<0.001** |
| Comorbidities |  |  |  |  |
| Asthma- no. (%) | 146 (8.52) | 114 (9.22) | 32 (6.69) | 0.093 |
| Atrial fibrillation- no. (%) | 383 (22.35) | 263 (21.28) | 120 (25.1) | 0.088 |
| Cerebral infarction- no. (%) | 135 (7.88) | 99 (8.01) | 36 (7.53) | 0.742 |
| Coronary heart disease- no. (%) | 327 (19.08) | 223 (18.04) | 104 (21.76) | 0.079 |
| Congestive heart failure- no. (%) | 135 (7.88) | 87 (7.04) | 48 (10.04) | **0.038** |
| Chronic kidney disease- no. (%) | 85 (4.96) | 57 (4.61) | 28 (5.86) | 0.287 |
| COPD- no. (%) | 104 (6.07) | 80 (6.47) | 24 (5.02) | 0.259 |
| Dementia- no. (%) | 71 (4.14) | 50 (4.05) | 21 (4.39) | 0.746 |
| Diabetes mellitus- no. (%) | 205 (11.96) | 148 (11.97) | 57 (11.92) | 0.977 |
| Hyperlipidemia- no. (%) | 179 (10.44) | 133 (10.76) | 46 (9.62) | 0.490 |
| Hypertension- no. (%) | 996 (58.11) | 721 (58.33) | 275 (57.53) | 0.763 |
| Liver disease- no. (%) | 26 (1.52) | 24 (1.94) | 2 (.42) | **0.021** |
| Malignancy- no. (%) | 243 (14.18) | 177 (14.32) | 66 (13.81) | 0.785 |
| Peptic ulcer disease- no. (%) | 64 (3.73) | 44 (3.56) | 20 (4.18) | 0.541 |
| Peripheral vascular disease- no. (%) | 82 (4.78) | 60 (4.85) | 22 (4.6) | 0.827 |
| Previous ICH/SAH- no. (%) | 377 (22) | 279 (22.57) | 98 (20.5) | 0.353 |
| Connective tissue disease- no. (%) | 62 (3.62) | 49 (3.96) | 13 (2.72) | 0.216 |
| Stroke-associated pneumonia- no. (%) | 194 (11.32) | 122 (9.87) | 72 (15.06) | **0.002** |
| Admission medications |  |  |  |  |
| Anticoagulants- no. (%) | 34 (1.98) | 29 (2.35) | 5 (1.05) | 0.083 |
| Antiplatelets- no. (%) | 604 (35.24) | 430 (34.79) | 174 (36.4) | 0.531 |
| Biomarkers at admission |  |  |  |  |
| White cell count (x10^9/L) - median (IQR) | 9.90 (7.60-12.60) | 8.70 (7.00-10.50) | 12.90 (11.20-16.10) | **<0.001** |
| CRP (mg/L)- median (IQR) | 10.00 (4.00-28.00) | 6.00 (3.00-14.00) | 28.00 (15.00-56.00) | **<0.001** |
| Outcomes |  |  |  |  |
| Poor functional outcome- no. (%) | 1309 (76.37) | 916 (74.11) | 393 (82.22) | **<0.001** |
| mRS at discharge |  |  |  |  |
| Discharge mRS 0- no. (%) | 143 (8.34) | 114 (9.22) | 29 (6.07) | **<0.001** |
| Discharge mRS 1- no. (%) | 167 (9.74) | 133 (10.76) | 34 (7.11) | **<0.001** |
| Discharge mRS 2- no. (%) | 95 (5.54) | 73 (5.91) | 22 (4.6) | **<0.001** |
| Discharge mRS 3- no. (%) | 241 (14.06) | 181 (14.64) | 60 (12.55) | **<0.001** |
| Discharge mRS 4- no. (%) | 307 (17.91) | 228 (18.45) | 79 (16.53) | **<0.001** |
| Discharge mRS 5- no. (%) | 138 (8.05) | 97 (7.85) | 41 (8.58) | **<0.001** |
| Discharge mRS 6- no. (%) | 623 (36.35) | 410 (33.17) | 213 (44.56) | **<0.001** |
| Mortality |  |  |  |  |
| Death during admission- no. (%) | 623 (36.35) | 410 (33.17) | 213 (44.56) | **<0.001** |
| Death at 90 days- no. (%) | 714 (41.66) | 476 (38.51) | 238 (49.79) | **<0.001** |
| Death at 365 days- no. (%) | 834 (48.66) | 562 (45.47) | 272 (56.9) | **<0.001** |
| Length of stay |  |  |  |  |
| Days- median (IQR) | 8.00 (3.00-19.00) | 8.00 (3.00-19.00) | 8.00 (3.00-23.00) | 0.078 |
| Length of stay >14 days- no. (%) | 576 (33.61) | 396 (32.04) | 180 (37.66) | **0.027** |

Table S2.3

|  | ICH admissions | | |  |
| --- | --- | --- | --- | --- |
| Variable | Total cohort (*n*= 1714) | Non-elevated inflammatory biomarkers (n= 1234) | Elevated inflammatory biomarkers (n= 480) | *P-*value |
| Age- mean (+/- SD) | 76.05 (12.25) | 76.08 (12.24) | 75.99 (12.29) | 0.898 |
| Sex- no. male (%) | 854 (49.82) | 637 (51.62) | 217 (45.21) | **0.017** |
| Total NIHSS score- median (IQR) | 4.00 (1.00-9.00) | 4.00 (1.00-8.00) | 5.00 (2.00-10.00) | **0.037** |
| Pre-ICH mRS |  |  |  |  |
| 0- no. (%) | 1129 (65.87) | 827 (67.02) | 302 (62.92) | **0.022** |
| 1- no. (%) | 194 (11.32) | 138 (11.18) | 56 (11.67) | **0.022** |
| 2- no. (%) | 130 (7.58) | 102 (8.27) | 28 (5.83) | **0.022** |
| 3- no. (%) | 167 (9.74) | 110 (8.91) | 57 (11.88) | **0.022** |
| 4- no. (%) | 68 (3.97) | 40 (3.24) | 28 (5.83) | **0.022** |
| 5- no. (%) | 26 (1.52) | 17 (1.38) | 9 (1.88) | **0.022** |
| OCSP classification |  |  |  |  |
| TACS | 544 (31.74) | 354 (28.69) | 190 (39.58) | **<0.001** |
| PACS | 501 (29.23) | 376 (30.47) | 125 (26.04) | **<0.001** |
| LACS | 139 (8.11) | 106 (8.59) | 33 (6.88) | **<0.001** |
| POCS | 530 (30.92) | 398 (32.25) | 132 (27.5) | **<0.001** |
| Comorbidities |  |  |  |  |
| Asthma- no. (%) | 146 (8.52) | 115 (9.32) | 31 (6.46) | 0.057 |
| Atrial fibrillation- no. (%) | 383 (22.35) | 264 (21.39) | 119 (24.79) | 0.129 |
| Cerebral infarction- no. (%) | 135 (7.88) | 98 (7.94) | 37 (7.71) | 0.872 |
| Coronary heart disease- no. (%) | 327 (19.08) | 218 (17.67) | 109 (22.71) | **0.017** |
| Congestive heart failure- no. (%) | 135 (7.88) | 85 (6.89) | 50 (10.42) | **0.015** |
| Chronic kidney disease- no. (%) | 85 (4.96) | 56 (4.54) | 29 (6.04) | 0.198 |
| COPD- no. (%) | 104 (6.07) | 80 (6.48) | 24 (5) | 0.248 |
| Dementia- no. (%) | 71 (4.14) | 51 (4.13) | 20 (4.17) | 0.975 |
| Diabetes mellitus- no. (%) | 205 (11.96) | 148 (11.99) | 57 (11.88) | 0.946 |
| Hyperlipidemia- no. (%) | 179 (10.44) | 134 (10.86) | 45 (9.38) | 0.367 |
| Hypertension- no. (%) | 996 (58.11) | 726 (58.83) | 270 (56.25) | 0.330 |
| Liver disease- no. (%) | 26 (1.52) | 23 (1.86) | 3 (.63) | 0.060 |
| Malignancy- no. (%) | 243 (14.18) | 177 (14.34) | 66 (13.75) | 0.752 |
| Peptic ulcer disease- no. (%) | 64 (3.73) | 43 (3.48) | 21 (4.38) | 0.383 |
| Peripheral vascular disease- no. (%) | 82 (4.78) | 60 (4.86) | 22 (4.58) | 0.808 |
| Previous ICH/SAH- no. (%) | 377 (22) | 277 (22.45) | 100 (20.83) | 0.469 |
| Connective tissue disease- no. (%) | 62 (3.62) | 48 (3.89) | 14 (2.92) | 0.333 |
| Stroke-associated pneumonia- no. (%) | 194 (11.32) | 121 (9.81) | 73 (15.21) | **0.002** |
| Admission medications |  |  |  |  |
| Anticoagulants- no. (%) | 34 (1.98) | 29 (2.35) | 5 (1.04) | 0.081 |
| Antiplatelets- no. (%) | 604 (35.24) | 428 (34.68) | 176 (36.67) | 0.440 |
| Biomarkers at admission |  |  |  |  |
| White cell count (x10^9/L) - median (IQR) | 9.90 (7.60-12.60) | 8.70 (7.00-10.50) | 12.90 (11.20-16.25) | **<0.001** |
| CRP (mg/L)- median (IQR) | 10.00 (4.00-27.00) | 6.00 (4.00-13.00) | 27.00 (15.00-60.00) | **<0.001** |
| Outcomes |  |  |  |  |
| Poor functional outcome- no. (%) | 1329 (77.54) | 932 (75.53) | 397 (82.71) | **0.001** |
| mRS at discharge |  |  |  |  |
| Discharge mRS 0- no. (%) | 133 (7.76) | 103 (8.35) | 30 (6.25) | **0.005** |
| Discharge mRS 1- no. (%) | 154 (8.98) | 117 (9.48) | 37 (7.71) | **0.005** |
| Discharge mRS 2- no. (%) | 98 (5.72) | 82 (6.65) | 16 (3.33) | **0.005** |
| Discharge mRS 3- no. (%) | 258 (15.05) | 190 (15.4) | 68 (14.17) | **0.005** |
| Discharge mRS 4- no. (%) | 306 (17.85) | 223 (18.07) | 83 (17.29) | **0.005** |
| Discharge mRS 5- no. (%) | 142 (8.27) | 103 (8.35) | 39 (8.13) | **0.005** |
| Discharge mRS 6- no. (%) | 623 (36.35) | 416 (33.71) | 207 (43.13) | **0.005** |
| Mortality |  |  |  |  |
| Death during admission- no. (%) | 623 (36.35) | 416 (33.71) | 207 (43.13) | **<0.001** |
| Death at 90 days- no. (%) | 714 (41.66) | 482 (39.06) | 232 (48.33) | **<0.001** |
| Death at 365 days- no. (%) | 834 (48.66) | 568 (46.03) | 266 (55.42) | **<0.001** |
| Length of stay |  |  |  |  |
| Days- median (IQR) | 8.00 (3.00-19.00) | 8.00 (3.00-19.00) | 8.00 (3.00-22.50) | 0.134 |
| Length of stay >14 days- no. (%) | 576 (33.61) | 395 (32.01) | 181 (37.71) | **0.025** |

Table S2.4

|  | ICH admissions | | |  |
| --- | --- | --- | --- | --- |
| Variable | Total cohort (*n*= 1714) | Non-elevated inflammatory biomarkers (n= 1237) | Elevated inflammatory biomarkers (n= 477) | *P-*value |
| Age- mean (+/- SD) | 76.05 (12.25) | 75.96 (12.36) | 76.30 (11.97) | 0.607 |
| Sex- no. male (%) | 854 (49.82) | 637 (51.5) | 217 (45.49) | **0.026** |
| Total NIHSS score- median (IQR) | 4.00 (2.00-8.00) | 3.00 (2.00-7.00) | 5.00 (2.00-10.00) | **0.009** |
| Pre-ICH mRS |  |  |  |  |
| 0- no. (%) | 1133 (66.1) | 832 (67.26) | 301 (63.1) | **0.015** |
| 1- no. (%) | 194 (11.32) | 140 (11.32) | 54 (11.32) | **0.015** |
| 2- no. (%) | 131 (7.64) | 103 (8.33) | 28 (5.87) | **0.015** |
| 3- no. (%) | 163 (9.51) | 105 (8.49) | 58 (12.16) | **0.015** |
| 4- no. (%) | 66 (3.85) | 40 (3.23) | 26 (5.45) | **0.015** |
| 5- no. (%) | 27 (1.58) | 17 (1.37) | 10 (2.1) | **0.015** |
| OCSP classification |  |  |  |  |
| TACS | 549 (32.03) | 356 (28.78) | 193 (40.46) | **<0.001** |
| PACS | 503 (29.35) | 369 (29.83) | 134 (28.09) | **<0.001** |
| LACS | 137 (7.99) | 108 (8.73) | 29 (6.08) | **<0.001** |
| POCS | 525 (30.63) | 404 (32.66) | 121 (25.37) | **<0.001** |
| Comorbidities |  |  |  |  |
| Asthma- no. (%) | 146 (8.52) | 113 (9.14) | 33 (6.92) | 0.141 |
| Atrial fibrillation- no. (%) | 383 (22.35) | 263 (21.26) | 120 (25.16) | 0.083 |
| Cerebral infarction- no. (%) | 135 (7.88) | 99 (8) | 36 (7.55) | 0.753 |
| Coronary heart disease- no. (%) | 327 (19.08) | 217 (17.54) | 110 (23.06) | **0.009** |
| Congestive heart failure- no. (%) | 135 (7.88) | 86 (6.95) | 49 (10.27) | **0.022** |
| Chronic kidney disease- no. (%) | 85 (4.96) | 56 (4.53) | 29 (6.08) | 0.185 |
| COPD- no. (%) | 104 (6.07) | 80 (6.47) | 24 (5.03) | 0.264 |
| Dementia- no. (%) | 71 (4.14) | 52 (4.2) | 19 (3.98) | 0.837 |
| Diabetes mellitus- no. (%) | 205 (11.96) | 146 (11.8) | 59 (12.37) | 0.746 |
| Hyperlipidemia- no. (%) | 179 (10.44) | 134 (10.83) | 45 (9.43) | 0.396 |
| Hypertension- no. (%) | 996 (58.11) | 725 (58.61) | 271 (56.81) | 0.499 |
| Liver disease- no. (%) | 26 (1.52) | 24 (1.94) | 2 (.42) | **0.021** |
| Malignancy- no. (%) | 243 (14.18) | 179 (14.47) | 64 (13.42) | 0.575 |
| Peptic ulcer disease- no. (%) | 64 (3.73) | 44 (3.56) | 20 (4.19) | 0.534 |
| Peripheral vascular disease- no. (%) | 82 (4.78) | 59 (4.77) | 23 (4.82) | 0.964 |
| Previous ICH/SAH- no. (%) | 377 (22) | 281 (22.72) | 96 (20.13) | 0.246 |
| Connective tissue disease- no. (%) | 62 (3.62) | 50 (4.04) | 12 (2.52) | 0.129 |
| Stroke-associated pneumonia- no. (%) | 194 (11.32) | 121 (9.78) | 73 (15.3) | **0.001** |
| Admission medications |  |  |  |  |
| Anticoagulants- no. (%) | 34 (1.98) | 29 (2.34) | 5 (1.05) | 0.085 |
| Antiplatelets- no. (%) | 604 (35.24) | 425 (34.36) | 179 (37.53) | 0.218 |
| Biomarkers at admission |  |  |  |  |
| White cell count (x10^9/L) - median (IQR) | 9.90 (7.60-12.60) | 8.70 (7.00-10.50) | 12.90 (11.30-16.10) | **<0.001** |
| CRP (mg/L)- median (IQR) | 10.00 (4.00-28.00) | 6.00 (3.00-14.00) | 27.00 (15.00-59.00) | **<0.001** |
| Outcomes |  |  |  |  |
| Poor functional outcome- no. (%) | 1321 (77.07) | 920 (74.37) | 401 (84.07) | **<0.001** |
| mRS at discharge |  |  |  |  |
| Discharge mRS 0- no. (%) | 151 (8.81) | 122 (9.86) | 29 (6.08) | **<0.001** |
| Discharge mRS 1- no. (%) | 147 (8.58) | 120 (9.7) | 27 (5.66) | **<0.001** |
| Discharge mRS 2- no. (%) | 95 (5.54) | 75 (6.06) | 20 (4.19) | **<0.001** |
| Discharge mRS 3- no. (%) | 249 (14.53) | 180 (14.55) | 69 (14.47) | **<0.001** |
| Discharge mRS 4- no. (%) | 303 (17.68) | 225 (18.19) | 78 (16.35) | **<0.001** |
| Discharge mRS 5- no. (%) | 146 (8.52) | 100 (8.08) | 46 (9.64) | **<0.001** |
| Discharge mRS 6- no. (%) | 623 (36.35) | 415 (33.55) | 208 (43.61) | **<0.001** |
| Mortality |  |  |  |  |
| Death during admission- no. (%) | 623 (36.35) | 415 (33.55) | 208 (43.61) | **<0.001** |
| Death at 90 days- no. (%) | 714 (41.66) | 480 (38.8) | 234 (49.06) | **<0.001** |
| Death at 365 days- no. (%) | 834 (48.66) | 568 (45.92) | 266 (55.77) | **<0.001** |
| Length of stay |  |  |  |  |
| Days- median (IQR) | 8.00 (3.00-19.00) | 8.00 (3.00-19.00) | 8.00 (3.00-23.00) | 0.068 |
| Length of stay >14 days- no. (%) | 576 (33.61) | 394 (31.85) | 182 (38.16) | **0.013** |

Table S2.5

|  | ICH admissions | | |  |
| --- | --- | --- | --- | --- |
| Variable | Total cohort (*n*= 1714) | Non-elevated inflammatory biomarkers (n= 1232) | Elevated inflammatory biomarkers (n= 482) | *P-*value |
| Age- mean (+/- SD) | 76.05 (12.25) | 75.89 (12.43) | 76.46 (11.78) | 0.389 |
| Sex- no. male (%) | 854 (49.82) | 637 (51.7) | 217 (45.02) | **0.013** |
| Total NIHSS score- median (IQR) | 5.00 (2.00-11.00) | 4.00 (2.00-11.00) | 5.00 (2.00-11.00) | 0.508 |
| Pre-ICH mRS |  |  |  |  |
| 0- no. (%) | 1137 (66.34) | 830 (67.37) | 307 (63.69) | 0.061 |
| 1- no. (%) | 194 (11.32) | 140 (11.36) | 54 (11.2) | 0.061 |
| 2- no. (%) | 126 (7.35) | 97 (7.87) | 29 (6.02) | 0.061 |
| 3- no. (%) | 166 (9.68) | 108 (8.77) | 58 (12.03) | 0.061 |
| 4- no. (%) | 63 (3.68) | 38 (3.08) | 25 (5.19) | 0.061 |
| 5- no. (%) | 28 (1.63) | 19 (1.54) | 9 (1.87) | 0.061 |
| OCSP classification |  |  |  |  |
| TACS | 567 (33.08) | 366 (29.71) | 201 (41.7) | **<0.001** |
| PACS | 495 (28.88) | 365 (29.63) | 130 (26.97) | **<0.001** |
| LACS | 135 (7.88) | 108 (8.77) | 27 (5.6) | **<0.001** |
| POCS | 517 (30.16) | 393 (31.9) | 124 (25.73) | **<0.001** |
| Comorbidities |  |  |  |  |
| Asthma- no. (%) | 146 (8.52) | 111 (9.01) | 35 (7.26) | 0.244 |
| Atrial fibrillation- no. (%) | 383 (22.35) | 261 (21.19) | 122 (25.31) | 0.065 |
| Cerebral infarction- no. (%) | 135 (7.88) | 98 (7.95) | 37 (7.68) | 0.848 |
| Coronary heart disease- no. (%) | 327 (19.08) | 218 (17.69) | 109 (22.61) | **0.020** |
| Congestive heart failure- no. (%) | 135 (7.88) | 84 (6.82) | 51 (10.58) | **0.009** |
| Chronic kidney disease- no. (%) | 85 (4.96) | 57 (4.63) | 28 (5.81) | 0.311 |
| COPD- no. (%) | 104 (6.07) | 79 (6.41) | 25 (5.19) | 0.339 |
| Dementia- no. (%) | 71 (4.14) | 52 (4.22) | 19 (3.94) | 0.794 |
| Diabetes mellitus- no. (%) | 205 (11.96) | 147 (11.93) | 58 (12.03) | 0.954 |
| Hyperlipidemia- no. (%) | 179 (10.44) | 135 (10.96) | 44 (9.13) | 0.266 |
| Hypertension- no. (%) | 996 (58.11) | 721 (58.52) | 275 (57.05) | 0.579 |
| Liver disease- no. (%) | 26 (1.52) | 23 (1.87) | 3 (.62) | 0.058 |
| Malignancy- no. (%) | 243 (14.18) | 175 (14.2) | 68 (14.11) | 0.959 |
| Peptic ulcer disease- no. (%) | 64 (3.73) | 42 (3.41) | 22 (4.56) | 0.257 |
| Peripheral vascular disease- no. (%) | 82 (4.78) | 61 (4.95) | 21 (4.36) | 0.604 |
| Previous ICH/SAH- no. (%) | 377 (22) | 278 (22.56) | 99 (20.54) | 0.363 |
| Connective tissue disease- no. (%) | 62 (3.62) | 49 (3.98) | 13 (2.7) | 0.202 |
| Stroke-associated pneumonia- no. (%) | 194 (11.32) | 121 (9.82) | 73 (15.15) | **0.002** |
| Admission medications |  |  |  |  |
| Anticoagulants- no. (%) | 34 (1.98) | 29 (2.35) | 5 (1.04) | 0.079 |
| Antiplatelets- no. (%) | 604 (35.24) | 422 (34.25) | 182 (37.76) | 0.172 |
| Biomarkers at admission |  |  |  |  |
| White cell count (x10^9/L) - median (IQR) | 9.90 (7.60-12.60) | 8.70 (7.00-10.40) | 12.90 (11.30-16.10) | **<0.001** |
| CRP (mg/L)- median (IQR) | 10.00 (5.00-29.00) | 7.00 (4.00-15.00) | 28.00 (15.00-56.00) | **<0.001** |
| Outcomes |  |  |  |  |
| Poor functional outcome- no. (%) | 1315 (76.72) | 918 (74.51) | 397 (82.37) | **<0.001** |
| mRS at discharge |  |  |  |  |
| Discharge mRS 0- no. (%) | 152 (8.87) | 113 (9.17) | 39 (8.09) | **<0.001** |
| Discharge mRS 1- no. (%) | 146 (8.52) | 122 (9.9) | 24 (4.98) | **<0.001** |
| Discharge mRS 2- no. (%) | 101 (5.89) | 79 (6.41) | 22 (4.56) | **<0.001** |
| Discharge mRS 3- no. (%) | 257 (14.99) | 189 (15.34) | 68 (14.11) | **<0.001** |
| Discharge mRS 4- no. (%) | 297 (17.33) | 227 (18.43) | 70 (14.52) | **<0.001** |
| Discharge mRS 5- no. (%) | 138 (8.05) | 92 (7.47) | 46 (9.54) | **<0.001** |
| Discharge mRS 6- no. (%) | 623 (36.35) | 410 (33.28) | 213 (44.19) | **<0.001** |
| Mortality |  |  |  |  |
| Death during admission- no. (%) | 623 (36.35) | 410 (33.28) | 213 (44.19) | **<0.001** |
| Death at 90 days- no. (%) | 714 (41.66) | 473 (38.39) | 241 (50) | **<0.001** |
| Death at 365 days- no. (%) | 834 (48.66) | 560 (45.45) | 274 (56.85) | **<0.001** |
| Length of stay |  |  |  |  |
| Days- median (IQR) | 8.00 (3.00-19.00) | 8.00 (3.00-19.00) | 8.00 (3.00-23.00) | 0.091 |
| Length of stay >14 days- no. (%) | 576 (33.61) | 391 (31.74) | 185 (38.38) | **0.009** |

Table S2.6

|  | ICH admissions | | |  |
| --- | --- | --- | --- | --- |
| Variable | Total cohort (*n*= 1714) | Non-elevated inflammatory biomarkers (n= 1234) | Elevated inflammatory biomarkers (n= 480) | *P-*value |
| Age- mean (+/- SD) | 76.05 (12.25) | 75.96 (12.36) | 76.27 (11.96) | 0.638 |
| Sex- no. male (%) | 854 (49.82) | 636 (51.54) | 218 (45.42) | **0.023** |
| Total NIHSS score- median (IQR) | 4.00 (2.00-9.00) | 4.00 (2.00-9.00) | 4.00 (2.00-9.00) | 0.416 |
| Pre-ICH mRS |  |  |  |  |
| 0- no. (%) | 1145 (66.8) | 841 (68.15) | 304 (63.33) | **0.005** |
| 1- no. (%) | 188 (10.97) | 136 (11.02) | 52 (10.83) | **0.005** |
| 2- no. (%) | 127 (7.41) | 99 (8.02) | 28 (5.83) | **0.005** |
| 3- no. (%) | 162 (9.45) | 104 (8.43) | 58 (12.08) | **0.005** |
| 4- no. (%) | 67 (3.91) | 38 (3.08) | 29 (6.04) | **0.005** |
| 5- no. (%) | 25 (1.46) | 16 (1.3) | 9 (1.88) | **0.005** |
| OCSP classification |  |  |  |  |
| TACS | 565 (32.96) | 363 (29.42) | 202 (42.08) | **<0.001** |
| PACS | 502 (29.29) | 369 (29.9) | 133 (27.71) | **<0.001** |
| LACS | 128 (7.47) | 100 (8.1) | 28 (5.83) | **<0.001** |
| POCS | 519 (30.28) | 402 (32.58) | 117 (24.38) | **<0.001** |
| Comorbidities |  |  |  |  |
| Asthma- no. (%) | 146 (8.52) | 114 (9.24) | 32 (6.67) | 0.087 |
| Atrial fibrillation- no. (%) | 383 (22.35) | 262 (21.23) | 121 (25.21) | 0.076 |
| Cerebral infarction- no. (%) | 135 (7.88) | 99 (8.02) | 36 (7.5) | 0.718 |
| Coronary heart disease- no. (%) | 327 (19.08) | 218 (17.67) | 109 (22.71) | **0.017** |
| Congestive heart failure- no. (%) | 135 (7.88) | 84 (6.81) | 51 (10.63) | **0.008** |
| Chronic kidney disease- no. (%) | 85 (4.96) | 57 (4.62) | 28 (5.83) | 0.298 |
| COPD- no. (%) | 104 (6.07) | 81 (6.56) | 23 (4.79) | 0.168 |
| Dementia- no. (%) | 71 (4.14) | 51 (4.13) | 20 (4.17) | 0.975 |
| Diabetes mellitus- no. (%) | 205 (11.96) | 146 (11.83) | 59 (12.29) | 0.792 |
| Hyperlipidemia- no. (%) | 179 (10.44) | 136 (11.02) | 43 (8.96) | 0.210 |
| Hypertension- no. (%) | 996 (58.11) | 726 (58.83) | 270 (56.25) | 0.330 |
| Liver disease- no. (%) | 26 (1.52) | 23 (1.86) | 3 (.63) | 0.060 |
| Malignancy- no. (%) | 243 (14.18) | 178 (14.42) | 65 (13.54) | 0.638 |
| Peptic ulcer disease- no. (%) | 64 (3.73) | 42 (3.4) | 22 (4.58) | 0.247 |
| Peripheral vascular disease- no. (%) | 82 (4.78) | 59 (4.78) | 23 (4.79) | 0.993 |
| Previous ICH/SAH- no. (%) | 377 (22) | 280 (22.69) | 97 (20.21) | 0.265 |
| Connective tissue disease- no. (%) | 62 (3.62) | 48 (3.89) | 14 (2.92) | 0.333 |
| Stroke-associated pneumonia- no. (%) | 194 (11.32) | 120 (9.72) | 74 (15.42) | **<0.001** |
| Admission medications |  |  |  |  |
| Anticoagulants- no. (%) | 34 (1.98) | 29 (2.35) | 5 (1.04) | 0.081 |
| Antiplatelets- no. (%) | 604 (35.24) | 426 (34.52) | 178 (37.08) | 0.319 |
| Biomarkers at admission |  |  |  |  |
| White cell count (x10^9/L) - median (IQR) | 9.90 (7.60-12.60) | 8.70 (7.00-10.50) | 12.90 (11.20-16.15) | **<0.001** |
| CRP (mg/L)- median (IQR) | 10.00 (4.00-27.00) | 6.00 (3.00-14.00) | 26.00 (14.00-54.00) | **<0.001** |
| Outcomes |  |  |  |  |
| Poor functional outcome- no. (%) | 1327 (77.42) | 927 (75.12) | 400 (83.33) | **<0.001** |
| mRS at discharge |  |  |  |  |
| Discharge mRS 0- no. (%) | 137 (7.99) | 109 (8.83) | 28 (5.83) | **<0.001** |
| Discharge mRS 1- no. (%) | 154 (8.98) | 122 (9.89) | 32 (6.67) | **<0.001** |
| Discharge mRS 2- no. (%) | 96 (5.6) | 76 (6.16) | 20 (4.17) | **<0.001** |
| Discharge mRS 3- no. (%) | 246 (14.35) | 181 (14.67) | 65 (13.54) | **<0.001** |
| Discharge mRS 4- no. (%) | 311 (18.14) | 232 (18.8) | 79 (16.46) | **<0.001** |
| Discharge mRS 5- no. (%) | 147 (8.58) | 103 (8.35) | 44 (9.17) | **<0.001** |
| Discharge mRS 6- no. (%) | 623 (36.35) | 411 (33.31) | 212 (44.17) | **<0.001** |
| Mortality |  |  |  |  |
| Death during admission- no. (%) | 623 (36.35) | 411 (33.31) | 212 (44.17) | **<0.001** |
| Death at 90 days- no. (%) | 714 (41.66) | 477 (38.65) | 237 (49.38) | **<0.001** |
| Death at 365 days- no. (%) | 834 (48.66) | 565 (45.79) | 269 (56.04) | **<0.001** |
| Length of stay |  |  |  |  |
| Days- median (IQR) | 8.00 (3.00-19.00) | 8.00 (3.00-19.00) | 8.00 (3.00-22.50) | 0.063 |
| Length of stay >14 days- no. (%) | 576 (33.61) | 395 (32.01) | 181 (37.71) | **0.025** |

Table S2.7

|  | ICH admissions | | |  |
| --- | --- | --- | --- | --- |
| Variable | Total cohort (*n*= 1714) | Non-elevated inflammatory biomarkers (n= 1223) | Elevated inflammatory biomarkers (n= 491) | *P-*value |
| Age- mean (+/- SD) | 76.05 (12.25) | 76.07 (12.31) | 75.99 (12.10) | 0.903 |
| Sex- no. male (%) | 854 (49.82) | 629 (51.43) | 225 (45.82) | **0.036** |
| Total NIHSS score- median (IQR) | 4.00 (2.00-7.00) | 3.00 (2.00-7.00) | 4.00 (2.00-7.00) | 0.222 |
| Pre-ICH mRS |  |  |  |  |
| 0- no. (%) | 1143 (66.69) | 825 (67.46) | 318 (64.77) | **0.014** |
| 1- no. (%) | 185 (10.79) | 137 (11.2) | 48 (9.78) | **0.014** |
| 2- no. (%) | 130 (7.58) | 101 (8.26) | 29 (5.91) | **0.014** |
| 3- no. (%) | 163 (9.51) | 103 (8.42) | 60 (12.22) | **0.014** |
| 4- no. (%) | 68 (3.97) | 40 (3.27) | 28 (5.7) | **0.014** |
| 5- no. (%) | 25 (1.46) | 17 (1.39) | 8 (1.63) | **0.014** |
| OCSP classification |  |  |  |  |
| TACS | 569 (33.2) | 379 (30.99) | 190 (38.7) | **0.022** |
| PACS | 505 (29.46) | 370 (30.25) | 135 (27.49) | **0.022** |
| LACS | 138 (8.05) | 104 (8.5) | 34 (6.92) | **0.022** |
| POCS | 502 (29.29) | 370 (30.25) | 132 (26.88) | **0.022** |
| Comorbidities |  |  |  |  |
| Asthma- no. (%) | 146 (8.52) | 112 (9.16) | 34 (6.92) | 0.134 |
| Atrial fibrillation- no. (%) | 383 (22.35) | 261 (21.34) | 122 (24.85) | 0.115 |
| Cerebral infarction- no. (%) | 135 (7.88) | 99 (8.09) | 36 (7.33) | 0.596 |
| Coronary heart disease- no. (%) | 327 (19.08) | 219 (17.91) | 108 (22) | 0.051 |
| Congestive heart failure- no. (%) | 135 (7.88) | 84 (6.87) | 51 (10.39) | **0.014** |
| Chronic kidney disease- no. (%) | 85 (4.96) | 57 (4.66) | 28 (5.7) | 0.369 |
| COPD- no. (%) | 104 (6.07) | 81 (6.62) | 23 (4.68) | 0.129 |
| Dementia- no. (%) | 71 (4.14) | 51 (4.17) | 20 (4.07) | 0.928 |
| Diabetes mellitus- no. (%) | 205 (11.96) | 148 (12.1) | 57 (11.61) | 0.776 |
| Hyperlipidemia- no. (%) | 179 (10.44) | 133 (10.87) | 46 (9.37) | 0.357 |
| Hypertension- no. (%) | 996 (58.11) | 714 (58.38) | 282 (57.43) | 0.719 |
| Liver disease- no. (%) | 26 (1.52) | 23 (1.88) | 3 (.61) | 0.052 |
| Malignancy- no. (%) | 243 (14.18) | 176 (14.39) | 67 (13.65) | 0.689 |
| Peptic ulcer disease- no. (%) | 64 (3.73) | 42 (3.43) | 22 (4.48) | 0.302 |
| Peripheral vascular disease- no. (%) | 82 (4.78) | 59 (4.82) | 23 (4.68) | 0.902 |
| Previous ICH/SAH- no. (%) | 377 (22) | 277 (22.65) | 100 (20.37) | 0.302 |
| Connective tissue disease- no. (%) | 62 (3.62) | 48 (3.92) | 14 (2.85) | 0.282 |
| Stroke-associated pneumonia- no. (%) | 194 (11.32) | 120 (9.81) | 74 (15.07) | **0.002** |
| Admission medications |  |  |  |  |
| Anticoagulants- no. (%) | 34 (1.98) | 29 (2.37) | 5 (1.02) | 0.069 |
| Antiplatelets- no. (%) | 604 (35.24) | 423 (34.59) | 181 (36.86) | 0.372 |
| Biomarkers at admission |  |  |  |  |
| White cell count (x10^9/L) - median (IQR) | 9.90 (7.60-12.60) | 8.60 (7.00-10.40) | 12.90 (11.30-16.20) | **<0.001** |
| CRP (mg/L)- median (IQR) | 10.00 (4.00-29.00) | 6.00 (4.00-14.00) | 27.00 (14.00-60.00) | **<0.001** |
| Outcomes |  |  |  |  |
| Poor functional outcome- no. (%) | 1319 (76.95) | 914 (74.73) | 405 (82.48) | **<0.001** |
| mRS at discharge |  |  |  |  |
| Discharge mRS 0- no. (%) | 151 (8.81) | 115 (9.4) | 36 (7.33) | **0.002** |
| Discharge mRS 1- no. (%) | 146 (8.52) | 115 (9.4) | 31 (6.31) | **0.002** |
| Discharge mRS 2- no. (%) | 98 (5.72) | 79 (6.46) | 19 (3.87) | **0.002** |
| Discharge mRS 3- no. (%) | 255 (14.88) | 183 (14.96) | 72 (14.66) | **0.002** |
| Discharge mRS 4- no. (%) | 308 (17.97) | 229 (18.72) | 79 (16.09) | **0.002** |
| Discharge mRS 5- no. (%) | 133 (7.76) | 93 (7.6) | 40 (8.15) | **0.002** |
| Discharge mRS 6- no. (%) | 623 (36.35) | 409 (33.44) | 214 (43.58) | **0.002** |
| Mortality |  |  |  |  |
| Death during admission- no. (%) | 623 (36.35) | 409 (33.44) | 214 (43.58) | **<0.001** |
| Death at 90 days- no. (%) | 714 (41.66) | 475 (38.84) | 239 (48.68) | **<0.001** |
| Death at 365 days- no. (%) | 834 (48.66) | 562 (45.95) | 272 (55.4) | **<0.001** |
| Length of stay |  |  |  |  |
| Days- median (IQR) | 8.00 (3.00-19.00) | 8.00 (3.00-19.00) | 8.00 (3.00-22.00) | 0.316 |
| Length of stay >14 days- no. (%) | 576 (33.61) | 394 (32.22) | 182 (37.07) | 0.055 |

Table S2.8

|  | ICH admissions | | |  |
| --- | --- | --- | --- | --- |
| Variable | Total cohort (*n*= 1714) | Non-elevated inflammatory biomarkers (n= 1230) | Elevated inflammatory biomarkers (n= 484) | *P-*value |
| Age- mean (+/- SD) | 76.05 (12.25) | 75.93 (12.41) | 76.35 (11.84) | 0.526 |
| Sex- no. male (%) | 854 (49.82) | 631 (51.3) | 223 (46.07) | 0.051 |
| Total NIHSS score- median (IQR) | 4.00 (2.00-7.00) | 3.00 (1.00-7.00) | 5.00 (2.00-7.00) | **0.028** |
| Pre-ICH mRS |  |  |  |  |
| 0- no. (%) | 1136 (66.28) | 831 (67.56) | 305 (63.02) | **0.025** |
| 1- no. (%) | 193 (11.26) | 138 (11.22) | 55 (11.36) | **0.025** |
| 2- no. (%) | 130 (7.58) | 99 (8.05) | 31 (6.4) | **0.025** |
| 3- no. (%) | 164 (9.57) | 109 (8.86) | 55 (11.36) | **0.025** |
| 4- no. (%) | 64 (3.73) | 37 (3.01) | 27 (5.58) | **0.025** |
| 5- no. (%) | 27 (1.58) | 16 (1.3) | 11 (2.27) | **0.025** |
| OCSP classification |  |  |  |  |
| TACS | 564 (32.91) | 363 (29.51) | 201 (41.53) | **<0.001** |
| PACS | 491 (28.65) | 370 (30.08) | 121 (25) | **<0.001** |
| LACS | 131 (7.64) | 103 (8.37) | 28 (5.79) | **<0.001** |
| POCS | 528 (30.81) | 394 (32.03) | 134 (27.69) | **<0.001** |
| Comorbidities |  |  |  |  |
| Asthma- no. (%) | 146 (8.52) | 114 (9.27) | 32 (6.61) | 0.076 |
| Atrial fibrillation- no. (%) | 383 (22.35) | 259 (21.06) | 124 (25.62) | **0.041** |
| Cerebral infarction- no. (%) | 135 (7.88) | 98 (7.97) | 37 (7.64) | 0.823 |
| Coronary heart disease- no. (%) | 327 (19.08) | 215 (17.48) | 112 (23.14) | **0.007** |
| Congestive heart failure- no. (%) | 135 (7.88) | 86 (6.99) | 49 (10.12) | **0.030** |
| Chronic kidney disease- no. (%) | 85 (4.96) | 56 (4.55) | 29 (5.99) | 0.217 |
| COPD- no. (%) | 104 (6.07) | 80 (6.50) | 24 (4.96) | 0.228 |
| Dementia- no. (%) | 71 (4.14) | 52 (4.23) | 19 (3.93) | 0.778 |
| Diabetes mellitus- no. (%) | 205 (11.96) | 146 (11.87) | 59 (12.19) | 0.854 |
| Hyperlipidemia- no. (%) | 179 (10.44) | 135 (10.98) | 44 (9.09) | 0.251 |
| Hypertension- no. (%) | 996 (58.11) | 720 (58.54) | 276 (57.02) | 0.568 |
| Liver disease- no. (%) | 26 (1.52) | 23 (1.87) | 3 (.62) | 0.057 |
| Malignancy- no. (%) | 243 (14.18) | 175 (14.23) | 68 (14.05) | 0.924 |
| Peptic ulcer disease- no. (%) | 64 (3.73) | 41 (3.33) | 23 (4.75) | 0.163 |
| Peripheral vascular disease- no. (%) | 82 (4.78) | 61 (4.96) | 21 (4.34) | 0.588 |
| Previous ICH/SAH- no. (%) | 377 (22) | 278 (22.6) | 99 (20.45) | 0.334 |
| Connective tissue disease- no. (%) | 62 (3.62) | 50 (4.07) | 12 (2.48) | 0.113 |
| Stroke-associated pneumonia- no. (%) | 194 (11.32) | 121 (9.84) | 73 (15.08) | **0.002** |
| Admission medications |  |  |  |  |
| Anticoagulants- no. (%) | 34 (1.98) | 29 (2.36) | 5 (1.03) | 0.077 |
| Antiplatelets- no. (%) | 604 (35.24) | 422 (34.31) | 182 (37.6) | 0.199 |
| Biomarkers at admission |  |  |  |  |
| White cell count (x10^9/L) - median (IQR) | 9.90 (7.60-12.60) | 8.70 (7.00-10.50) | 12.90 (11.30-16.10) | **<0.001** |
| CRP (mg/L)- median (IQR) | 10.00 (4.00-28.00) | 6.00 (4.00-14.00) | 27.00 (15.00-60.00) | **<0.001** |
| Outcomes |  |  |  |  |
| Poor functional outcome- no. (%) | 1309 (76.37) | 909 (73.90) | 400 (82.64) | **<0.001** |
| mRS at discharge |  |  |  |  |
| Discharge mRS 0- no. (%) | 153 (8.93) | 114 (9.27) | 39 (8.06) | **<0.001** |
| Discharge mRS 1- no. (%) | 152 (8.87) | 126 (10.24) | 26 (5.37) | **<0.001** |
| Discharge mRS 2- no. (%) | 100 (5.83) | 81 (6.59) | 19 (3.93) | **<0.001** |
| Discharge mRS 3- no. (%) | 244 (14.24) | 176 (14.31) | 68 (14.05) | **<0.001** |
| Discharge mRS 4- no. (%) | 302 (17.62) | 226 (18.37) | 76 (15.7) | **<0.001** |
| Discharge mRS 5- no. (%) | 140 (8.17) | 99 (8.05) | 41 (8.47) | **<0.001** |
| Discharge mRS 6- no. (%) | 623 (36.35) | 408 (33.17) | 215 (44.42) | **<0.001** |
| Mortality |  |  |  |  |
| Death during admission- no. (%) | 623 (36.35) | 408 (33.17) | 215 (44.42) | **<0.001** |
| Death at 90 days- no. (%) | 714 (41.66) | 473 (38.46) | 241 (49.79) | **<0.001** |
| Death at 365 days- no. (%) | 834 (48.66) | 561 (45.61) | 273 (56.4) | **<0.001** |
| Length of stay |  |  |  |  |
| Days- median (IQR) | 8.00 (3.00-19.00) | 8.00 (3.00-19.00) | 8.00 (3.00-22.50) | 0.133 |
| Length of stay >14 days- no. (%) | 576 (33.61) | 393 (31.95) | 183 (37.81) | **0.021** |

Table S2.9

|  | ICH admissions | | |  |
| --- | --- | --- | --- | --- |
| Variable | Total cohort (*n*= 1714) | Non-elevated inflammatory biomarkers (n= 1231) | Elevated inflammatory biomarkers (n= 483) | *P-*value |
| Age- mean (+/- SD) | 76.05 (12.25) | 75.98 (12.30) | 76.22 (12.12) | 0.718 |
| Sex- no. male (%) | 854 (49.82) | 633 (51.42) | 221 (45.76) | **0.035** |
| Total NIHSS score- median (IQR) | 2.00 (1.00-8.00) | 2.00 (1.00-8.00) | 3.00 (1.00-9.00) | **0.022** |
| Pre-ICH mRS |  |  |  |  |
| 0- no. (%) | 1138 (66.39) | 829 (67.34) | 309 (63.98) | **0.004** |
| 1- no. (%) | 185 (10.79) | 135 (10.97) | 50 (10.35) | **0.004** |
| 2- no. (%) | 124 (7.23) | 99 (8.04) | 25 (5.18) | **0.004** |
| 3- no. (%) | 171 (9.98) | 112 (9.1) | 59 (12.22) | **0.004** |
| 4- no. (%) | 71 (4.14) | 40 (3.25) | 31 (6.42) | **0.004** |
| 5- no. (%) | 25 (1.46) | 16 (1.3) | 9 (1.86) | **0.004** |
| OCSP classification |  |  |  |  |
| TACS | 567 (33.08) | 364 (29.57) | 203 (42.03) | **<0.001** |
| PACS | 487 (28.41) | 361 (29.33) | 126 (26.09) | **<0.001** |
| LACS | 136 (7.93) | 103 (8.37) | 33 (6.83) | **<0.001** |
| POCS | 524 (30.57) | 403 (32.74) | 121 (25.05) | **<0.001** |
| Comorbidities |  |  |  |  |
| Asthma- no. (%) | 146 (8.52) | 114 (9.26) | 32 (6.63) | 0.079 |
| Atrial fibrillation- no. (%) | 383 (22.35) | 264 (21.45) | 119 (24.64) | 0.154 |
| Cerebral infarction- no. (%) | 135 (7.88) | 99 (8.04) | 36 (7.45) | 0.684 |
| Coronary heart disease- no. (%) | 327 (19.08) | 217 (17.63) | 110 (22.77) | **0.015** |
| Congestive heart failure- no. (%) | 135 (7.88) | 85 (6.9) | 50 (10.35) | **0.017** |
| Chronic kidney disease- no. (%) | 85 (4.96) | 56 (4.55) | 29 (6) | 0.212 |
| COPD- no. (%) | 104 (6.07) | 80 (6.5) | 24 (4.97) | 0.233 |
| Dementia- no. (%) | 71 (4.14) | 51 (4.14) | 20 (4.14) | 0.998 |
| Diabetes mellitus- no. (%) | 205 (11.96) | 149 (12.1) | 56 (11.59) | 0.770 |
| Hyperlipidemia- no. (%) | 179 (10.44) | 134 (10.89) | 45 (9.32) | 0.339 |
| Hypertension- no. (%) | 996 (58.11) | 720 (58.49) | 276 (57.14) | 0.611 |
| Liver disease- no. (%) | 26 (1.52) | 23 (1.87) | 3 (.62) | 0.057 |
| Malignancy- no. (%) | 243 (14.18) | 178 (14.46) | 65 (13.46) | 0.593 |
| Peptic ulcer disease- no. (%) | 64 (3.73) | 41 (3.33) | 23 (4.76) | 0.160 |
| Peripheral vascular disease- no. (%) | 82 (4.78) | 61 (4.96) | 21 (4.35) | 0.596 |
| Previous ICH/SAH- no. (%) | 377 (22) | 278 (22.58) | 99 (20.5) | 0.348 |
| Connective tissue disease- no. (%) | 62 (3.62) | 48 (3.9) | 14 (2.9) | 0.318 |
| Stroke-associated pneumonia- no. (%) | 194 (11.32) | 122 (9.91) | 72 (14.91) | **0.003** |
| Admission medications |  |  |  |  |
| Anticoagulants- no. (%) | 34 (1.98) | 29 (2.36) | 5 (1.04) | 0.078 |
| Antiplatelets- no. (%) | 604 (35.24) | 427 (34.69) | 177 (36.65) | 0.445 |
| Biomarkers at admission |  |  |  |  |
| White cell count (x10^9/L) - median (IQR) | 9.90 (7.60-12.60) | 8.70 (7.00-10.50) | 12.90 (11.20-16.20) | **<0.001** |
| CRP (mg/L)- median (IQR) | 10.00 (4.00-28.00) | 6.00 (3.00-14.00) | 27.00 (15.00-55.00) | **<0.001** |
| Outcomes |  |  |  |  |
| Poor functional outcome- no. (%) | 1320 (77.01) | 917 (74.49) | 403 (83.44) | **<0.001** |
| mRS at discharge |  |  |  |  |
| Discharge mRS 0- no. (%) | 149 (8.69) | 119 (9.67) | 30 (6.21) | **<0.001** |
| Discharge mRS 1- no. (%) | 147 (8.58) | 120 (9.75) | 27 (5.59) | **<0.001** |
| Discharge mRS 2- no. (%) | 98 (5.72) | 75 (6.09) | 23 (4.76) | **<0.001** |
| Discharge mRS 3- no. (%) | 250 (14.59) | 182 (14.78) | 68 (14.08) | **<0.001** |
| Discharge mRS 4- no. (%) | 307 (17.91) | 231 (18.77) | 76 (15.73) | **<0.001** |
| Discharge mRS 5- no. (%) | 140 (8.17) | 94 (7.64) | 46 (9.52) | **<0.001** |
| Discharge mRS 6- no. (%) | 623 (36.35) | 410 (33.31) | 213 (44.1) | **<0.001** |
| Mortality |  |  |  |  |
| Death during admission- no. (%) | 623 (36.35) | 410 (33.31) | 213 (44.1) | **<0.001** |
| Death at 90 days- no. (%) | 714 (41.66) | 475 (38.59) | 239 (49.48) | **<0.001** |
| Death at 365 days- no. (%) | 834 (48.66) | 562 (45.65) | 272 (56.31) | **<0.001** |
| Length of stay |  |  |  |  |
| Days- median (IQR) | 8.00 (3.00-19.00) | 8.00 (3.00-19.00) | 8.00 (3.00-23.00) | 0.100 |
| Length of stay >14 days- no. (%) | 576 (33.61) | 393 (31.93) | 183 (37.89) | **0.019** |

Table S2.10

|  | ICH admissions | | |  |
| --- | --- | --- | --- | --- |
| Variable | Total cohort (*n*= 1714) | Non-elevated inflammatory biomarkers (n= 1229) | Elevated inflammatory biomarkers (n= 485) | *P-*value |
| Age- mean (+/- SD) | 76.05 (12.25) | 75.90 (12.33) | 76.44 (12.03) | 0.414 |
| Sex- no. male (%) | 854 (49.82) | 631 (51.34) | 223 (45.98) | **0.045** |
| Total NIHSS score- median (IQR) | 4.00 (2.00-10.00) | 4.00 (2.00-10.00) | 3.00 (1.00-10.00) | 0.613 |
| Pre-ICH mRS |  |  |  |  |
| 0- no. (%) | 1137 (66.34) | 833 (67.78) | 304 (62.68) | **0.001** |
| 1- no. (%) | 194 (11.32) | 137 (11.15) | 57 (11.75) | **0.001** |
| 2- no. (%) | 129 (7.53) | 103 (8.38) | 26 (5.36) | **0.001** |
| 3- no. (%) | 164 (9.57) | 104 (8.46) | 60 (12.37) | **0.001** |
| 4- no. (%) | 65 (3.79) | 36 (2.93) | 29 (5.98) | **0.001** |
| 5- no. (%) | 25 (1.46) | 16 (1.3) | 9 (1.86) | **0.001** |
| OCSP classification |  |  |  |  |
| TACS | 576 (33.61) | 372 (30.27) | 204 (42.06) | **<0.001** |
| PACS | 505 (29.46) | 380 (30.92) | 125 (25.77) | **<0.001** |
| LACS | 131 (7.64) | 97 (7.89) | 34 (7.01) | **<0.001** |
| POCS | 502 (29.29) | 380 (30.92) | 122 (25.15) | **<0.001** |
| Comorbidities |  |  |  |  |
| Asthma- no. (%) | 146 (8.52) | 114 (9.28) | 32 (6.6) | 0.074 |
| Atrial fibrillation- no. (%) | 383 (22.35) | 259 (21.07) | 124 (25.57) | **0.044** |
| Cerebral infarction- no. (%) | 135 (7.88) | 98 (7.97) | 37 (7.63) | 0.811 |
| Coronary heart disease- no. (%) | 327 (19.08) | 214 (17.41) | 113 (23.3) | **0.005** |
| Congestive heart failure- no. (%) | 135 (7.88) | 85 (6.92) | 50 (10.31) | **0.019** |
| Chronic kidney disease- no. (%) | 85 (4.96) | 56 (4.56) | 29 (5.98) | 0.222 |
| COPD- no. (%) | 104 (6.07) | 81 (6.59) | 23 (4.74) | 0.149 |
| Dementia- no. (%) | 71 (4.14) | 51 (4.15) | 20 (4.12) | 0.981 |
| Diabetes mellitus- no. (%) | 205 (11.96) | 146 (11.88) | 59 (12.16) | 0.870 |
| Hyperlipidemia- no. (%) | 179 (10.44) | 135 (10.98) | 44 (9.07) | 0.244 |
| Hypertension- no. (%) | 996 (58.11) | 717 (58.34) | 279 (57.53) | 0.758 |
| Liver disease- no. (%) | 26 (1.52) | 23 (1.87) | 3 (.62) | 0.056 |
| Malignancy- no. (%) | 243 (14.18) | 175 (14.24) | 68 (14.02) | 0.907 |
| Peptic ulcer disease- no. (%) | 64 (3.73) | 43 (3.5) | 21 (4.33) | 0.414 |
| Peripheral vascular disease- no. (%) | 82 (4.78) | 60 (4.88) | 22 (4.54) | 0.762 |
| Previous ICH/SAH- no. (%) | 377 (22) | 278 (22.62) | 99 (20.41) | 0.320 |
| Connective tissue disease- no. (%) | 62 (3.62) | 48 (3.91) | 14 (2.89) | 0.309 |
| Stroke-associated pneumonia- no. (%) | 194 (11.32) | 119 (9.68) | 75 (15.46) | **<0.001** |
| Admission medications |  |  |  |  |
| Anticoagulants- no. (%) | 34 (1.98) | 29 (2.36) | 5 (1.03) | 0.076 |
| Antiplatelets- no. (%) | 604 (35.24) | 425 (34.58) | 179 (36.91) | 0.364 |
| Biomarkers at admission |  |  |  |  |
| White cell count (x10^9/L) - median (IQR) | 9.90 (7.60-12.60) | 8.70 (7.00-10.40) | 12.90 (11.30-16.10) | **<0.001** |
| CRP (mg/L)- median (IQR) | 10.00 (4.00-28.00) | 6.00 (4.00-13.00) | 28.00 (15.00-59.00) | **<0.001** |
| Outcomes |  |  |  |  |
| Poor functional outcome- no. (%) | 1308 (76.31) | 896 (72.9) | 412 (84.95) | **<0.001** |
| mRS at discharge |  |  |  |  |
| Discharge mRS 0- no. (%) | 146 (8.52) | 122 (9.93) | 24 (4.95) | **<0.001** |
| Discharge mRS 1- no. (%) | 147 (8.58) | 123 (10.01) | 24 (4.95) | **<0.001** |
| Discharge mRS 2- no. (%) | 113 (6.59) | 88 (7.16) | 25 (5.15) | **<0.001** |
| Discharge mRS 3- no. (%) | 242 (14.12) | 172 (14) | 70 (14.43) | **<0.001** |
| Discharge mRS 4- no. (%) | 301 (17.56) | 222 (18.06) | 79 (16.29) | **<0.001** |
| Discharge mRS 5- no. (%) | 142 (8.289) | 93 (7.57) | 49 (10.1) | **<0.001** |
| Discharge mRS 6- no. (%) | 623 (36.35) | 409 (33.28) | 214 (44.12) | **<0.001** |
| Mortality |  |  |  |  |
| Death during admission- no. (%) | 623 (36.35) | 409 (33.28) | 214 (44.12) | **<0.001** |
| Death at 90 days- no. (%) | 714 (41.66) | 473 (38.49) | 241 (49.69) | **<0.001** |
| Death at 365 days- no. (%) | 834 (48.66) | 560 (45.57) | 274 (56.49) | **<0.001** |
| Length of stay |  |  |  |  |
| Days- median (IQR) | 8.00 (3.00-19.00) | 8.00 (3.00-19.00) | 8.00 (3.00-22.00) | 0.173 |
| Length of stay >14 days- no. (%) | 576 (33.61) | 394 (32.06) | 182 (37.53) | **0.031** |

Table S2.11

|  | ICH admissions | | |  |
| --- | --- | --- | --- | --- |
| Variable | Total cohort (*n*= 1714) | Non-elevated inflammatory biomarkers (n= 1233) | Elevated inflammatory biomarkers (n= 481) | *P-*value |
| Age- mean (+/- SD) | 76.05 (12.25) | 75.88 (12.39) | 76.49 (11.87) | 0.357 |
| Sex- no. male (%) | 854 (49.82) | 636 (51.58) | 218 (45.32) | **0.020** |
| Total NIHSS score- median (IQR) | 3.00 (1.00-8.00) | 4.00 (1.00-8.00) | 3.00 (1.00-9.00) | 0.918 |
| Pre-ICH mRS |  |  |  |  |
| 0- no. (%) | 1142 (66.63) | 832 (67.48) | 310 (64.45) | **0.048** |
| 1- no. (%) | 192 (11.2) | 143 (11.6) | 49 (10.19) | **0.048** |
| 2- no. (%) | 126 (7.35) | 96 (7.79) | 30 (6.24) | **0.048** |
| 3- no. (%) | 161 (9.39) | 105 (8.52) | 56 (11.64) | **0.048** |
| 4- no. (%) | 68 (3.97) | 41 (3.33) | 27 (5.61) | **0.048** |
| 5- no. (%) | 25 (1.46) | 16 (1.3) | 9 (1.87) | **0.048** |
| OCSP classification |  |  |  |  |
| TACS | 548 (31.97) | 361 (29.28) | 187 (38.88) | **0.002** |
| PACS | 494 (28.82) | 365 (29.6) | 129 (26.82) | **0.002** |
| LACS | 128 (7.47) | 97 (7.87) | 31 (6.44) | **0.002** |
| POCS | 544 (31.74) | 410 (33.25) | 134 (27.86) | **0.002** |
| Comorbidities |  |  |  |  |
| Asthma- no. (%) | 146 (8.52) | 114 (9.25) | 32 (6.65) | 0.084 |
| Atrial fibrillation- no. (%) | 383 (22.35) | 259 (21.01) | 124 (25.78) | **0.033** |
| Cerebral infarction- no. (%) | 135 (7.88) | 99 (8.03) | 36 (7.48) | 0.707 |
| Coronary heart disease- no. (%) | 327 (19.08) | 218 (17.68) | 109 (22.66) | **0.018** |
| Congestive heart failure- no. (%) | 135 (7.88) | 85 (6.89) | 50 (10.4) | **0.016** |
| Chronic kidney disease- no. (%) | 85 (4.96) | 56 (4.54) | 29 (6.03) | 0.203 |
| COPD- no. (%) | 104 (6.07) | 80 (6.49) | 24 (4.99) | 0.243 |
| Dementia- no. (%) | 71 (4.14) | 50 (4.06) | 21 (4.37) | 0.772 |
| Diabetes mellitus- no. (%) | 205 (11.96) | 148 (12) | 57 (11.85) | 0.930 |
| Hyperlipidemia- no. (%) | 179 (10.44) | 132 (10.71) | 47 (9.77) | 0.570 |
| Hypertension- no. (%) | 996 (58.11) | 722 (58.56) | 274 (56.96) | 0.548 |
| Liver disease- no. (%) | 26 (1.52) | 23 (1.87) | 3 (.62) | 0.059 |
| Malignancy- no. (%) | 243 (14.18) | 177 (14.36) | 66 (13.72) | 0.735 |
| Peptic ulcer disease- no. (%) | 64 (3.73) | 43 (3.49) | 21 (4.37) | 0.389 |
| Peripheral vascular disease- no. (%) | 82 (4.78) | 59 (4.79) | 23 (4.78) | 0.998 |
| Previous ICH/SAH- no. (%) | 377 (22) | 281 (22.79) | 96 (19.96) | 0.204 |
| Connective tissue disease- no. (%) | 62 (3.62) | 49 (3.97) | 13 (2.7) | 0.205 |
| Stroke-associated pneumonia- no. (%) | 194 (11.32) | 120 (9.73) | 74 (15.38) | **<0.001** |
| Admission medications |  |  |  |  |
| Anticoagulants- no. (%) | 34 (1.98) | 29 (2.35) | 5 (1.04) | 0.080 |
| Antiplatelets- no. (%) | 604 (35.24) | 426 (34.55) | 178 (37.01) | 0.339 |
| Biomarkers at admission |  |  |  |  |
| White cell count (x10^9/L) - median (IQR) | 9.90 (7.60-12.60) | 8.70 (7.00-10.50) | 12.90 (11.30-16.10) | **<0.001** |
| CRP (mg/L)- median (IQR) | 10.00 (5.00-29.00) | 6.00 (4.00-14.00) | 28.00 (15.00-55.00) | **<0.001** |
| Outcomes |  |  |  |  |
| Poor functional outcome- no. (%) | 1321 (77.07) | 914 (74.13) | 407 (84.62) | **<0.001** |
| mRS at discharge |  |  |  |  |
| Discharge mRS 0- no. (%) | 148 (8.63) | 122 (9.89) | 26 (5.41) | **<0.001** |
| Discharge mRS 1- no. (%) | 151 (8.81) | 125 (10.14) | 26 (5.41) | **<0.001** |
| Discharge mRS 2- no. (%) | 94 (5.48) | 72 (5.84) | 22 (4.57) | **<0.001** |
| Discharge mRS 3- no. (%) | 243 (14.18) | 176 (14.27) | 67 (13.93) | **<0.001** |
| Discharge mRS 4- no. (%) | 310 (18.09) | 230 (18.65) | 80 (16.63) | **<0.001** |
| Discharge mRS 5- no. (%) | 145 (8.46) | 101 (8.19) | 44 (9.15) | **<0.001** |
| Discharge mRS 6- no. (%) | 623 (36.35) | 407 (33.01) | 216 (44.91) | **<0.001** |
| Mortality |  |  |  |  |
| Death during admission- no. (%) | 623 (36.35) | 407 (33.01) | 216 (44.91) | **<0.001** |
| Death at 90 days- no. (%) | 714 (41.66) | 474 (38.44) | 240 (49.9) | **<0.001** |
| Death at 365 days- no. (%) | 834 (48.66) | 561 (45.5) | 273 (56.76) | **<0.001** |
| Length of stay |  |  |  |  |
| Days- median (IQR) | 8.00 (3.00-19.00) | 8.00 (3.00-19.00) | 8.00 (3.00-22.00) | 0.093 |
| Length of stay >14 days- no. (%) | 576 (33.61) | 396 (32.12) | 180 (37.42) | **0.037** |

Table S2.12

|  | ICH admissions | | |  |
| --- | --- | --- | --- | --- |
| Variable | Total cohort (*n*= 1714) | Non-elevated inflammatory biomarkers (n= 1230) | Elevated inflammatory biomarkers (n= 484) | *P-*value |
| Age- mean (+/- SD) | 76.05 (12.25) | 76.06 (12.24) | 76.02 (12.27) | 0.943 |
| Sex- no. male (%) | 854 (49.82) | 636 (51.71) | 218 (45.04) | **0.013** |
| Total NIHSS score- median (IQR) | 5.00 (2.00-6.00) | 5.00 (2.00-6.00) | 5.00 (2.00-7.00) | 0.350 |
| Pre-ICH mRS |  |  |  |  |
| 0- no. (%) | 1137 (66.34) | 828 (67.32) | 309 (63.84) | 0.088 |
| 1- no. (%) | 189 (11.03) | 137 (11.14) | 52 (10.74) | 0.088 |
| 2- no. (%) | 129 (7.53) | 98 (7.97) | 31 (6.4) | 0.088 |
| 3- no. (%) | 164 (9.57) | 108 (8.78) | 56 (11.57) | 0.088 |
| 4- no. (%) | 70 (4.08) | 42 (3.41) | 28 (5.79) | 0.088 |
| 5- no. (%) | 25 (1.46) | 17 (1.38) | 8 (1.65) | 0.088 |
| OCSP classification |  |  |  |  |
| TACS | 577 (33.66) | 381 (30.98) | 196 (40.5) | **0.003** |
| PACS | 480 (28) | 356 (28.94) | 124 (25.62) | **0.003** |
| LACS | 139 (8.11) | 103 (8.37) | 36 (7.44) | **0.003** |
| POCS | 518 (30.22) | 390 (31.71) | 128 (26.45) | **0.003** |
| Comorbidities |  |  |  |  |
| Asthma- no. (%) | 146 (8.52) | 113 (9.19) | 33 (6.82) | 0.114 |
| Atrial fibrillation- no. (%) | 383 (22.35) | 257 (20.89) | 126 (26.03) | **0.022** |
| Cerebral infarction- no. (%) | 135 (7.88) | 98 (7.97) | 37 (7.64) | 0.823 |
| Coronary heart disease- no. (%) | 327 (19.08) | 218 (17.72) | 109 (22.52) | **0.023** |
| Congestive heart failure- no. (%) | 135 (7.88) | 84 (6.83) | 51 (10.54) | **0.010** |
| Chronic kidney disease- no. (%) | 85 (4.96) | 57 (4.63) | 28 (5.79) | 0.323 |
| COPD- no. (%) | 104 (6.07) | 81 (6.59) | 23 (4.75) | 0.152 |
| Dementia- no. (%) | 71 (4.14) | 51 (4.15) | 20 (4.13) | 0.989 |
| Diabetes mellitus- no. (%) | 205 (11.96) | 147 (11.95) | 58 (11.98) | 0.985 |
| Hyperlipidemia- no. (%) | 179 (10.44) | 132 (10.73) | 47 (9.71) | 0.534 |
| Hypertension- no. (%) | 996 (58.11) | 720 (58.54) | 276 (57.02) | 0.568 |
| Liver disease- no. (%) | 26 (1.52) | 23 (1.87) | 3 (.62) | 0.057 |
| Malignancy- no. (%) | 243 (14.18) | 179 (14.55) | 64 (13.22) | 0.477 |
| Peptic ulcer disease- no. (%) | 64 (3.73) | 43 (3.5) | 21 (4.34) | 0.407 |
| Peripheral vascular disease- no. (%) | 82 (4.78) | 59 (4.8) | 23 (4.75) | 0.969 |
| Previous ICH/SAH- no. (%) | 377 (22) | 275 (22.36) | 102 (21.07) | 0.564 |
| Connective tissue disease- no. (%) | 62 (3.62) | 49 (3.98) | 13 (2.69) | 0.195 |
| Stroke-associated pneumonia- no. (%) | 194 (11.32) | 123 (10) | 71 (14.67) | **0.006** |
| Admission medications |  |  |  |  |
| Anticoagulants- no. (%) | 34 (1.98) | 29 (2.36) | 5 (1.03) | 0.077 |
| Antiplatelets- no. (%) | 604 (35.24) | 424 (34.47) | 180 (37.19) | 0.289 |
| Biomarkers at admission |  |  |  |  |
| White cell count (x10^9/L) - median (IQR) | 9.90 (7.60-12.60) | 8.70 (7.00-10.40) | 12.90 (11.30-16.10) | **<0.001** |
| CRP (mg/L)- median (IQR) | 10.00 (4.00-28.00) | 6.00 (3.00-14.00) | 28.00 (15.00-59.00) | **<0.001** |
| Outcomes |  |  |  |  |
| Poor functional outcome- no. (%) | 1315 (76.72) | 921 (74.88) | 394 (81.4) | **0.004** |
| mRS at discharge |  |  |  |  |
| Discharge mRS 0- no. (%) | 141 (8.23) | 109 (8.86) | 32 (6.61) | **<0.001** |
| Discharge mRS 1- no. (%) | 153 (8.93) | 125 (10.16) | 28 (5.79) | **<0.001** |
| Discharge mRS 2- no. (%) | 105 (6.13) | 75 (6.1) | 30 (6.2) | **<0.001** |
| Discharge mRS 3- no. (%) | 251 (14.64) | 175 (14.23) | 76 (15.7) | **<0.001** |
| Discharge mRS 4- no. (%) | 296 (17.27) | 230 (18.7) | 66 (13.64) | **<0.001** |
| Discharge mRS 5- no. (%) | 145 (8.46) | 104 (8.46) | 41 (8.47) | **<0.001** |
| Discharge mRS 6- no. (%) | 623 (36.35) | 412 (33.5) | 211 (43.6) | **<0.001** |
| Mortality |  |  |  |  |
| Death during admission- no. (%) | 623 (36.35) | 412 (33.5) | 211 (43.6) | **<0.001** |
| Death at 90 days- no. (%) | 714 (41.66) | 476 (38.7) | 238 (49.17) | **<0.001** |
| Death at 365 days- no. (%) | 834 (48.66) | 563 (45.77) | 271 (55.99) | **<0.001** |
| Length of stay |  |  |  |  |
| Days- median (IQR) | 8.00 (3.00-19.00) | 8.00 (3.00-19.00) | 8.00 (3.00-22.00) | 0.169 |
| Length of stay >14 days- no. (%) | 576 (33.61) | 395 (32.11) | 181 (37.4) | **0.037** |

Table S2.13

|  | ICH admissions | | |  |
| --- | --- | --- | --- | --- |
| Variable | Total cohort (*n*= 1714) | Non-elevated inflammatory biomarkers (n= 1235) | Elevated inflammatory biomarkers (n= 479) | *P-*value |
| Age- mean (+/- SD) | 76.05 (12.25) | 76.02 (12.39) | 76.14 (11.87) | 0.852 |
| Sex- no. male (%) | 854 (49.82) | 636 (51.5) | 218 (45.51) | **0.026** |
| Total NIHSS score- median (IQR) | 2.00 (0.00-6.00) | 2.00 (0.00-6.00) | 3.00 (0.00-7.00) | 0.403 |
| Pre-ICH mRS |  |  |  |  |
| 0- no. (%) | 1137 (66.34) | 829 (67.13) | 308 (64.3) | **0.004** |
| 1- no. (%) | 191 (11.14) | 135 (10.93) | 56 (11.69) | **0.004** |
| 2- no. (%) | 126 (7.35) | 104 (8.42) | 22 (4.59) | **0.004** |
| 3- no. (%) | 164 (9.57) | 108 (8.74) | 56 (11.69) | **0.004** |
| 4- no. (%) | 69 (4.03) | 40 (3.24) | 29 (6.05) | **0.004** |
| 5- no. (%) | 27 (1.58) | 19 (1.54) | 8 (1.67) | **0.004** |
| OCSP classification |  |  |  |  |
| TACS | 555 (32.38) | 367 (29.72) | 188 (39.25) | **0.002** |
| PACS | 494 (28.82) | 366 (29.64) | 128 (26.72) | **0.002** |
| LACS | 138 (8.05) | 104 (8.42) | 34 (7.1) | **0.002** |
| POCS | 527 (30.75) | 398 (32.23) | 129 (26.93) | **0.002** |
| Comorbidities |  |  |  |  |
| Asthma- no. (%) | 146 (8.52) | 114 (9.23) | 32 (6.68) | 0.090 |
| Atrial fibrillation- no. (%) | 383 (22.35) | 261 (21.13) | 122 (25.47) | 0.053 |
| Cerebral infarction- no. (%) | 135 (7.88) | 98 (7.94) | 37 (7.72) | 0.884 |
| Coronary heart disease- no. (%) | 327 (19.08) | 220 (17.81) | 107 (22.34) | **0.032** |
| Congestive heart failure- no. (%) | 135 (7.88) | 88 (7.13) | 47 (9.81) | 0.064 |
| Chronic kidney disease- no. (%) | 85 (4.96) | 57 (4.62) | 28 (5.85) | 0.292 |
| COPD- no. (%) | 104 (6.07) | 80 (6.48) | 24 (5.01) | 0.254 |
| Dementia- no. (%) | 71 (4.14) | 50 (4.05) | 21 (4.38) | 0.754 |
| Diabetes mellitus- no. (%) | 205 (11.96) | 150 (12.15) | 55 (11.48) | 0.704 |
| Hyperlipidemia- no. (%) | 179 (10.44) | 134 (10.85) | 45 (9.39) | 0.377 |
| Hypertension- no. (%) | 996 (58.11) | 722 (58.46) | 274 (57.2) | 0.635 |
| Liver disease- no. (%) | 26 (1.52) | 23 (1.86) | 3 (.63) | 0.060 |
| Malignancy- no. (%) | 243 (14.18) | 177 (14.33) | 66 (13.78) | 0.768 |
| Peptic ulcer disease- no. (%) | 64 (3.73) | 42 (3.4) | 22 (4.59) | 0.243 |
| Peripheral vascular disease- no. (%) | 82 (4.78) | 61 (4.94) | 21 (4.38) | 0.629 |
| Previous ICH/SAH- no. (%) | 377 (22) | 276 (22.35) | 101 (21.09) | 0.571 |
| Connective tissue disease- no. (%) | 62 (3.62) | 48 (3.89) | 14 (2.92) | 0.338 |
| Stroke-associated pneumonia- no. (%) | 194 (11.32) | 123 (9.96) | 71 (14.82) | **0.004** |
| Biomarkers at admission |  |  |  |  |
| Anticoagulants- no. (%) | 34 (1.98) | 29 (2.35) | 5 (1.04) | 0.082 |
| Antiplatelets- no. (%) | 604 (35.24) | 429 (34.74) | 175 (36.53) | 0.485 |
| Biomarkers at admission |  |  |  |  |
| White cell count (x10^9/L) - median (IQR) | 9.90 (7.60-12.60) | 8.70 (7.00-10.50) | 12.90 (11.20-16.10) | **<0.001** |
| CRP (mg/L)- median (IQR) | 10.00 (4.00-28.00) | 6.00 (3.00-14.00) | 27.00 (15.00-55.00) | **<0.001** |
| Outcomes |  |  |  |  |
| Poor functional outcome- no. (%) | 1314 (76.66) | 911 (73.77) | 403 (84.13) | **<0.001** |
| mRS at discharge |  |  |  |  |
| Discharge mRS 0- no. (%) | 147 (8.58) | 114 (9.23) | 33 (6.89) | **<0.001** |
| Discharge mRS 1- no. (%) | 162 (9.45) | 135 (10.93) | 27 (5.64) | **<0.001** |
| Discharge mRS 2- no. (%) | 91 (5.31) | 75 (6.07) | 16 (3.34) | **<0.001** |
| Discharge mRS 3- no. (%) | 243 (14.18) | 176 (14.25) | 67 (13.99) | **<0.001** |
| Discharge mRS 4- no. (%) | 305 (17.79) | 227 (18.38) | 78 (16.28) | **<0.001** |
| Discharge mRS 5- no. (%) | 143 (8.34) | 97 (7.85) | 46 (9.6) | **<0.001** |
| Discharge mRS 6- no. (%) | 623 (36.35) | 411 (33.28) | 212 (44.26) | **<0.001** |
| Mortality |  |  |  |  |
| Death during admission- no. (%) | 623 (36.35) | 411 (33.28) | 212 (44.26) | **<0.001** |
| Death at 90 days- no. (%) | 714 (41.66) | 477 (38.62) | 237 (49.48) | **<0.001** |
| Death at 365 days- no. (%) | 834 (48.66) | 564 (45.67) | 270 (56.37) | **<0.001** |
| Length of stay |  |  |  |  |
| Days- median (IQR) | 8.00 (3.00-19.00) | 8.00 (3.00-19.00) | 8.00 (3.00-23.00) | 0.109 |
| Length of stay >14 days- no. (%) | 576 (33.61) | 395 (31.98) | 181 (37.79) | **0.022** |

Table S2.14

|  | ICH admissions | | |  |
| --- | --- | --- | --- | --- |
| Variable | Total cohort (*n*= 1714) | Non-elevated inflammatory biomarkers (n= 1228) | Elevated inflammatory biomarkers (n= 486) | *P-*value |
| Age- mean (+/- SD) | 76.05 (12.25) | 76.02 (12.28) | 76.12 (12.19) | 0.885 |
| Sex- no. male (%) | 854 (49.82) | 635 (51.71) | 219 (45.06) | **0.013** |
| Total NIHSS score- median (IQR) | 4.00 (2.00-9.00) | 4.00 (2.00-9.00) | 4.00 (2.00-9.00) | 0.130 |
| Pre-ICH mRS |  |  |  |  |
| 0- no. (%) | 1141 (66.57) | 830 (67.59) | 311 (63.99) | **0.043** |
| 1- no. (%) | 190 (11.09) | 140 (11.4) | 50 (10.29) | **0.043** |
| 2- no. (%) | 127 (7.41) | 96 (7.82) | 31 (6.38) | **0.043** |
| 3- no. (%) | 163 (9.51) | 105 (8.55) | 58 (11.93) | **0.043** |
| 4- no. (%) | 67 (3.91) | 40 (3.26) | 27 (5.56) | **0.043** |
| 5- no. (%) | 26 (1.52) | 17 (1.38) | 9 (1.85) | **0.043** |
| OCSP classification |  |  |  |  |
| TACS | 559 (32.61) | 357 (29.07) | 202 (41.56) | **<0.001** |
| PACS | 489 (28.53) | 358 (29.15) | 131 (26.95) | **<0.001** |
| LACS | 129 (7.53) | 97 (7.9) | 32 (6.58) | **<0.001** |
| POCS | 537 (31.33) | 416 (33.88) | 121 (24.9) | **<0.001** |
| Comorbidities |  |  |  |  |
| Asthma- no. (%) | 146 (8.52) | 114 (9.28) | 32 (6.58) | 0.071 |
| Atrial fibrillation- no. (%) | 383 (22.35) | 261 (21.25) | 122 (25.1) | 0.085 |
| Cerebral infarction- no. (%) | 135 (7.88) | 98 (7.98) | 37 (7.61) | 0.799 |
| Coronary heart disease- no. (%) | 327 (19.08) | 215 (17.51) | 112 (23.05) | **0.009** |
| Congestive heart failure- no. (%) | 135 (7.88) | 84 (6.84) | 51 (10.49) | **0.011** |
| Chronic kidney disease- no. (%) | 85 (4.96) | 56 (4.56) | 29 (5.97) | 0.227 |
| COPD- no. (%) | 104 (6.07) | 79 (6.43) | 25 (5.14) | 0.314 |
| Dementia- no. (%) | 71 (4.14) | 51 (4.15) | 20 (4.12) | 0.972 |
| Diabetes mellitus- no. (%) | 205 (11.96) | 146 (11.89) | 59 (12.14) | 0.885 |
| Hyperlipidemia- no. (%) | 179 (10.44) | 134 (10.91) | 45 (9.26) | 0.313 |
| Hypertension- no. (%) | 996 (58.11) | 720 (58.63) | 276 (56.79) | 0.486 |
| Liver disease- no. (%) | 26 (1.52) | 23 (1.87) | 3 (.62) | 0.055 |
| Malignancy- no. (%) | 243 (14.18) | 178 (14.5) | 65 (13.37) | 0.549 |
| Peptic ulcer disease- no. (%) | 64 (3.73) | 42 (3.42) | 22 (4.53) | 0.276 |
| Peripheral vascular disease- no. (%) | 82 (4.78) | 59 (4.8) | 23 (4.73) | 0.950 |
| Previous ICH/SAH- no. (%) | 377 (22) | 278 (22.64) | 99 (20.37) | 0.307 |
| Connective tissue disease- no. (%) | 62 (3.62) | 48 (3.91) | 14 (2.88) | 0.304 |
| Stroke-associated pneumonia- no. (%) | 194 (11.32) | 122 (9.93) | 72 (14.81) | **0.004** |
| Admission medications |  |  |  |  |
| Anticoagulants- no. (%) | 34 (1.98) | 29 (2.36) | 5 (1.03) | 0.075 |
| Antiplatelets- no. (%) | 604 (35.24) | 426 (34.69) | 178 (36.63) | 0.450 |
| Biomarkers at admission |  |  |  |  |
| White cell count (x10^9/L) - median (IQR) | 9.90 (7.60-12.60) | 8.70 (7.00-10.50) | 12.90 (11.20-16.10) | **<0.001** |
| CRP (mg/L)- median (IQR) | 10.00 (4.00-27.00) | 6.00 (4.00-14.00) | 27.00 (15.00-58.00) | **<0.001** |
| Outcomes |  |  |  |  |
| Poor functional outcome- no. (%) | 1312 (76.55) | 912 (74.27) | 400 (82.3) | **<0.001** |
| mRS at discharge |  |  |  |  |
| Discharge mRS 0- no. (%) | 156 (9.1) | 119 (9.69) | 37 (7.61) | **<0.001** |
| Discharge mRS 1- no. (%) | 148 (8.63) | 116 (9.45) | 32 (6.58) | **<0.001** |
| Discharge mRS 2- no. (%) | 98 (5.72) | 81 (6.6) | 17 (3.5) | **<0.001** |
| Discharge mRS 3- no. (%) | 251 (14.64) | 186 (15.15) | 65 (13.37) | **<0.001** |
| Discharge mRS 4- no. (%) | 292 (17.04) | 216 (17.59) | 76 (15.64) | **<0.001** |
| Discharge mRS 5- no. (%) | 146 (8.52) | 100 (8.14) | 46 (9.47) | **<0.001** |
| Discharge mRS 6- no. (%) | 623 (36.35) | 410 (33.39) | 213 (43.83) | **<0.001** |
| Mortality |  |  |  |  |
| Death during admission- no. (%) | 623 (36.35) | 410 (33.39) | 213 (43.83) | **<0.001** |
| Death at 90 days- no. (%) | 714 (41.66) | 473 (38.52) | 241 (49.59) | **<0.001** |
| Death at 365 days- no. (%) | 834 (48.66) | 559 (45.52) | 275 (56.58) | **<0.001** |
| Length of stay |  |  |  |  |
| Days- median (IQR) | 8.00 (3.00-19.00) | 8.00 (3.00-19.00) | 8.00 (3.00-22.00) | 0.267 |
| Length of stay >14 days- no. (%) | 576 (33.61) | 395 (32.17) | 181 (37.24) | **0.045** |

Table S2.15

|  | ICH admissions | | |  |
| --- | --- | --- | --- | --- |
| Variable | Total cohort (*n*= 1714) | Non-elevated inflammatory biomarkers (n= 1234) | Elevated inflammatory biomarkers (n= 480) | *P-*value |
| Age- mean (+/- SD) | 76.05 (12.25) | 75.79 (12.46) | 76.73 (11.68) | 0.151 |
| Sex- no. male (%) | 854 (49.82) | 640 (51.86) | 214 (44.58) | **0.007** |
| Total NIHSS score- median (IQR) | 6.00 (2.00-9.00) | 6.00 (2.00-9.00) | 6.00 (2.00-9.00) | 0.182 |
| Pre-ICH mRS |  |  |  |  |
| 0- no. (%) | 1136 (66.28) | 838 (67.91) | 298 (62.08) | **0.003** |
| 1- no. (%) | 195 (11.38) | 140 (11.35) | 55 (11.46) | **0.003** |
| 2- no. (%) | 129 (7.53) | 98 (7.94) | 31 (6.46) | **0.003** |
| 3- no. (%) | 160 (9.33) | 104 (8.43) | 56 (11.67) | **0.003** |
| 4- no. (%) | 67 (3.91) | 36 (2.92) | 31 (6.46) | **0.003** |
| 5- no. (%) | 27 (1.58) | 18 (1.46) | 9 (1.88) | **0.003** |
| OCSP classification |  |  |  |  |
| TACS | 555 (32.38) | 356 (28.85) | 199 (41.46) | **<0.001** |
| PACS | 493 (28.76) | 377 (30.55) | 116 (24.17) | **<0.001** |
| LACS | 138 (8.05) | 107 (8.67) | 31 (6.46) | **<0.001** |
| POCS | 528 (30.81) | 394 (31.93) | 134 (27.92) | **<0.001** |
| Comorbidities |  |  |  |  |
| Asthma- no. (%) | 146 (8.52) | 112 (9.08) | 34 (7.08) | 0.184 |
| Atrial fibrillation- no. (%) | 383 (22.35) | 261 (21.15) | 122 (25.42) | 0.057 |
| Cerebral infarction- no. (%) | 135 (7.88) | 98 (7.94) | 37 (7.71) | 0.872 |
| Coronary heart disease- no. (%) | 327 (19.08) | 221 (17.91) | 106 (22.08) | **0.048** |
| Congestive heart failure- no. (%) | 135 (7.88) | 85 (6.89) | 50 (10.42) | **0.015** |
| Chronic kidney disease- no. (%) | 85 (4.96) | 57 (4.62) | 28 (5.83) | 0.298 |
| COPD- no. (%) | 104 (6.07) | 80 (6.48) | 24 (5) | 0.248 |
| Dementia- no. (%) | 71 (4.14) | 51 (4.13) | 20 (4.17) | 0.975 |
| Diabetes mellitus- no. (%) | 205 (11.96) | 147 (11.91) | 58 (12.08) | 0.922 |
| Hyperlipidemia- no. (%) | 179 (10.44) | 133 (10.78) | 46 (9.58) | 0.468 |
| Hypertension- no. (%) | 996 (58.11) | 719 (58.27) | 277 (57.71) | 0.834 |
| Liver disease- no. (%) | 26 (1.52) | 24 (1.94) | 2 (.42) | **0.020** |
| Malignancy- no. (%) | 243 (14.18) | 177 (14.34) | 66 (13.75) | 0.752 |
| Peptic ulcer disease- no. (%) | 64 (3.73) | 41 (3.32) | 23 (4.79) | 0.150 |
| Peripheral vascular disease- no. (%) | 82 (4.78) | 61 (4.94) | 21 (4.38) | 0.621 |
| Previous ICH/SAH- no. (%) | 377 (22) | 278 (22.53) | 99 (20.63) | 0.393 |
| Connective tissue disease- no. (%) | 62 (3.62) | 49 (3.97) | 13 (2.71) | 0.209 |
| Stroke-associated pneumonia- no. (%) | 194 (11.32) | 119 (9.64) | 75 (15.63) | **<0.001** |
| Admission medications |  |  |  |  |
| Anticoagulants- no. (%) | 34 (1.98) | 29 (2.35) | 5 (1.04) | 0.081 |
| Antiplatelets- no. (%) | 604 (35.24) | 426 (34.52) | 178 (37.08) | 0.319 |
| Biomarkers at admission |  |  |  |  |
| White cell count (x10^9/L) - median (IQR) | 9.90 (7.60-12.60) | 8.70 (7.00-10.50) | 12.90 (11.30-16.15) | **<0.001** |
| CRP (mg/L)- median (IQR) | 10.00 (4.00-28.00) | 6.00 (3.00-14.00) | 27.00 (14.00-58.00) | **<0.001** |
| Outcomes |  |  |  |  |
| Poor functional outcome- no. (%) | 1311 (76.49) | 913 (73.99) | 398 (82.92) | **<0.001** |
| mRS at discharge |  |  |  |  |
| Discharge mRS 0- no. (%) | 143 (8.34) | 117 (9.48) | 26 (5.42) | **<0.001** |
| Discharge mRS 1- no. (%) | 161 (9.39) | 123 (9.97) | 38 (7.92) | **<0.001** |
| Discharge mRS 2- no. (%) | 99 (5.78) | 81 (6.56) | 18 (3.75) | **<0.001** |
| Discharge mRS 3- no. (%) | 240 (14) | 181 (14.67) | 59 (12.29) | **<0.001** |
| Discharge mRS 4- no. (%) | 315 (18.38) | 233 (18.88) | 82 (17.08) | **<0.001** |
| Discharge mRS 5- no. (%) | 133 (7.76) | 95 (7.7) | 38 (7.92) | **<0.001** |
| Discharge mRS 6- no. (%) | 623 (36.35) | 404 (32.74) | 219 (45.63) | **<0.001** |
| Mortality |  |  |  |  |
| Death during admission- no. (%) | 623 (36.35) | 404 (32.74) | 219 (45.63) | **<0.001** |
| Death at 90 days- no. (%) | 714 (41.66) | 472 (38.25) | 242 (50.42) | **<0.001** |
| Death at 365 days- no. (%) | 834 (48.66) | 559 (45.3) | 275 (57.29) | **<0.001** |
| Length of stay |  |  |  |  |
| Days- median (IQR) | 8.00 (3.00-19.00) | 8.00 (3.00-19.00) | 8.00 (3.00-23.50) | 0.187 |
| Length of stay >14 days- no. (%) | 576 (33.61) | 395 (32.01) | 181 (37.71) | **0.025** |

Table S2.16

|  | ICH admissions | | |  |
| --- | --- | --- | --- | --- |
| Variable | Total cohort (*n*= 1714) | Non-elevated inflammatory biomarkers (n= 1237) | Elevated inflammatory biomarkers (n= 477) | *P-*value |
| Age- mean (+/- SD) | 76.05 (12.25) | 75.94 (12.37) | 76.35 (11.92) | 0.529 |
| Sex- no. male (%) | 854 (49.82) | 638 (51.58) | 216 (45.28) | **0.020** |
| Total NIHSS score- median (IQR) | 4.00 (2.00-10.00) | 4.00 (2.00-9.00) | 5.00 (2.00-12.00) | **0.012** |
| Pre-ICH mRS |  |  |  |  |
| 0- no. (%) | 1137 (66.34) | 833 (67.34) | 304 (63.73) | **0.010** |
| 1- no. (%) | 191 (11.14) | 139 (11.24) | 52 (10.9) | **0.010** |
| 2- no. (%) | 125 (7.29) | 100 (8.08) | 25 (5.24) | **0.010** |
| 3- no. (%) | 167 (9.74) | 106 (8.57) | 61 (12.79) | **0.010** |
| 4- no. (%) | 68 (3.97) | 42 (3.4) | 26 (5.45) | **0.010** |
| 5- no. (%) | 26 (1.52) | 17 (1.37) | 9 (1.89) | **0.010** |
| OCSP classification |  |  |  |  |
| TACS | 558 (32.56) | 364 (29.43) | 194 (40.67) | **<0.001** |
| PACS | 516 (30.11) | 389 (31.45) | 127 (26.62) | **<0.001** |
| LACS | 136 (7.93) | 105 (8.49) | 31 (6.5) | **<0.001** |
| POCS | 504 (29.4) | 379 (30.64) | 125 (26.21) | **<0.001** |
| Comorbidities |  |  |  |  |
| Asthma- no. (%) | 146 (8.52) | 115 (9.3) | 31 (6.5) | 0.063 |
| Atrial fibrillation- no. (%) | 383 (22.35) | 262 (21.18) | 121 (25.37) | 0.062 |
| Cerebral infarction- no. (%) | 135 (7.88) | 98 (7.92) | 37 (7.76) | 0.909 |
| Coronary heart disease- no. (%) | 327 (19.08) | 221 (17.87) | 106 (22.22) | **0.040** |
| Congestive heart failure- no. (%) | 135 (7.88) | 89 (7.19) | 46 (9.64) | 0.092 |
| Chronic kidney disease- no. (%) | 85 (4.96) | 57 (4.61) | 28 (5.87) | 0.281 |
| COPD- no. (%) | 104 (6.07) | 80 (6.47) | 24 (5.03) | 0.264 |
| Dementia- no. (%) | 71 (4.14) | 50 (4.04) | 21 (4.4) | 0.737 |
| Diabetes mellitus- no. (%) | 205 (11.96) | 147 (11.88) | 58 (12.16) | 0.875 |
| Hyperlipidemia- no. (%) | 179 (10.44) | 133 (10.75) | 46 (9.64) | 0.501 |
| Hypertension- no. (%) | 996 (58.11) | 726 (58.69) | 270 (56.6) | 0.433 |
| Liver disease- no. (%) | 26 (1.52) | 23 (1.86) | 3 (.63) | 0.062 |
| Malignancy- no. (%) | 243 (14.18) | 177 (14.31) | 66 (13.84) | 0.802 |
| Peptic ulcer disease- no. (%) | 64 (3.73) | 43 (3.48) | 21 (4.4) | 0.365 |
| Peripheral vascular disease- no. (%) | 82 (4.78) | 61 (4.93) | 21 (4.4) | 0.646 |
| Previous ICH/SAH- no. (%) | 377 (22) | 280 (22.64) | 97 (20.34) | 0.303 |
| Connective tissue disease- no. (%) | 62 (3.62) | 49 (3.96) | 13 (2.73) | 0.219 |
| Stroke-associated pneumonia- no. (%) | 194 (11.32) | 119 (9.62) | 75 (15.72) | **<0.001** |
| Admission medications |  |  |  |  |
| Anticoagulants- no. (%) | 34 (1.98) | 29 (2.34) | 5 (1.05) | 0.085 |
| Antiplatelets- no. (%) | 604 (35.24) | 428 (34.6) | 176 (36.9) | 0.372 |
| Biomarkers at admission |  |  |  |  |
| White cell count (x10^9/L) - median (IQR) | 9.90 (7.60-12.60) | 8.70 (7.00-10.50) | 12.80 (11.20-15.90) | **<0.001** |
| CRP (mg/L)- median (IQR) | 10.00 (4.00-28.00) | 6.00 (4.00-14.00) | 26.00 (15.00-55.00) | **<0.001** |
| Outcomes |  |  |  |  |
| Poor functional outcome- no. (%) | 1312 (76.55) | 915 (73.97) | 397 (83.23) | **<0.001** |
| mRS at discharge |  |  |  |  |
| Discharge mRS 0- no. (%) | 139 (8.11) | 107 (8.65) | 32 (6.71) | **<0.001** |
| Discharge mRS 1- no. (%) | 168 (9.8) | 136 (10.99) | 32 (6.71) | **<0.001** |
| Discharge mRS 2- no. (%) | 95 (5.54) | 79 (6.39) | 16 (3.35) | **<0.001** |
| Discharge mRS 3- no. (%) | 239 (13.94) | 175 (14.15) | 64 (13.42) | **<0.001** |
| Discharge mRS 4- no. (%) | 308 (17.97) | 232 (18.76) | 76 (15.93) | **<0.001** |
| Discharge mRS 5- no. (%) | 142 (8.28) | 96 (7.76) | 46 (9.64) | **<0.001** |
| Discharge mRS 6- no. (%) | 623 (36.35) | 412 (33.31) | 211 (44.23) | **<0.001** |
| Mortality |  |  |  |  |
| Death during admission- no. (%) | 623 (36.35) | 412 (33.31) | 211 (44.23) | **<0.001** |
| Death at 90 days- no. (%) | 714 (41.66) | 478 (38.64) | 236 (49.48) | **<0.001** |
| Death at 365 days- no. (%) | 834 (48.66) | 565 (45.68) | 269 (56.39) | **<0.001** |
| Length of stay |  |  |  |  |
| Days- median (IQR) | 8.00 (3.00-19.00) | 8.00 (3.00-19.00) | 8.00 (3.00-23.00) | 0.105 |
| Length of stay >14 days- no. (%) | 576 (33.61) | 396 (32.01) | 180 (37.74) | **0.025** |

Table S2.17

|  | ICH admissions | | |  |
| --- | --- | --- | --- | --- |
| Variable | Total cohort (*n*= 1714) | Non-elevated inflammatory biomarkers (n= 1231) | Elevated inflammatory biomarkers (n= 483) | *P-*value |
| Age- mean (+/- SD) | 76.05 (12.25) | 75.99 (12.21) | 76.21 (12.35) | 0.737 |
| Sex- no. male (%) | 854 (49.82) | 639 (51.91) | 215 (44.51) | **0.006** |
| Total NIHSS score- median (IQR) | 2.00 (0.00-6.00) | 2.00 (0.00-6.00) | 2.00 (0.00-5.00) | 0.766 |
| Pre-ICH mRS |  |  |  |  |
| 0- no. (%) | 1138 (66.39) | 835 (67.83) | 303 (62.73) | **0.016** |
| 1- no. (%) | 200 (11.67) | 141 (11.45) | 59 (12.22) | **0.016** |
| 2- no. (%) | 126 (7.35) | 98 (7.96) | 28 (5.8) | **0.016** |
| 3- no. (%) | 159 (9.28) | 100 (8.12) | 59 (12.22) | **0.016** |
| 4- no. (%) | 65 (3.79) | 40 (3.25) | 25 (5.18) | **0.016** |
| 5- no. (%) | 26 (1.52) | 17 (1.38) | 9 (1.86) | **0.016** |
| OCSP classification |  |  |  |  |
| TACS | 567 (33.08) | 363 (29.49) | 204 (42.24) | **<0.001** |
| PACS | 488 (28.47) | 365 (29.65) | 123 (25.47) | **<0.001** |
| LACS | 134 (7.82) | 100 (8.12) | 34 (7.04) | **<0.001** |
| POCS | 525 (30.63) | 403 (32.74) | 122 (25.26) | **<0.001** |
| Comorbidities |  |  |  |  |
| Asthma- no. (%) | 146 (8.52) | 115 (9.34) | 31 (6.42) | 0.051 |
| Atrial fibrillation- no. (%) | 383 (22.35) | 262 (21.28) | 121 (25.05) | 0.092 |
| Cerebral infarction- no. (%) | 135 (7.88) | 98 (7.96) | 37 (7.66) | 0.835 |
| Coronary heart disease- no. (%) | 327 (19.08) | 218 (17.71) | 109 (22.57) | **0.021** |
| Congestive heart failure- no. (%) | 135 (7.88) | 83 (6.74) | 52 (10.77) | **0.005** |
| Chronic kidney disease- no. (%) | 85 (4.96) | 57 (4.63) | 28 (5.8) | 0.317 |
| COPD- no. (%) | 104 (6.07) | 80 (6.5) | 24 (4.97) | 0.233 |
| Dementia- no. (%) | 71 (4.14) | 51 (4.14) | 20 (4.14) | 0.998 |
| Diabetes mellitus- no. (%) | 205 (11.96) | 146 (11.86) | 59 (12.22) | 0.839 |
| Hyperlipidemia- no. (%) | 179 (10.44) | 134 (10.89) | 45 (9.32) | 0.339 |
| Hypertension- no. (%) | 996 (58.11) | 719 (58.41) | 277 (57.35) | 0.690 |
| Liver disease- no. (%) | 26 (1.52) | 24 (1.95) | 2 (.41) | **0.019** |
| Malignancy- no. (%) | 243 (14.18) | 174 (14.13) | 69 (14.29) | 0.936 |
| Peptic ulcer disease- no. (%) | 64 (3.73) | 42 (3.41) | 22 (4.55) | 0.261 |
| Peripheral vascular disease- no. (%) | 82 (4.78) | 59 (4.79) | 23 (4.76) | 0.978 |
| Previous ICH/SAH- no. (%) | 377 (22) | 275 (22.34) | 102 (21.12) | 0.583 |
| Connective tissue disease- no. (%) | 62 (3.62) | 49 (3.98) | 13 (2.69) | 0.199 |
| Stroke-associated pneumonia- no. (%) | 194 (11.32) | 121 (9.83) | 73 (15.11) | **0.002** |
| Admission medications |  |  |  |  |
| Anticoagulants- no. (%) | 34 (1.98) | 29 (2.36) | 5 (1.04) | 0.078 |
| Antiplatelets- no. (%) | 604 (35.24) | 426 (34.61) | 178 (36.85) | 0.381 |
| Biomarkers at admission |  |  |  |  |
| White cell count (x10^9/L) - median (IQR) | 9.90 (7.60-12.60) | 8.70 (7.00-10.40) | 12.90 (11.30-16.10) | **<0.001** |
| CRP (mg/L)- median (IQR) | 10.00 (4.00-28.00) | 6.00 (3.00-14.00) | 27.00 (14.00-55.00) | **<0.001** |
| Outcomes |  |  |  |  |
| Poor functional outcome- no. (%) | 1317 (76.84) | 913 (74.17) | 404 (83.64) | **<0.001** |
| mRS at discharge |  |  |  |  |
| Discharge mRS 0- no. (%) | 154 (8.98) | 123 (9.99) | 31 (6.42) | **<0.001** |
| Discharge mRS 1- no. (%) | 145 (8.46) | 118 (9.59) | 27 (5.59) | **<0.001** |
| Discharge mRS 2- no. (%) | 98 (5.72) | 77 (6.26) | 21 (4.35) | **<0.001** |
| Discharge mRS 3- no. (%) | 242 (14.12) | 169 (13.73) | 73 (15.11) | **<0.001** |
| Discharge mRS 4- no. (%) | 301 (17.56) | 231 (18.77) | 70 (14.49) | **<0.001** |
| Discharge mRS 5- no. (%) | 151 (8.81) | 103 (8.37) | 48 (9.94) | **<0.001** |
| Discharge mRS 6- no. (%) | 623 (36.35) | 410 (33.31) | 213 (44.1) | **<0.001** |
| Mortality |  |  |  |  |
| Death during admission- no. (%) | 623 (36.35) | 410 (33.31) | 213 (44.1) | **<0.001** |
| Death at 90 days- no. (%) | 714 (41.66) | 474 (38.51) | 240 (49.69) | **<0.001** |
| Death at 365 days- no. (%) | 834 (48.66) | 561 (45.57) | 273 (56.52) | **<0.001** |
| Length of stay |  |  |  |  |
| Days- median (IQR) | 8.00 (3.00-19.00) | 8.00 (3.00-19.00) | 8.00 (3.00-22.00) | 0.256 |
| Length of stay >14 days- no. (%) | 576 (33.61) | 397 (32.25) | 179 (37.06) | 0.058 |

Table S2.18

|  | ICH admissions | | |  |
| --- | --- | --- | --- | --- |
| Variable | Total cohort (*n*= 1714) | Non-elevated inflammatory biomarkers (n= 1236 | Elevated inflammatory biomarkers (n= 478) | *P-*value |
| Age- mean (+/- SD) | 76.05 (12.25) | 75.85 (12.44) | 76.56 (11.73) | 0.284 |
| Sex- no. male (%) | 854 (49.82) | 640 (51.78) | 214 (44.77) | **0.009** |
| Total NIHSS score- median (IQR) | 4.00 (2.00-10.00) | 4.00 (2.00-10.00) | 4.50 (2.00-10.00) | 0.786 |
| Pre-ICH mRS |  |  |  |  |
| 0- no. (%) | 1143 (66.69) | 839 (67.88) | 304 (63.6) | **0.008** |
| 1- no. (%) | 188 (10.97) | 138 (11.17) | 50 (10.46) | **0.008** |
| 2- no. (%) | 129 (7.53) | 100 (8.09) | 29 (6.07) | **0.008** |
| 3- no. (%) | 160 (9.33) | 104 (8.41) | 56 (11.72) | **0.008** |
| 4- no. (%) | 66 (3.85) | 38 (3.07) | 28 (5.86) | **0.008** |
| 5- no. (%) | 28 (1.63) | 17 (1.38) | 11 (2.3) | **0.008** |
| OCSP classification |  |  |  |  |
| TACS | 554 (32.32) | 361 (29.21) | 193 (40.38) | **<0.001** |
| PACS | 498 (29.05) | 370 (29.94) | 128 (26.78) | **<0.001** |
| LACS | 142 (8.28) | 105 (8.5) | 37 (7.74) | **<0.001** |
| POCS | 520 (30.34) | 400 (32.36) | 120 (25.1) | **<0.001** |
| Comorbidities |  |  |  |  |
| Asthma- no. (%) | 146 (8.52) | 115 (9.3) | 31 (6.49) | 0.061 |
| Atrial fibrillation- no. (%) | 383 (22.35) | 261 (21.12) | 122 (25.52) | 0.050 |
| Cerebral infarction- no. (%) | 135 (7.88) | 99 (8.01) | 36 (7.53) | 0.742 |
| Coronary heart disease- no. (%) | 327 (19.08) | 218 (17.64) | 109 (22.8) | **0.015** |
| Congestive heart failure- no. (%) | 135 (7.88) | 85 (6.88) | 50 (10.46) | **0.014** |
| Chronic kidney disease- no. (%) | 85 (4.96) | 57 (4.61) | 28 (5.86) | 0.287 |
| COPD- no. (%) | 104 (6.07) | 81 (6.55) | 23 (4.81) | 0.176 |
| Dementia- no. (%) | 71 (4.14) | 51 (4.13) | 20 (4.18) | 0.957 |
| Diabetes mellitus- no. (%) | 205 (11.96) | 144 (11.65) | 61 (12.76) | 0.525 |
| Hyperlipidemia- no. (%) | 179 (10.44) | 134 (10.84) | 45 (9.41) | 0.386 |
| Hypertension- no. (%) | 996 (58.11) | 721 (58.33) | 275 (57.53) | 0.763 |
| Liver disease- no. (%) | 26 (1.52) | 24 (1.94) | 2 (.42) | **0.021** |
| Malignancy- no. (%) | 243 (14.18) | 176 (14.24) | 67 (14.02) | 0.906 |
| Peptic ulcer disease- no. (%) | 64 (3.73) | 43 (3.48) | 21 (4.39) | 0.371 |
| Peripheral vascular disease- no. (%) | 82 (4.78) | 59 (4.77) | 23 (4.81) | 0.973 |
| Previous ICH/SAH- no. (%) | 377 (22) | 276 (22.33) | 101 (21.13) | 0.591 |
| Connective tissue disease- no. (%) | 62 (3.62) | 48 (3.88) | 14 (2.93) | 0.343 |
| Stroke-associated pneumonia- no. (%) | 194 (11.32) | 119 (9.63) | 75 (15.69) | **<0.001** |
| Admission medications |  |  |  |  |
| Anticoagulants- no. (%) | 34 (1.98) | 29 (2.35) | 5 (1.05) | 0.083 |
| Antiplatelets- no. (%) | 604 (35.24) | 427 (34.55) | 177 (37.03) | 0.335 |
| Biomarkers at admission |  |  |  |  |
| White cell count (x10^9/L) - median (IQR) | 9.90 (7.60-12.60) | 8.70 (7.00-10.50) | 13.05 (11.30-16.20) | **<0.001** |
| CRP (mg/L)- median (IQR) | 10.00 (4.00-27.00) | 6.00 (3.00-13.00) | 27.00 (15.00-59.00) | **<0.001** |
| Outcomes |  |  |  |  |
| Poor functional outcome- no. (%) | 1323 (77.19) | 917 (74.19) | 406 (84.94) | **<0.001** |
| mRS at discharge |  |  |  |  |
| Discharge mRS 0- no. (%) | 145 (8.46) | 117 (9.47) | 28 (5.86) | **<0.001** |
| Discharge mRS 1- no. (%) | 156 (9.1) | 125 (10.11) | 31 (6.49) | **<0.001** |
| Discharge mRS 2- no. (%) | 90 (5.25) | 77 (6.23) | 13 (2.72) | **<0.001** |
| Discharge mRS 3- no. (%) | 248 (14.47) | 179 (14.48) | 69 (14.44) | **<0.001** |
| Discharge mRS 4- no. (%) | 307 (17.91) | 226 (18.28) | 81 (16.95) | **<0.001** |
| Discharge mRS 5- no. (%) | 145 (8.46) | 104 (8.41) | 41 (8.58) | **<0.001** |
| Discharge mRS 6- no. (%) | 623 (36.35) | 408 (33.01) | 215 (44.98) | **<0.001** |
| Mortality |  |  |  |  |
| Death during admission- no. (%) | 623 (36.35) | 408 (33.01) | 215 (44.98) | **<0.001** |
| Death at 90 days- no. (%) | 714 (41.66) | 473 (38.27) | 241 (50.42) | **<0.001** |
| Death at 365 days- no. (%) | 834 (48.66) | 560 (45.31) | 274 (57.32) | **<0.001** |
| Length of stay |  |  |  |  |
| Days- median (IQR) | 8.00 (3.00-19.00) | 8.00 (3.00-19.00) | 8.00 (3.00-23.00) | 0.081 |
| Length of stay >14 days- no. (%) | 576 (33.61) | 396 (32.04) | 180 (37.66) | **0.027** |

Table S2.19

| Variable | Total cohort (*n*= 1714) | Non-elevated inflammatory biomarkers (n= 1236) | Elevated inflammatory biomarkers (n= 478) | *P-*value |
| --- | --- | --- | --- | --- |
| Age- mean (+/- SD) | 76.05 (12.25) | 75.91 (12.36) | 76.42 (11.94) | 0.433 |
| Sex- no. male (%) | 854 (49.82) | 636 (51.46) | 218 (45.61) | **0.030** |
| Total NIHSS score- median (IQR) | 3.00 (1.00-8.00) | 3.00 (1.00-7.00) | 3.00 (1.00-9.00) | 0.624 |
| Pre-ICH mRS |  |  |  |  |
| 0- no. (%) | 1138 (66.39) | 839 (67.88) | 299 (62.55) | **0.006** |
| 1- no. (%) | 196 (11.44) | 139 (11.25) | 57 (11.92) | **0.006** |
| 2- no. (%) | 125 (7.29) | 98 (7.93) | 27 (5.65) | **0.006** |
| 3- no. (%) | 164 (9.57) | 105 (8.5) | 59 (12.34) | **0.006** |
| 4- no. (%) | 67 (3.91) | 39 (3.16) | 28 (5.86) | **0.006** |
| 5- no. (%) | 24 (1.4) | 16 (1.29) | 8 (1.67) | **0.006** |
| OCSP classification |  |  |  |  |
| TACS | 579 (33.78) | 381 (30.83) | 198 (41.42) | **<0.001** |
| PACS | 487 (28.41) | 365 (29.53) | 122 (25.52) | **<0.001** |
| LACS | 134 (7.82) | 100 (8.09) | 34 (7.11) | **<0.001** |
| POCS | 514 (29.99) | 390 (31.55) | 124 (25.94) | **<0.001** |
| Comorbidities |  |  |  |  |
| Asthma- no. (%) | 146 (8.52) | 113 (9.14) | 33 (6.9) | 0.137 |
| Atrial fibrillation- no. (%) | 383 (22.35) | 265 (21.44) | 118 (24.69) | 0.148 |
| Cerebral infarction- no. (%) | 135 (7.88) | 98 (7.93) | 37 (7.74) | 0.897 |
| Coronary heart disease- no. (%) | 327 (19.08) | 218 (17.64) | 109 (22.8) | **0.015** |
| Congestive heart failure- no. (%) | 135 (7.88) | 86 (6.96) | 49 (10.25) | **0.023** |
| Chronic kidney disease- no. (%) | 85 (4.96) | 56 (4.53) | 29 (6.07) | 0.189 |
| COPD- no. (%) | 104 (6.07) | 79 (6.39) | 25 (5.23) | 0.366 |
| Dementia- no. (%) | 71 (4.14) | 50 (4.05) | 21 (4.39) | 0.746 |
| Diabetes mellitus- no. (%) | 205 (11.96) | 150 (12.14) | 55 (11.51) | 0.719 |
| Hyperlipidemia- no. (%) | 179 (10.44) | 133 (10.76) | 46 (9.62) | 0.490 |
| Hypertension- no. (%) | 996 (58.11) | 722 (58.41) | 274 (57.32) | 0.681 |
| Liver disease- no. (%) | 26 (1.52) | 24 (1.94) | 2 (.42) | **0.021** |
| Malignancy- no. (%) | 243 (14.18) | 178 (14.4) | 65 (13.6) | 0.669 |
| Peptic ulcer disease- no. (%) | 64 (3.73) | 43 (3.48) | 21 (4.39) | 0.371 |
| Peripheral vascular disease- no. (%) | 82 (4.78) | 60 (4.85) | 22 (4.6) | 0.827 |
| Previous ICH/SAH- no. (%) | 377 (22) | 279 (22.57) | 98 (20.5) | 0.353 |
| Connective tissue disease- no. (%) | 62 (3.62) | 48 (3.88) | 14 (2.93) | 0.343 |
| Stroke-associated pneumonia- no. (%) | 194 (11.32) | 119 (9.63) | 75 (15.69) | **<0.001** |
| Admission medications |  |  |  |  |
| Anticoagulants- no. (%) | 34 (1.98) | 29 (2.35) | 5 (1.05) | 0.083 |
| Antiplatelets- no. (%) | 604 (35.24) | 422 (34.14) | 182 (38.08) | 0.126 |
| Biomarkers at admission |  |  |  |  |
| White cell count (x10^9/L) - median (IQR) | 9.90 (7.60-12.60) | 8.70 (7.00-10.50) | 12.90 (11.30-16.20) | **<0.001** |
| CRP (mg/L)- median (IQR) | 10.00 (4.00-27.00) | 6.00 (3.00-14.00) | 26.00 (14.00-55.00) | **<0.001** |
| Outcomes |  |  |  |  |
| Poor functional outcome- no. (%) | 1312 (76.55) | 917 (74.19) | 395 (82.64) | **<0.001** |
| mRS at discharge |  |  |  |  |
| Discharge mRS 0- no. (%) | 154 (8.98) | 124 (10.03) | 30 (6.28) | **<0.001** |
| Discharge mRS 1- no. (%) | 159 (9.28) | 125 (10.11) | 34 (7.11) | **<0.001** |
| Discharge mRS 2- no. (%) | 89 (5.19) | 70 (5.66) | 19 (3.97) | **<0.001** |
| Discharge mRS 3- no. (%) | 250 (14.59) | 187 (15.13) | 63 (13.18) | **<0.001** |
| Discharge mRS 4- no. (%) | 306 (17.85) | 231 (18.69) | 75 (15.69) | **<0.001** |
| Discharge mRS 5- no. (%) | 133 (7.76) | 89 (7.2) | 44 (9.21) | **<0.001** |
| Discharge mRS 6- no. (%) | 623 (36.35) | 410 (33.17) | 213 (44.56) | **<0.001** |
| Mortality |  |  |  |  |
| Death during admission- no. (%) | 623 (36.35) | 410 (33.17) | 213 (44.56) | **<0.001** |
| Death at 90 days- no. (%) | 714 (41.66) | 476 (38.51) | 238 (49.79) | **<0.001** |
| Death at 365 days- no. (%) | 834 (48.66) | 564 (45.63) | 270 (56.49) | **<0.001** |
| Length of stay |  |  |  |  |
| Days- median (IQR) | 8.00 (3.00-19.00) | 8.00 (3.00-19.00) | 8.00 (3.00-23.00) | 0.149 |
| Length of stay >14 days- no. (%) | 576 (33.61) | 395 (31.96) | 181 (37.87) | **0.020** |

Table S2.20

|  | ICH admissions | | |  |
| --- | --- | --- | --- | --- |
| Variable | Total cohort (*n*= 1714) | Non-elevated inflammatory biomarkers (n= 1225) | Elevated inflammatory biomarkers (n= 489) | *P-*value |
| Age- mean (+/- SD) | 76.05 (12.25) | 75.88 (12.48) | 76.47 (11.65) | 0.369 |
| Sex- no. male (%) | 854 (49.82) | 636 (51.92) | 218 (44.58) | **0.006** |
| Total NIHSS score- median (IQR) | 3.00 (1.00-7.00) | 3.00 (1.00-7.00) | 3.00 (1.00-8.00) | 0.340 |
| Pre-ICH mRS |  |  |  |  |
| 0- no. (%) | 1137 (66.34) | 828 (67.59) | 309 (63.19) | **0.003** |
| 1- no. (%) | 188 (10.97) | 133 (10.86) | 55 (11.25) | **0.003** |
| 2- no. (%) | 128 (7.47) | 101 (8.24) | 27 (5.52) | **0.003** |
| 3- no. (%) | 165 (9.63) | 108 (8.82) | 57 (11.66) | **0.003** |
| 4- no. (%) | 70 (4.08) | 38 (3.1) | 32 (6.54) | **0.003** |
| 5- no. (%) | 26 (1.52) | 17 (1.39) | 9 (1.84) | **0.003** |
| OCSP classification |  |  |  |  |
| TACS | 566 (33.02) | 358 (29.22) | 208 (42.54) | **<0.001** |
| PACS | 476 (27.77) | 359 (29.31) | 117 (23.93) | **<0.001** |
| LACS | 134 (7.82) | 107 (8.73) | 27 (5.52) | **<0.001** |
| POCS | 538 (31.39) | 401 (32.73) | 137 (28.02) | **<0.001** |
| Comorbidities |  |  |  |  |
| Asthma- no. (%) | 146 (8.52) | 112 (9.14) | 34 (6.95) | 0.142 |
| Atrial fibrillation- no. (%) | 383 (22.35) | 260 (21.22) | 123 (25.15) | 0.078 |
| Cerebral infarction- no. (%) | 135 (7.88) | 98 (8) | 37 (7.57) | 0.764 |
| Coronary heart disease- no. (%) | 327 (19.08) | 217 (17.71) | 110 (22.49) | **0.023** |
| Congestive heart failure- no. (%) | 135 (7.88) | 85 (6.94) | 50 (10.22) | **0.023** |
| Chronic kidney disease- no. (%) | 85 (4.96) | 57 (4.65) | 28 (5.73) | 0.356 |
| COPD- no. (%) | 104 (6.07) | 81 (6.61) | 23 (4.7) | 0.135 |
| Dementia- no. (%) | 71 (4.14) | 51 (4.16) | 20 (4.09) | 0.945 |
| Diabetes mellitus- no. (%) | 205 (11.96) | 147 (12) | 58 (11.86) | 0.936 |
| Hyperlipidemia- no. (%) | 179 (10.44) | 134 (10.94) | 45 (9.2) | 0.289 |
| Hypertension- no. (%) | 996 (58.11) | 721 (58.86) | 275 (56.24) | 0.321 |
| Liver disease- no. (%) | 26 (1.52) | 23 (1.88) | 3 (.61) | 0.053 |
| Malignancy- no. (%) | 243 (14.18) | 175 (14.29) | 68 (13.91) | 0.839 |
| Peptic ulcer disease- no. (%) | 64 (3.73) | 42 (3.43) | 22 (4.5) | 0.291 |
| Peripheral vascular disease- no. (%) | 82 (4.78) | 61 (4.98) | 21 (4.29) | 0.548 |
| Previous ICH/SAH- no. (%) | 377 (22) | 278 (22.69) | 99 (20.25) | 0.269 |
| Connective tissue disease- no. (%) | 62 (3.62) | 48 (3.92) | 14 (2.86) | 0.291 |
| Stroke-associated pneumonia- no. (%) | 194 (11.32) | 121 (9.88) | 73 (14.93) | **0.003** |
| Admission medications |  |  |  |  |
| Anticoagulants- no. (%) | 34 (1.98) | 29 (2.37) | 5 (1.02) | 0.071 |
| Antiplatelets- no. (%) | 604 (35.24) | 427 (34.86) | 177 (36.2) | 0.600 |
| Biomarkers at admission |  |  |  |  |
| White cell count (x10^9/L) - median (IQR) | 9.90 (7.60-12.60) | 8.60 (7.00-10.40) | 12.90 (11.30-16.10) | **<0.001** |
| CRP (mg/L)- median (IQR) | 10.00 (4.00-27.00) | 6.00 (3.00-14.00) | 26.00 (14.00-55.00) | **<0.001** |
| Outcomes |  |  |  |  |
| Poor functional outcome- no. (%) | 1318 (76.9) | 902 (73.63) | 416 (85.07) | **<0.001** |
| mRS at discharge |  |  |  |  |
| Discharge mRS 0- no. (%) | 143 (8.34) | 115 (9.39) | 28 (5.73) | **<0.001** |
| Discharge mRS 1- no. (%) | 161 (9.39) | 136 (11.1) | 25 (5.11) | **<0.001** |
| Discharge mRS 2- no. (%) | 92 (5.37) | 72 (5.88) | 20 (4.09) | **<0.001** |
| Discharge mRS 3- no. (%) | 258 (15.05) | 184 (15.02) | 74 (15.13) | **<0.001** |
| Discharge mRS 4- no. (%) | 305 (17.79) | 223 (18.2) | 82 (16.77) | **<0.001** |
| Discharge mRS 5- no. (%) | 132 (7.7) | 93 (7.59) | 39 (7.98) | **<0.001** |
| Discharge mRS 6- no. (%) | 623 (36.35) | 402 (32.82) | 221 (45.19) | **<0.001** |
| Mortality |  |  |  |  |
| Death during admission- no. (%) | 623 (36.35) | 402 (32.82) | 221 (45.19) | **<0.001** |
| Death at 90 days- no. (%) | 714 (41.66) | 468 (38.2) | 246 (50.31) | **<0.001** |
| Death at 365 days- no. (%) | 834 (48.66) | 555 (45.31) | 279 (57.06) | **<0.001** |
| Length of stay |  |  |  |  |
| Days- median (IQR) | 8.00 (3.00-19.00) | 8.00 (3.00-19.00) | 8.00 (3.00-22.00) | 0.320 |
| Length of stay >14 days- no. (%) | 576 (33.61) | 393 (32.08) | 183 (37.42) | **0.035** |

Table S3: In-hospital analyses using multivariable logistic regression modelling. All models adjust for: adjusted for age, sex, pre-morbid modified Ranking Score, Oxfordshire Community Stroke Project classification, prevalent comorbidities on admission, and anticoagulant and antithrombotic medications on admission.

| Outcome | Primary model | SAP-adjusted model | NIHSS-adjusted model |
| --- | --- | --- | --- |
| Poor functional outcome | **1.58 (1.10-2.26), *P* = 0.013** | **1.49 (1.04-2.14), *P* = 0.031** | **1.49 (1.03-2.15), *P* = 0.033** |
| Death during admission | **1.47 (1.15-1.88), *P* = 0.002** | **1.44 (1.13-1.84), *P* = 0.004** | **1.44 (1.13-1.85), *P* = 0.004** |
| Length of stay >14 days | 1.23 (0.97-1.56), *P* = 0.081 | 1.17 (0.92-1.49), *P* = 0.195 | 1.17 (0.92-1.49), *P* = 0.199 |

Table S4: Multivariable Poisson regression models of in-hospital outcomes excluding all patients diagnosed with SAP (n included= 1520, 88.68% of total cohort). Models adjust for: adjusted for age, sex, NIHSS, pre-morbid modified Ranking Score, Oxfordshire Community Stroke Project classification, prevalent comorbidities on admission, and anticoagulant and antithrombotic medications.

| Outcomes | RR (95% CI) | *P*-value |
| --- | --- | --- |
| Poor functional outcome | 1.08 (1.01-1.17) | **0.031** |
| Death during admission | 1.23 (1.06-1.42) | **0.007** |
| Length of stay >14 days | 1.12 (0.95-1.32) | 0.190 |

Table S5: Multivariable Cox regression models of long-term mortality outcomes excluding all patients diagnosed with SAP (n included= 1520, 88.68% of total cohort). Models adjust for: adjusted for age, sex, NIHSS, pre-morbid modified Ranking Score, Oxfordshire Community Stroke Project classification, prevalent comorbidities on admission, and anticoagulant and antithrombotic medications.

| Outcomes | HR (95% CI) | *P*-value |
| --- | --- | --- |
| Death at 90 days | 1.19 (0.98-1.44) | 0.079 |
| Death at 365 days | 1.16 (0.97-1.38) | 0.112 |

Table S6: Multivariable Poisson regression models of in-hospital outcomes including only those patients with inflammatory biomarkers samples within 48 hours of admission (n included= 1248, 72.8% of the total cohort). Models adjust for: adjusted for age, sex, NIHSS, pre-morbid modified Ranking Score, Oxfordshire Community Stroke Project classification, prevalent comorbidities on admission, SAP, and anticoagulant and antithrombotic medications.

| Outcomes | RR (95% CI) | *P*-value |
| --- | --- | --- |
| Poor functional outcome | 1.09 (1.02-1.16) | **0.012** |
| Death during admission | 1.27 (1.09-1.49) | **0.002** |
| Length of stay >14 days | 1.10 (0.93-1.30) | 0.251 |

Table S7: Multivariable Cox regression models of long-term mortality outcomes including only those patients with inflammatory biomarkers samples within 48 hours of admission (n included= 1248, 72.8% of the total cohort). Models adjust for: adjusted for age, sex, NIHSS, pre-morbid modified Ranking Score, Oxfordshire Community Stroke Project classification, prevalent comorbidities on admission, SAP, and anticoagulant and antithrombotic medications.

| Outcomes | HR (95% CI) | *P*-value |
| --- | --- | --- |
| Death at 90 days | 1.33 (1.09-1.62) | **0.005** |
| Death at 365 days | 1.29 (1.07-1.54) | **0.006** |


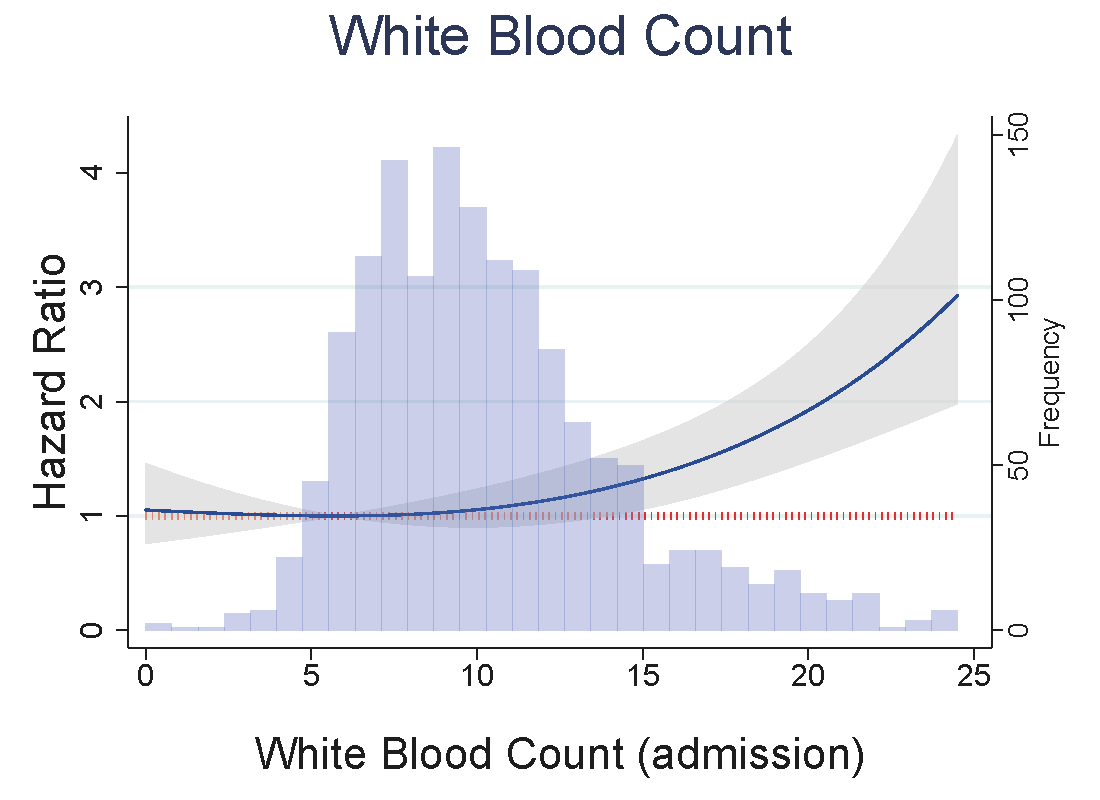


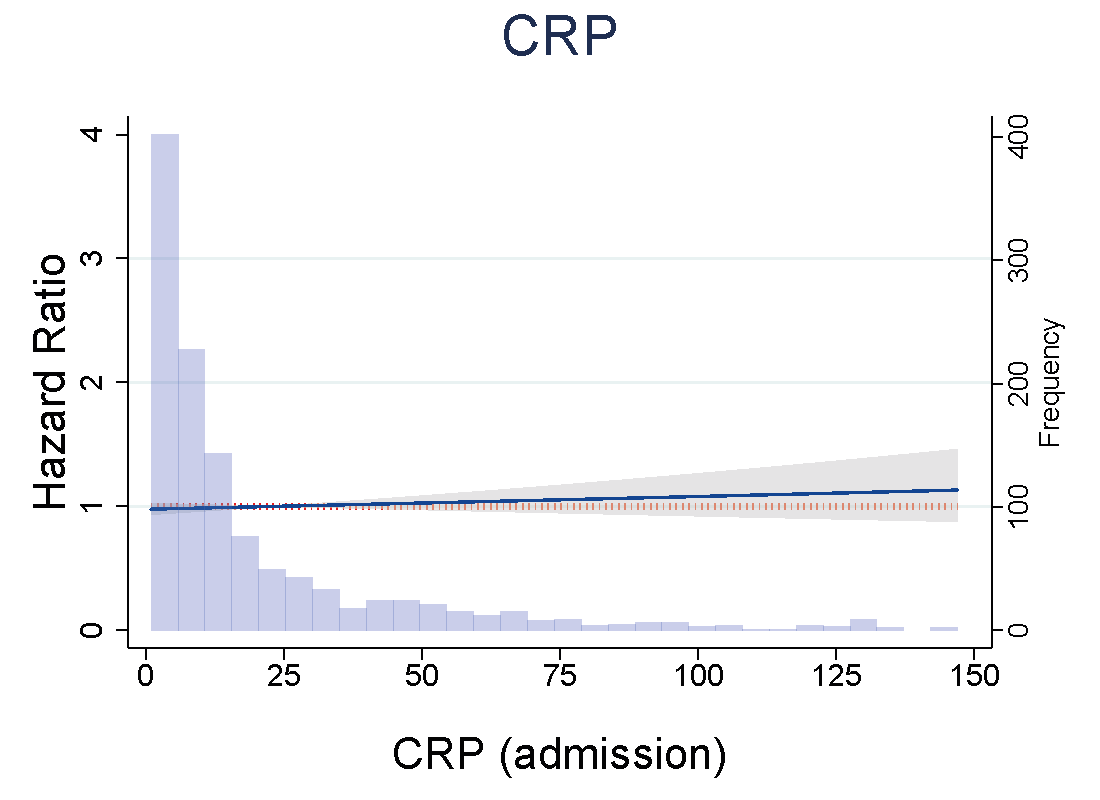


Supplementary Figure 1: Results of multivariable Cox regression sensitivity analyses assessing the association of white blood cell count (WCC) and C-reactive protein (CRP) as continuous variables, and long-term mortality after intracerebral hemorrhage, excluding all cases of SAP. As the relationship between WCC and mortality did not follow a linear pattern, this was parametrized using restricted cubic splines with 1 internal knot. The reference point (HR=1) for the HR function was chosen as the minimum value of the function, corresponding to a WCC value of 7.0x10^9^/L. There was no association between CRP and long-term mortality: HR (95% confidence interval) for 1-point increase in CRP = 1.0017 (0.9981-1.0052).

Hazard ratios and respective 95% confidence intervals are represented by the blue line with grey shadowing. The dotted red line represents the reference line (HR=1). The overlaying blue bar chart displays the distribution of each inflammatory marker in the included cohort. Model adjusted for age, sex, Oxfordshire Community Stroke Project classification, pre-morbid modified Rankin Scale, prevalent comorbidities on admission, admission antiplatelet and anticoagulant medication, and the National Institute of Health Stroke Scale.
